# Pediatric Capacity Building in Northern Laos: An Evaluation of the Pediatric Nursing Training Program at Lao Friends Hospital for Children

**DOI:** 10.1371/journal.pone.0349226

**Published:** 2026-07-15

**Authors:** Rebecca Silvers, Maly Chittaphai, Matt Evans, Jessica Van Meter

**Affiliations:** 1 Institute of Global Health Sciences, University of California, San Francisco, California, United States of America; 2 Lao Friends Hospital for Children, Luang Prabang, Lao PDR; 3 Vanderbilt School of Nursing, Nashville, Tennessee United States of America; Kwame Nkrumah University of Science and Technology College of Health Sciences, GHANA

## Abstract

**Background:**

In 2015, the child mortality rate in Laos was 53.6 per 1,000 live births. The Lao Friends Hospital for Children (LFHC) in Luang Prabang was established to enhance access to specialized pediatric care. To support workforce development, a Pediatric Nursing Training Program was implemented. However, the program has not undergone a formal evaluation since its inception. This study aims to evaluate the program’s first five years.

**Study design:**

The evaluation included a review of curriculum documents via an online platform and feedback collection through an online survey administered to former expatriate nurse volunteers who provided clinical mentorship. Additionally, semi-structured interviews were conducted with former nurse leaders who designed and implemented the program. Qualitative data were analyzed thematically and validated through triangulation.

**Results:**

The curriculum review revealed system-based and course-specific plans, including clear objectives, teaching methods, and evaluation tools. Of the 148 clinical mentors surveyed, a 45% response rate was achieved. Survey responses indicated improvements in nurses’ clinical skills since the program’s inception, with mentors emphasizing the importance of cultural attunement to Lao staff. Six out of seven former nurse leaders participated in interviews. Key themes emerged, highlighting the value of culturally relevant educational materials, multimodal teaching strategies, a train-the-trainer approach, and clinical mentorship as integral components of the program.

**Conclusion:**

The development of pediatric nursing specialization at LFHC was facilitated by structured curriculum plans, cultural sensitivity, diverse teaching methodologies, and robust clinical mentorship. These findings provide valuable insights for designing culturally competent and sustainable training programs in Laos and similar contexts.

## Introduction

Child mortality worldwide has declined substantially over recent decades. In 1990, approximately 93 children per 1,000 live births died before reaching age five (about 1 in 11), compared to 37 per 1,000 live births in 2022 (about 1 in 27) [1]. In 2022, an estimated 4.9 million children under the age of five died globally [1]. Despite this progress, significant regional disparities persist. Sub-Saharan Africa accounted for approximately half of all under-five deaths in 2022 and had an under-five mortality rate of about 74 deaths per 1,000 live births. Southern Asia accounted for roughly one quarter of global under-five deaths, with an under-five mortality rate of approximately 42 per 1,000 live births [1]. The vast majority of childhood deaths continue to occur in low- and middle-income countries [1,2].

Access to appropriate pediatric care is the largest determinant of child morbidity and mortality, and therefore, understanding access to care is essential to uncovering solutions worldwide [3]. For a country or region to provide access to pediatric health services, a system must optimize the number of clinicians and their clinical expertise, environmental and physical resources, and address patient variables and barriers to care. In addition, clinicians must be prepared with the clinical skills and knowledge to make appropriate clinical judgments in pediatric care [4].

There are numerous challenges in low- and middle-income countries (LMICs) to optimizing the nursing workforce to provide access to pediatric care. Additionally, high-quality professional nursing education in LMICs faces many challenges, including a lack of available faculty, limited clinical preceptorship hours, and failure of available evidence for best practice to apply to their clinical settings [5,6]. As a result, nurses struggle to apply what they have learned with available resources and standards of practice in their local setting, which results in a lack of standardized care and poor confidence in nursing practice [7]. Thirdly, the opportunities for specialized pediatric training are limited [8].

## Background

Laos, a landlocked country in Southeast Asia, is characterized by mountainous terrain and significant ethnic, religious, and linguistic diversity. Despite its rich cultural heritage, Laos faces considerable economic challenges and is classified by the World Bank as a low-income country. Its healthcare infrastructure is still developing, particularly in rural areas outside the capital city. To bridge gaps in healthcare delivery, various organizations in Laos partner with international aid agencies and non-governmental organizations (NGOs) to strengthen healthcare programs and enhance the education of medical professionals [9].

Efforts to improve healthcare in low- and middle-income countries (LMICs) like Laos have increasingly focused on supplementary education and training. Academic institutions and NGOs play a pivotal role in elevating clinical practice standards by providing specialized training for healthcare workers [10–13]. These initiatives aim to address critical shortages in healthcare workforce expertise and improve health outcomes in resource-limited settings.

In 2014, the non-governmental organization Friends Without Borders (FWAB), in collaboration with the Ministry of Health, launched an initiative to expand access to pediatric specialty care in northern Laos. This effort led to the establishment of a pediatric specialty medical center aimed at addressing critical healthcare gaps and reducing the child mortality rate, which was 53.6 per 1,000 live births. A key focus of the initiative was capacity building through comprehensive training programs for Lao healthcare professionals—including doctors, nurses, allied health workers, and administrative staff—to strengthen local expertise and improve health outcomes.

The Lao Friends Hospital for Children (LFHC) opened its doors in January 2015 as a no-cost tertiary pediatric referral hospital serving northern Laos. During its first five year, the hospital provided outpatient and inpatient pediatric care, emergency services, neonatal care, pediatric surgery, and basic critical care, and progressively expanded operating theatre capacity and emergency stabilization services in response to regional needs. As one of only two dedicated pediatric hospitals in the country, LFHC became a referral center for children requiring specialized medical and surgical care.

At its inception, the nursing workforce consisted entirely of newly graduated Lao nurses without prior pediatric specialization. These nurses entered a developing tertiary care environment that required rapid acquisition of pediatric knowledge, procedural competence, and clinical decision-making skills. The Pediatric Nursing Training Program was therefore integral not only to skill development but also to establishing foundational pediatric nursing standards within the hospital.

This program was designed to enhance the clinical competencies of nursing staff through culturally tailored didactic education and bedside clinical mentorship. The training program is supported by expatriate nurse leaders who commit to one- to two-year terms, as well as short-term nursing volunteers who provide bedside mentorship during their one- to twelve-month placements. Didactic sessions take place in the hospital’s designated learning spaces, with protected time allocated for nurses to participate in educational activities.

The pediatric nursing training program at LFHC is the first of its kind in Laos, yet a program evaluation has not been conducted. Thus, the purpose of this study was to evaluate the first five years of the specialized nursing training program. Aims including evaluating whether the program implemented culturally relevant training and increased nurses’ clinical knowledge and their ability to apply their knowledge within the clinical setting.

## Methods

### Study setting design

This study was conducted at the Lao Friends Hospital for Children (LFHC), a no-cost tertiary pediatric referral facility located in Luang Prabang, northern Laos. According to UNICEF estimates, the pediatric population in Laos is approximately 2.8 million; however, LFHC is one of only two dedicated pediatric hospitals in the country. Since opening its doors in 2015 with an outpatient clinic and inpatient wards, LFHC has expanded to provide a full range of specialized services, including surgery, neonatal, emergency, and critical care, a developmental clinic, and a thalassemia program. Annually, the hospital now serves more than 45,000 children from across northern Laos and has provided access to care for over 200,000 patients in the first ten years of operations. LFHC continues to train a growing workforce of Lao physicians and nurses, many of whom are new to pediatric specialty care.

### Study design

This study used a multi-method program evaluation design to assess the LFHC Pediatric Nursing Training Program implemented between 2015 and 2019. The evaluation incorporated three complementary data sources: a review of program curriculum and associated documents, an electronic survey of expatriate clinical mentors, and semi-structured interviews with former nurse leaders at LFHC. This approach allowed for triangulation of perspectives from individuals involved in program implementation, mentorship, and leadership.

Due to travel restrictions imposed during the COVID-19 pandemic, all evaluation activities were conducted remotely using online platforms. Surveys were designed and distributed through REDCap, a secure web-based platform for managing online surveys and databases. [14,15]. The survey remained open for participation from December 15, 2020, to February 1, 2021. Semi-structured interviews with former nurse leaders were conducted via Zoom between February and March 2021. Program curriculum documents and related materials were reviewed concurrently using a shared secure cloud-based document platform.

### Evaluation and theoretical framework

The evaluation was guided by the Context–Input–Process–Product (CIPP) evaluation framework, which is commonly used to assess educational and health workforce development programs. The CIPP framework supported a structured examination of the LFHC Pediatric Nursing Training Program by assessing the program context, the inputs used to support program development including curriculum and mentorship structures, the processes through which training and mentorship activities were implemented, and the perceived outcomes and sustainability of the program.

In addition, Madeleine Leininger’s Transcultural Theory of Nursing informed the conceptual understanding of cross-cultural mentorship and training within the program [16]. This theory emphasizes the importance of cultural awareness and culturally congruent care when working with diverse populations. The framework has been widely used in global health and resource-variable settings to guide culturally responsive health workforce development. Incorporating this theoretical perspective supported interpretation of how cultural context influenced mentorship relationships, training adaptation, and program sustainability.

### Sample selection and recruitment

Participants were purposively selected based on their direct involvement in the LFHC Pediatric Nursing Training Program between 2015 and 2019. Two participant groups were included: former LFHC clinical mentors and nurse leaders.

Clinical mentors were international nurse volunteers who served in mentorship roles, providing didactic education, bedside clinical teaching, and support to Lao nurses in applying knowledge to practice. Mentors also contributed to curriculum development and competency evaluation. Contact information for clinical mentors who volunteered at LFHC between 2015 and 2019 was obtained from the LFHC Human Resources Department and the Nursing Director. An introductory email was sent to potential participants explaining the study and inviting them to complete an online survey via a REDCap link.

Nurse leaders consisted of expatriate staff who worked at LFHC in Nursing Director or Nursing Educator roles. These individuals played a critical role in adapting and sustaining the program within the local context while residing in Laos and contributed to curriculum design, mentorship structures, and leadership development. All eligible nurse leaders were invited by the research team, in collaboration with the current Nursing Director, to participate in semi-structured interviews. Invitations were sent via email, asking interested participants to select an interview date and time.

This purposive sampling strategy ensured representation from individuals who directly designed, implemented, and guided the program, capturing perspectives central to evaluating its outcomes and sustainability.

### Data tools and collection

Data collection occurred simultaneously through a review of curriculum documents, a REDCap survey, and through semi-structured interviews over a four-week period. The LFHC Nurse Training Program curriculum and other relevant documents were gathered and reviewed to evaluate the following themes: program design, program objectives, training schedule, and the content of the program. Attention to the train-the-trainer model and cultural relevance was also noted.

The LFHC Clinical Mentor Survey consisted of three sections and was distributed in January 2021 to all expatriate nurses who participated in the clinical mentorship program from 2015 to 2019. The first section gathered information about participants’ nursing background, clinical experience prior to LFHC, and any prior experience working abroad. The second section focused on the expatriate nurses’ perspectives regarding the clinical skill set of the Lao staff nurses during their time at LFHC. Ratings were done based on recall of past experiences of direct observation. The final section provided an opportunity for self-reflection on the expatriate nurses’ contributions to the training process, specifically in clinical mentoring and cultural competence. A pilot test was conducted to enhance the face and content validity of the survey before it was distributed. All the data collected from the survey were imported via REDCap into a Microsoft Excel spreadsheet for analysis.

**Fig 1:**
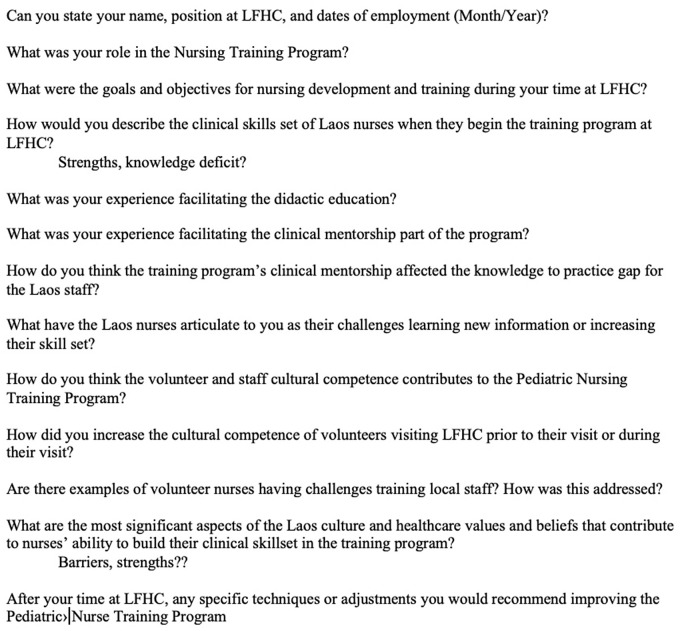
Interview Guide.

Interviews with former nurse leaders were scheduled at a time convenient for each participant. The interviews were conducted via Zoom and were recorded with the participants’ permission. Each interview was designed to last approximately one hour. The LFHC Program Evaluation Interview Guide was used for the semi-structured interviews and consisted of 14 questions (Fig 1). Two questions collected demographic data about the nurse leaders, while the remaining 12 questions focused on evaluating the program. These questions encouraged participants to reflect on their role in facilitating the LFHC Nurse Training Program. To ensure content and face validity, the interview guide was tested by the research team at Vanderbilt University. The interviews were recorded by the interviewer and submitted to Trint Ltd., an online high-quality and secure transcription service, to be transcribed [17].

### Data analysis

Data collected from the electronic REDCap survey were analyzed using descriptive statistics. Demographic variables were summarized as frequencies and percentages. Likert-scale responses were analyzed in Microsoft Excel using built-in functions (AVERAGE and STDEV) to calculate mean scores and standard deviations. For the qualitative component, interviews were audio-recorded, transcribed verbatim, and anonymized prior to analysis. Data were examined using thematic analysis informed by grounded theory methodology. Coding was performed manually by two members of the research team, followed by review with the Principal Investigator. The coding process involved open coding to identify initial concepts, axial coding to organize these into categories, and iterative refinement of emerging themes and subthemes. Discrepancies were resolved through consensus meetings, and an audit trail of coding decisions was maintained. Triangulation across coders and data sources enhanced credibility, and cross-validation of findings strengthened the rigor and trustworthiness of the analysis.

### Ethical considerations

This study received ethical approval from the Institutional Review Board (IRB) at Vanderbilt University under exempt (non-research) status (Protocol #202298). Additional approval to proceed with the study was granted by the Lao Friends Hospital for Children (LFHC) Research Committee following review of the study protocol. Participation in the study was entirely voluntary. Consent was implied through the completion of surveys and participation in interviews or focus groups, with participants informed of their right to withdraw at any time without penalty. No financial incentives or compensation were provided. All data were anonymized to safeguard confidentiality, and results are reported in aggregate to protect participant identity. Audio recordings of interviews and focus groups were obtained only with consent and remain securely stored, with assurance of non-disclosure beyond the research team.

## Results

### Curriculum documents review

The curriculum documents were stored on a privately accessed Microsoft OneDrive, with access controlled by the head of information technology at LFHC. The OneDrive contains organized folders, including audits, competency, courses, curricula, English vocabulary list, exams, in-services, medication safety, emergency simulations, nursing orientation, students, and training records, each with several subfolders. A review of the curriculum documents revealed thorough and well-organized record-keeping of clinical courses taught as part of the education and training programs since 2016. The training documents and PowerPoint presentations for each course were easily accessible in their designated subfolders. Lesson plans clearly outline the targeted learning objectives for each course, along with the associated evaluation methods, which include hands-on activities and group exercises. The clinical competency evaluations were clearly documented for each nurse at LFHC, but the schedule for student reassessment or evaluation was unclear. It was also unclear if there was testing to measure expatriate nurses’ understanding of the content that they were teaching.

### LFHC clinical mentorship survey

The LFHC Clinical Mentorship Survey was distributed to 148 former nursing volunteers who served from 2015 to 2019. Sixty-seven of the volunteers responded and completed the survey in full for a return rate of 45%.

#### Expatriate nurse demographics.

The first section of the survey focused on the demographics and work history of expatriate nursing volunteers (Table 1). A total of 67 survey responses were analyzed, revealing participation across all years of the Pediatric Nursing Training Program’s initial operation. The majority of respondents volunteered in 2017 (n = 19) and 2018 (n = 17), with additional participation in 2015 (n = 6), 2016 (n = 10), and 2019 (n = 15). Regarding the duration of service at LFHC, most respondents (64.2%) reported volunteering for two to six months (n = 43). Seventeen volunteers (25.4%) stayed for one month, five (7.5%) for six months to one year, and two (3%) for more than one year.

**Table 1:** Demographics of Participants: LFHC Clinical Mentorship Survey.

*Demographic Data (N = 67)*	
**Start participating at LFHC (Year)**	**n (%)**
2015	6 (9)
2016	10 (14.9)
2017	19 (28.4)
2018	17 (25.4)
2019	15 (22.4)
**Length of time at LFHC**	
< 1 month	17 (25.4)
2–6 months	43 (64.2)
6 months – 1 year	5 (7.5)
> 1 year	2 (3)
**Age (years)**	
21-30	32 (47.8)
31-39	14 (20.9)
40-49	5 (7.5)
50-59	11 (16.4)
> 59	5 (7.5)
**Home Country**	
Australia	21 (31.3)
United States	19 (28.4)
United Kingdom	11 (16.4)
Canada	8 (11.9)
New Zealand	3 (4.5)
Other ^a^	5 (7.5)
**Profession** ^**b**^	
Bedside Nurse	48 (71.6)
Clinical Nurse Specialist	9 (13.4)
Nurse Educator	8 (11.9)
Advance Practice Nurse (Nurse Practitioner)	5 (7.5)
Operating Room/Theater Nurse	3 (4.5)
Professor/Teacher at School of Nursing	2 (3)
Nurse Anesthetist	1 (1.5)
Other ^c^	6 (9)
**Clinical Experience (years)**	
1 - 5	17 (25.4)
5- 10	22 (32.8)
10-15	9 (13.4)
> 15	19 (28.4)
**Previous experience in resource-limited setting**	
Yes	41 (61.2)
No	26 (38.8)
**# countries experience working**	
None	26 (38.8)
1-3	30 (44.8)
3-10	9 (13.4)
> 10	2 (3)
**Clinical experience** ^**a**^	
Pediatric Ward	34 (50.7)
Emergency department (A & E)	26 (38.8)
Pediatric specific ED (A & E)	25 (37.3)
Pediatric Critical Care (PICU)	18 (26.9)
Pediatric Clinic	14 (20.9)
Neonatal Intensive Care	12 (17.9)
Pediatric Hematology & Oncology	11 (16.4)
Other ***	20 (29.9)

^a^Other includes Italy (n=2), Spain, Portugal, & Denmark.

^b^Participants were asked to mark all answers that apply.

^c^Other includes physician assistant, nurse manager (NICU), clinical nurse consultant (n = 2), research.

^d^Other includes stroke triage, PACU (pediatric recovery room) (n = 2), remote First Nations Primary Care Health Centre, Trauma, adult ICU (n = 3), adult medical & surgical wards (n = 7), school nurse in a public school, acute rehabilitation (n = 3), hospice, Operating Theatre (n = 3), Labor and Delivery (Maternity) (n = 2).

The participants represented a diverse range of countries, with the majority identifying as Australian (n = 21), American (n = 19), British (n = 11), Canadian (n = 8), or New Zealander (n = 3). Additional countries of origin included Italy (n = 2), Spain, Portugal, and Denmark (n = 1 each). Notably, 41 respondents (61%) had prior experience in resource-variable settings (RVS).

Forty-eight expatriate volunteers (71.5%) identified as bedside registered nurses, while others reported varied roles, including clinical nurse specialists (13.4%, n = 9), nurse educators (11.9%, n = 8), advanced practice nurses or nurse practitioners (7.5%, n = 5), operating room nurses (4.5%, n = 3), professors at university nursing schools (3%, n = 2), and nurse anesthetists (1.5%, n = 1).

Participants also had diverse clinical backgrounds. More than half (50.7%, n = 34) had experience in pediatric wards. Other areas of clinical experience included emergency departments (38.8%, n = 26), pediatric-specific emergency departments (27.3%, n = 25), pediatric critical care (26.9%, n = 18), pediatric clinics (20.9%, n = 14), neonatal intensive care (17.9%, n = 12), and pediatric hematology and oncology (16.4%, n = 11).

The respondents varied in their years of clinical nursing experience before joining LFHC: 17 (25.4%) had one to five years of experience, 22 (32.8%) had five to ten years, nine (13.4%) had 10–15 years, and 19 (28.4%) had over 15 years of experience.

#### Evaluation of the content of the training program.

In the second section of the LFHC Clinical Mentorship Survey, participants rated the Lao nursing staff on 14 skills using a Likert scale ranging from one to five where one referred to insufficient knowledge and being unable to complete the skill, while five referred to completing competence in knowledge and skill and being able to help support others (Table 2). The mean (standard deviation) of the clinical skill set rated by the participants in 2015 was 2.8 (0.4). Over the course of five years, the mean increased to 3.5 (0.5) in 2019. The greatest changes in mean scores between 2015 and 2019 were starting an IV live (1.1), recognition of a patient having a seizure (1), application of weight-based medication dosing (0.8), and application of appropriate respiratory support for hypoxia (0.8). The smallest change in mean scores was seen in the knowledge of normal vital signs for age (0.2), performing a 12-lead ECG (0.3), and recognition of a patient in respiratory distress or failure (0.3).

**Table 2:** LFHC Clinical Mentorship Survey: Nursing Clinical Skill Data by Year.

Nursing Clinical Skills	2015	2016	2017	2018	2019
Taking vital signs on a pediatric patient	3.3	3.7	3.6	3.6	3.8
Knowledge of normal vital signs patients of various ages	3	2.9	3.1	3.4	3.2
Drawing blood through a venipuncture	3.7	4	4	4.3	4.4
Starting an intravenous (IV) line to administer intravenous fluids/medications	3	4.1	3.9	4.1	4.1
Using a stethoscope to perform a respiratory examination	2.5	3.1	2.7	2.9	3.2
Application of weight-based medication dosing	2.7	2.8	2.7	3.1	3.5
Preparing and administering oral medications	3.2	3.7	3.5	3.6	3.9
Preparing and administering IV administration	2.8	3.5	3.3	3.4	3.5
Performing an electrocardiogram (EKG or ECG)	2.5	2.7	2.7	2.8	2.8
Recognition of hemodynamically unstable patient	2.3	2.6	2.5	2.8	2.8
Recognition of a patient in respiratory distress or failure	2.8	3.1	2.9	3.2	3.1
Application of the appropriate oxygenation delivery devise for hypoxemia/respiratory distress	2.7	2.8	2.9	3.2	3.5
Recognition of a child with seizures	2.3	3.1	3.1	3.2	3.3
Participation in pediatric or neonatal cardiopulmonary resuscitation (CPR)	2.7	2.9	2.8	3.1	3.3
**Mean**	**2.8**	**3.2**	**3.1**	**3.3**	**3.5**
**Standard Deviation**	0.4	0.5	0.5	0.5	0.5

#### Participants’ self-reflection on contributions to the training program.

The third section of the survey focused on the participants’ self-reflection on their contribution to the training process pertaining to clinical mentoring and cultural competence. The nurses responded to 14 statements on a Likert scale of one to five, where one was “strongly disagreed” and five was “strongly agree.” Eighty-four percent (n = 56) of participants agreed or strongly agreed that their knowledge of Lao culture contributed to their ability to provide clinical mentorship and education to the LFHC nursing staff at the bedside. The majority also agreed or strongly agreed (90%, n = 60) that participating in cultural events in the hospital and town increased their understanding go Lao values and beliefs. Additionally, 96% (n = 64) of volunteers responded that they agree or strongly agree that as their time at LFHC progressed, they could recognize barriers to learning in Lao and adapt their teaching methods.

Sixty-one percent (n = 41) of participants strongly agreed they were able to facilitate more constructive clinical mentorship as the strength of their relationship with the Lao staff strengthened (mean 4.5, SD 0.8). The participants agreed that they recognized the importance of cultural competence in the nursing training program (mean 4.6, SD 0.5). 96% (n = 64) of the participants strongly agreed or agreed that they understood the role of nursing in Laos at the end of their time in LFHC (mean 4.4, SD 0.6). Fifty-five percent of participants agreed or strongly disagreed with understanding the role of nursing in Laos prior to volunteering at LFHC (mean 3.5, SD 1.0).

There was neither an agreement or disagreement (mean 3.0, SD 1.0) that the Lao nurses struggled to apply their classroom education to the clinical setting, but 93% (n = 62) of the participants agreed or strongly agreed that they felt comfortable recognizing the knowledge to practice gap and addressing it as they mentored Lao staff at the bedside (mean 3.9, SD 0.6). Overall, the clinical mentors felt that their time working with the Lao nurses contributed to an increase in the clinical skills of the Lao staff (mean 3.9, SD 0.7).

### LFHC Program evaluation: Semi-structured interviews

#### Demographics.

During the first two years of the program at LFHC, a nursing director managed both the administrative and educational programs for the nurses, and in the latter three years, there was an additional role of a nurse educator (Participant 1). Semi-structured interviews were conducted with six former nurse leaders at LFHC. Two of the nurse leaders were from Australia; one was from the United Kingdom, one was from New Zealand, one was from Canada, and one was from the United States. The amount of time each nurse leader worked at LFCH ranged from nine months to two years. Based on the content analysis of the interview transcripts, many themes (italicized below) emerged related to the following topics: nursing curriculum development, clinical mentorship program, and cultural competence.

#### Nursing curriculum development.

*Continual Revision of Curriculum.* Participants described the evolution of the nursing curriculum as a dynamic and responsive process. In the early years of hospital development, training began with a brief WHO course, which several participants felt was too limited. One explained, “The initial training was only a short course based on the WHO’s little blue book” (Participant 2). Over time, the curriculum expanded with input from global sources. As another noted, “We adapted from WHO and MSF system-based curricula, but always tried to fit them to our context” (Participant 3).

The onboarding of a formal nurse educator in 2016 marked a turning point. According to one participant, “With a dedicated educator, we could customize the curriculum based on the Lao nurses’ skill set and what we had learned in the first two years” (Participant 1). This shift included the deliberate removal of irrelevant material. “We eliminated content written for high-income countries that did not make sense here” (Participant 4). Curriculum revision became a regular process, with each new educator reassessing practice and clinical goals. “Every one or two years, when a new educator joined, they looked at what the staff needed and adjusted the content” (Participant 1). Lao nurse educators also played a critical role once they began teaching independently. One participant highlighted, “They were encouraged to change the curriculum themselves, based on whether it met the needs of their colleagues” (Participant 5).

*Adaptive Teaching Style Grounded in Humility.* Alongside curriculum changes, participants emphasized the importance of adapting teaching styles to meet the learning preferences of Lao nurses. Early experiences revealed mismatches between Western teaching expectations and local educational norms. “The nurses were used to memorizing and repeating answers; they had never been asked to question or participate before” (Participant 5). To address this, educators experimented with different methods. One described, “We used a trial and error system to find what worked. Interactive games ended up being very effective” (Participant 1).

Tone and demeanor were also crucial. As one educator explained, “The nurses were very sensitive to the teacher’s tone and frustrations. They responded best to a compassionate, understanding style” (Participant 1). Participants consistently described the importance of humility in teaching, recognizing that respectful and culturally sensitive interactions were key to sustaining engagement.

*Creating a Culture of Critical Thinking.* A consistent theme across interviews was the need to move beyond rote memorization and cultivate critical thinking. Participants spoke about historical barriers. “Nursing education in Laos had been focused on memorization and tasks, not questioning or inquiry” (Participant 4). This approach left gaps in clinical reasoning. “The nurses were task compliant, but they did not always understand how interventions impacted the patient’s health” (Participant 3).

Several leaders provided striking examples. One recalled, “There were fundamental knowledge gaps, such as not connecting the pulse with the beating of the heart” (Participant 1). For the nurse leaders, teaching critical thinking was not only about knowledge but about transforming practice. As one summarized, “Our goal was to build clinical inquiry, so they could trust their assessments and contribute to planning care with the team” (Participant 4).

*Nursing Empowerment and Sustainability.* Another key focus was empowering Lao nurses to assume leadership roles as part of LFHC’s long-term sustainability plan. Early on, nurses struggled with confidence. “At first, they did not want to speak up or challenge norms; they stayed quiet even when they had good ideas” (Participant 3). Empowerment strategies included encouraging open communication. “We asked them to voice concerns and suggest improvements in patient care” (Participant 1).

A structured train-the-trainer model supported this goal. One leader explained, “We identified future nurse leaders by watching them over two years, looking at their clinical skills and emerging leadership” (Participant 1). Despite early barriers, including reluctance to stand out, progress was evident. “When one Lao nurse became an educational leader, it changed everything. It inspired others to step forward and use culturally appropriate teaching methods” (Participant 5).

#### Clinical mentorship program.

*Evolving Roles of Clinical Mentors.* Participants described how the role of clinical mentors adapted as Lao nurses gained competence. Early on, mentors were embedded directly in patient care to provide safety oversight and hands-on training. “At first, the mentors worked side by side with the nurses in every unit, making sure they were safe and guiding them step by step” (Participant 4). As local nurses advanced, mentors shifted their focus. “By late 2017, we had Lao shift leaders, and mentors were assigned to individuals identified as needing extra support” (Participant 4). Mentors rotated through inpatient, neonatal, operating theater, and emergency units, mirroring the schedules of Lao staff to maximize learning opportunities.

*Selecting and Preparing Mentors.* Nurse leaders highlighted that mentor selection was critical to program success. Volunteers were initially recruited through academic institutions in the United Kingdom, including the Liverpool School of Tropical Medicine (LSTM) offers a Diploma in Tropical Nursing for nurses wanting to work in LMICs. As one participant explained, “Many of our mentors came through the LSTM which provided them a good baseline knowledge for work with nurses here outside their home country” (Participant 2). Word of mouth also played a role. “A lot of our mentors were referrals from former volunteers” (Participant 3). Beyond clinical expertise, cultural humility emerged as an essential trait. “We looked for open-mindedness and adaptability, those who could adjust to Laos. Intensive care nurses often struggled here, but ER nurses thrived” (Participant 4).

*Supporting the Mentors through Cultural Humility.* Participants underscored the importance of preparing mentors to engage with humility and reflection. Preparation began with cultural orientation. “They received a pre-departure packet with Lao culture, their role, and details about LFHC” (Participant 4). On arrival, mentors shadowed seasoned volunteers and often spent time in villages to understand daily life. “They even had language learning time built into the schedule” (Participant 3).

Support also emphasized reflective practice. “We reminded mentors that they first needed to observe and learn the culture and workflow before suggesting changes” (Participant 3). Nurse leaders encouraged humility when navigating differences. “We told them, you are here to listen, to support, not to impose your way” (Participant 2). This reflective stance was reinforced through ongoing check-ins, debriefs, and monthly gatherings where mentors could process challenges and share insights.

*Staff Responses to Mentorship.* Initially, Lao staff were apprehensive. “At the bedside, they worried that having a mentor meant they were failing or being singled out” (Participant 1). Over time, perceptions shifted as mentors demonstrated respect and humility. “After explanations, they began requesting to be scheduled with mentors” (Participant 4). Trust deepened through personal relationships. “They invited mentors to weddings and dinners. They enjoyed those friendships” (Participant 4).

Cultural humility shaped how successful mentors interacted with staff. “The nurses were very sensitive to tone and attitude. Volunteers who were patient and humble were much more successful” (Participant 3). One participant added, “In Lao culture, you do not raise your voice. The mentors who adapted to that had better results” (Participant 4). Language barriers also underscored the need for humility. “Medical terms do not always translate. The best mentors admitted when things were unclear and worked together to find simpler explanations” (Participant 6). Ultimately, humility built trust. “The most successful mentors created safe relationships where nurses felt comfortable saying what they did not understand” (Participant 6).

#### Utilizing Cultural Humility within the LFHC Nursing Training Program.

*Respecting and Integrating Lao Cultural Practices.* All participants emphasized that cultural humility, defined as approaching local practices with openness and respect, was essential to developing the training program. As one nurse leader explained, “In Laos, post-birth customs require mothers to sit by the fire, and colostrum is believed to harm infants. We needed to understand these practices before we could teach neonatal care” (Participant 6). Nurses also navigated requests for traditional healing. “A family wanted to take their child home for a ceremony that they believed would cure pneumonia. We respected their wishes and provided antibiotics for their journey, orally, and intravenously when they returned” (Participant 1). End-of-life care further demonstrated the importance of humility. “Families often took children home to die, because of spiritual beliefs about spirits not returning. It was important that we respected that, even if it conflicted with our own norms” (Participant 3).

*Adapting Global Curricula with Humility.* Participants reflected on the limitations of directly applying international curricula without considering local realities. “When volunteers came to teach WHO’s ETAT course, it did not work. The content was at the wrong level, language was a barrier, and some lessons clashed with local practices” (Participant 1). Over time, the team recognized the need for humility in training design. “We rewrote every course specifically for LFHC, taking into account culture, language, and the actual diseases here” (Participant 1). Continuous adaptation and openness to feedback from Lao nurses ensured that curricula remained relevant, effective, and grounded in local context.

*Practicing Humility as an Ongoing Process.* Participants emphasized that cultural humility was not a single adjustment but a continuous process of reflection and adaptation. One explained, *“Cultural humility meant reminding ourselves to keep learning. Every time we thought we understood, something new challenged our assumptions”* (Participant 2). Another reflected, *“The most important part was not assuming we knew better. We had to listen first, and then work together to find solutions”* (Participant 4). Humility was therefore seen as a mindset that required sustained openness to learning and adaptation in everyday interactions.

## Discussion

### Key findings

The LFHC Nursing Training Program led to a notable improvement in the Lao nurses’ clinical skillset, as consistently emphasized by both clinical mentors and nurse leaders. In addition to strengthening procedural competence, the program facilitated a shift toward more advanced forms of clinical reasoning and problem-solving. Participants described how nurses began to move beyond rote memorization toward applying knowledge in practice, asking questions, and critically engaging with patient care. These changes reflect a transition from task-based nursing to a more holistic and critical thinking approach to clinical practice.

A central factor in this development was the adaptation of the curriculum to the Lao context. By eliminating materials designed for high-income countries and customizing training to reflect local epidemiology, cultural practices, and available resources, the LFHC team ensured that the curriculum was both accessible and relevant. Such contextualization is consistent with literature on global health education, which emphasizes that externally imported curricula, without adaptation, often fail to achieve meaningful practice change [18–20]. The WHO ETAT course, for example, was attempted at LFHC but was reported to be of limited value without curriculum adaptations due to its misalignment with Lao nursing practice and cultural norms.

The program’s combined strategy of structured didactic sessions and hands-on clinical mentorship was another key contributor to success. Bedside mentorship created a feedback loop in which nurses could immediately apply knowledge, receive guidance, and reflect on practice. Nurse leaders noted that mentorship also facilitated psychosocial support, helping Lao nurses develop confidence and autonomy in their roles. Importantly, the clinical mentorship model was not static. Over time, it shifted from broad support of all staff to more targeted mentorship for junior nurses, allowing for efficient use of mentor expertise.

A further strength of the LFHC program was its investment in sustainability through leadership development. The modified train-the-trainer approach was pivotal in building long-term capacity. By the second year, a Lao nurse was identified as a future educator, and by 2019, this nurse was independently teaching the core curriculum. This not only ensured program continuity but also signaled a critical shift in power and ownership from expatriate staff to Lao leadership. In qualitative accounts, this transition was described as a turning point that inspired other nurses to aspire to leadership roles. Such findings align with global evidence that local leadership and peer-to-peer teaching are essential for enduring health system strengthening [21].

Perhaps most importantly, the LFHC experience demonstrated that cultivating critical thinking among nurses is possible even in traditionally rote-based educational systems. Through compassionate teaching, interactive learning strategies, and culturally sensitive mentorship, Lao nurses developed the ability to analyze clinical situations, anticipate complications, and contribute actively to care planning. This represents a substantial departure from historical models of nursing education in LMICs and highlights the potential for global nursing initiatives to drive pedagogical change.

Taken together, the findings of this evaluation underscore the importance of three interdependent elements in global nursing capacity building: contextual adaptation of curricula, integration of bedside mentorship, and investment in leadership through train-the-trainer strategies. The LFHC model demonstrates that when these elements are combined, nurse training can advance beyond technical skill acquisition to foster critical thinking, confidence, and sustainable leadership.

### Volunteer perspectives and bidirectional learning

A significant finding from this study was the difference in the mean scores reflecting volunteers’ knowledge of the role of Lao nursing before and after participation at LFHC (3.5 vs. 4.4). This increase demonstrates that international clinical mentors not only contributed to the strengthening of Lao nurses’ clinical skills but also deepened their own understanding of the cultural, clinical, and leadership roles of local nurses. This highlights the reciprocal nature of the program, in which mentorship fostered bidirectional learning and exchange. Importantly, it underscores the value of cultural humility in cross-cultural partnerships, as volunteers gained greater awareness of the Lao context and the complexities of nursing practice in a resource-variable setting. Such findings reinforce the importance of designing global mentorship initiatives as bidirectional learning opportunities that promote both local capacity building and the professional development of international collaborators.

### Implications for global nursing education

The evaluation of the LFHC Nursing Training Program provides important lessons for nursing education in resource-variable settings. First, the findings reinforce that standardized, externally developed training programs may not achieve their intended outcomes without meaningful cultural and contextual adaptation. Efforts to implement global curricula such as WHO’s ETAT course highlight this limitation, as the program was perceived as misaligned with local needs and practices. In contrast, the LFHC model demonstrated that tailoring content to reflect local learning styles, language, and health system realities can lead to measurable gains in both clinical skills and critical thinking.

Second, the integration of bedside mentorship alongside didactic learning proved critical. This aligns with growing evidence that experiential learning models are particularly effective for bridging knowledge-to-practice gaps in nursing. Mentorship not only improved technical competencies but also fostered professional identity and autonomy, qualities that are central to the advancement of nursing as a profession globally. For nurses in Laos, who traditionally trained in hierarchical and didactic systems, the opportunity to engage in dialogue, ask questions, and apply critical reasoning marked a transformative educational shift.

Finally, the study underscores the importance of embedding cultural humility into global nursing education. Expatriate mentors and educators at LFHC recognized that success depended on listening to Lao nurses’ perspectives, respecting local practices, and adapting pedagogical approaches accordingly. This emphasis on cultural humility rather than cultural competence promoted a spirit of mutual learning and helped establish trust, which was repeatedly highlighted in interviews as central to the program’s success.

### Sustainability and leadership development

A key contribution of the LFHC program was its explicit focus on sustainability. Too often, nursing education initiatives are limited by dependence on external trainers or short-term funding. In contrast, LFHC prioritized developing local leadership capacity through a modified train-the-trainer approach. By the second year of the program, a Lao nurse was formally identified as an educator, and within three years, they assumed independent responsibility for delivering the curriculum. This transition not only secured the program’s continuity but also elevated the professional role of nurses within the hospital.

Leadership development extended beyond the formal educator role. Qualitative findings revealed that exposure to mentorship and responsibility inspired many Lao nurses to envision themselves as leaders in their units. Several participants reflected that the program encouraged them to mentor junior colleagues, set standards for practice, and advocate for patient care improvements. These findings echo global nursing literature that emphasizes the link between local leadership and sustainable improvements in care quality.

The emphasis on sustainability also offers insights for broader health systems. By empowering Lao educators and fostering critical thinking among staff, the LFHC program contributed to resilience during crises, including the COVID-19 pandemic, when international travel restrictions severely limited expatriate support. In this way, the program illustrates how investments in LMIC nursing leadership can buffer against system shocks and promote long-term independence from external aid.

### Broader global health implications

Beyond the context of LFHC, this evaluation contributes to the discourse on how global partnerships can advance health equity. It illustrates the value of bi-directional learning, where expatriate mentors adapted their teaching to local contexts while simultaneously learning from Lao nurses about culturally grounded care practices [22]. Such partnerships advance not only technical skills but also relational capacity, which is critical for sustainable health system strengthening.

Furthermore, the LFHC experience provides a replicable model for other low- and middle-income countries seeking to strengthen pediatric nursing education. The combination of contextualized curricula, mentorship, and leadership development could be adapted for various clinical areas, from emergency and critical care to maternal and newborn health.

### Strengths and limitations of the study

The strengths of this project include strong engagement and participation from nursing leadership at LFHC. The clinical mentorship survey achieved a response rate of 45 percent, and six of the seven former nurse leaders from LFHC participated in semi-structured interviews, ensuring a broad representation of leadership perspectives. The use of a mixed-methods approach further strengthened the evaluation by helping to mitigate potential deficiencies and biases associated with reliance on a single method [23].

This study also has several limitations. A primary limitation was recall bias, as participants were asked to reflect on past work experiences [24]. Social desirability bias may have further influenced responses, particularly in relation to sensitive topics such as leadership and training effectiveness. The COVID-19 pandemic imposed additional challenges, including the absence of a standardized, validated survey tool, reliance on online technologies with varying levels of internet connectivity, and the inability to conduct in-person data collection. Lastly, due to travel restrictions during the pandemic, feedback from Lao staff could not be incorporated, limiting the inclusion of their perspectives, which are essential for a comprehensive evaluation of the LFHC Pediatric Nursing Training Program. Despite these limitations, the study provides critical insights into the development, implementation, and sustainability of pediatric nursing education and mentorship at LFHC, offering lessons that may be transferable to education program development in similar resource-variable settings.

### Recommendations for further research

This study provides evidence for new approaches to building and evaluating sustainable and culturally relevant nursing training programs in resource-variable settings. The lessons learned from this project can inform future capacity-building initiatives by highlighting techniques to identify potential barriers early and equipping program developers with the tools necessary to ensure program success.

Future research is needed in several areas. First, further studies should evaluate the long-term outcomes of train-the-trainer models, particularly regarding how they influence nurse retention, leadership development, and patient outcomes. Second, research should focus on refining methods for integrating cultural humility, which must be a central component of any cross-cultural partnership, into training curricula. This includes evaluating how cultural humility practices affect learner engagement, mutual respect, and the sustainability of acquired skills. Third, there is a need for comparative studies examining how different mentorship models, such as in-person, virtual, or hybrid, affect knowledge translation and practice change across resource-variable contexts. Finally, involving nurses in LMIC’s perspectives, which were limited in this study due to COVID-19 restrictions, will be crucial for gaining a more comprehensive understanding of training needs, effectiveness, and sustainability.

## Conclusion

The LFHC Nursing Training Program demonstrated that tailored, context-specific approaches can strengthen nursing skills, promote critical thinking, and foster leadership development in resource-variable settings. The use of bedside mentorship, didactic teaching, and a train-the-trainer model provided a sustainable pathway for building local capacity while advancing nursing leadership within LFHC. Importantly, the study underscores that cultural humility must be a central pillar in any cross-cultural training partnership. By approaching collaboration with openness, mutual respect, and recognition of everyone’s knowledge, training programs can achieve greater relevance, sustainability, and impact.

Future initiatives should not only focus on clinical skill-building but also prioritize the integration of cultural humility into program design and evaluation. Doing so will help ensure that global nursing partnerships remain equitable, contextually appropriate, and capable of driving lasting improvements in patient care and health system strengthening.

## Supporting information

S1 File LFHC Volunteer Survey: Program Feedback Likert-Score Results. (XLS)

S2 File LFHC Volunteer Sruvey: Knowledge and Skill Assessment Results. (XLSX)

## References

[1] United Nations Children’s Fund (UNICEF). Under five mortality. https://data.unicef.org/topic/child-survival/under-five-mortality/. 2018. Accessed 2025 June 2.

[2] Liu L, Oza S, Hogan D, Perin J, Rudan I, Lawn JE, et al. Global, regional, and national causes of child mortality in 2000-13, with projections to inform post-2015 priorities: An updated systematic analysis. Lancet. 2015;385(9966):430–40. doi: 10.1016/S0140-6736(14)61698-6 25280870

[3] Rutherford ME, Dockerty JD, Jasseh M, Howie SRC, Herbison P, Jeffries DJ, et al. Access to health care and mortality of children under 5 years of age in the Gambia: A case-control study. Bull World Health Organ. 2009;87(3):216–24. doi: 10.2471/blt.08.052175 19377718 PMC2654650

[4] Kissoon N, Burns J. Who should get pediatric intensive care when not all can? A call for international guidelines on allocation of pediatric intensive care resources*. Pediatr Crit Care Med. 2014;15(1):82–3. doi: 10.1097/PCC.0000000000000038 24389710

[5] World Health Organization. WHO global disability action plan 2014–2021: Better health for all people with disability. Geneva: WHO. https://www.who.int/disabilities/actionplan/en/

[6] Wong FKY, Liu H, Wang H, Anderson D, Seib C, Molasiotis A. Global nursing issues and development: Analysis of World Health Organization documents. J Nurs Scholarsh. 2015;47(6):574–83. doi: 10.1111/jnu.12174 26488137

[7] Morin KH. Evolving global education standards for nurses and midwives. MCN Am J Matern Child Nurs. 2012;37(6):360–4; quiz p.365-6. doi: 10.1097/NMC.0b013e31825df7e7 22976823

[8] Slusher TM, Kiragu AW, Day LT, Bjorklund AR, Shirk A, Johannsen C, et al. Pediatric critical care in resource-limited settings-overview and lessons learned. Front Pediatr. 2018;6:49. doi: 10.3389/fped.2018.00049 29616202 PMC5864848

[9] Pacific Bridge Medical. Healthcare in Laos. https://www.pacificbridgemedical.com/publication/healthcare-in-laos/. 2017. Accessed 2025 June 2.

[10] Barron A-M, Moran J, Nina SS, Harlow J, Gyawali M, Hossain F, et al. Building specialized nursing practice capacity in Bangladesh: An educational program to prepare nurses to care for oncology and bone marrow transplant patients in Dhaka, Bangladesh. J Glob Oncol. 2018;4:1–6. doi: 10.1200/JGO.2016.006486 30222084 PMC6223378

[11] Halabi JO, Abdalrahim MS, Persson GL, Hedemalm A, Lepp M. The development of a preceptor training program on clinical nursing education in Jordan in collaboration with Sweden. J Contin Educ Nurs. 2012;43(3):135–44. doi: 10.3928/00220124-20111115-04 22106880

[12] Kumar R, Canarie MF. Developing pediatric critical care in Kenya. Pediatr Crit Care Med. 2019;20(12):e538–45. doi: 10.1097/PCC.0000000000002130 31805021

[13] McConnell KA, Krisher LK, Lenssen M, Bunik M, Bunge Montes S, Domek GJ. Telehealth to expand community health nurse education in rural guatemala: A Pilot feasibility and acceptability evaluation. Front Public Health. 2017;5:60. doi: 10.3389/fpubh.2017.00060 28405582 PMC5370395

[14] Harris PA, Taylor R, Thielke R, Payne J, Gonzalez N, Conde JG. Research electronic data capture (REDCap)--a metadata-driven methodology and workflow process for providing translational research informatics support. J Biomed Inform. 2009;42(2):377–81. doi: 10.1016/j.jbi.2008.08.010 18929686 PMC2700030

[15] Harris PA, Taylor R, Minor BL, Elliott V, Fernandez M, O’Neal L, et al. The REDCap consortium: Building an international community of software platform partners. J Biomed Inform. 2019;95:103208. doi: 10.1016/j.jbi.2019.103208 31078660 PMC7254481

[16] Leininger MM. Leininger’s theory of nursing: cultural care diversity and universality. Nurs Sci Q. 1988;1(4):152–60. doi: 10.1177/089431848800100408 3205480

[17] Trint Ltd. Trint Platform. https://www.trint.com/. Accessed 2020.

[18] Canarie MF, Shenoi AN. Teaching the principles of pediatric critical care to non-intensivists in resource limited settings: Challenges and opportunities. Front Pediatr. 2018;6:44. doi: 10.3389/fped.2018.00044 29552547 PMC5840157

[19] Ralston ME, Day LT, Slusher TM, Musa NL, Doss HS. Global paediatric advanced life support: Improving child survival in limited-resource settings. Lancet. 2013;381(9862):256–65. doi: 10.1016/S0140-6736(12)61191-X 23332963

[20] Shilkofski N, Hunt EA. Identification of barriers to pediatric care in limited-resource settings: A simulation study. Pediatrics. 2015;136(6):e1569-75. doi: 10.1542/peds.2015-2677 26553183

[21] Bell SA, Bam V, Acheampong E. Developments in emergency nursing education in Ghana. Emerg Nurse. 2015;23(8):18–21. doi: 10.7748/en.23.8.18.s24 26638754

[22] Sullivan BJ, Bettger JP. Community-informed health promotion to improve health behaviors in honduras. J Transcult Nurs. 2018;29(1):14–20. doi: 10.1177/1043659616670214 27671172

[23] Thurmond VA. The point of triangulation. J Nurs Scholarsh. 2001;33(3):253–8. doi: 10.1111/j.1547-5069.2001.00253.x 11552552

[24] Melnyk BM, Fineout-Overholt E. Evidence-based practice in nursing & healthcare: A guide to best practice. 4th ed. Philadelphia: Wolters Kluwer. 2019.

