# Supplementary material for: Pediatric Capacity Building in Northern Laos: An Evaluation of the Pediatric Nursing Training Program at Lao Friends Hospital for Children
Source: PLoS One. 2026 Jul 15;21(7):e0349226. doi: 10.1371/journal.pone.0349226 (PMC13372149; doi:10.1371/journal.pone.0349226)
Supplement: Attachment — Submitted filename: Response to Reviewers.pdf [file pone.0349226.s007.pdf]

UCSF Institute for Global Health Sciences | Center for Global Nursing

August 20th, 2025

Dear PLOS ONE Academic Editor and Reviewers,

We are grateful for the time and thoughtful feedback provided during the review of our manuscript. We have carefully revised the text to address all comments and believe these revisions have strengthened the clarity, rigor, and contribution of our work. Below we provide a point-by-point response, with reviewer comments in italics followed by our responses regarding the following manuscript:

Pediatric Capacity Building in Northern Laos: An Evaluation of the Pediatric Nursing Training Program at Lao Friends Hospital for Children

---

Methods

1. Study setting and design
Comment: Please include a description of the LFHC setting (level of hospital, services rendered, staff strength, staff caliber, and possibly child mortality figures).
Response: We have added a description of LFHC as a tertiary pediatric referral hospital in Luang Prabang, Laos. The revised text now includes information on the types of services provided, the scope of staff roles, and its role in serving a vulnerable pediatric population.

---

2. Sample selection and recruitment
Comment: Certain sentences belong in other sections (voluntary participation → ethics, pilot test → validity, interview details → data collection).
Response: We have reorganized these elements into appropriate sections. Voluntary participation and confidentiality are now described under Ethical Considerations. Details of the pilot survey are presented under Tools, and interview procedures are included in the Data Collection subsection.

---

3. Data analysis
Comment: More detail is needed on quantitative (means, SDs) and qualitative (grounded theory, coding, theme identification, anonymization) analysis.
Response: We have expanded our description of both quantitative and qualitative data analysis. For the quantitative data, we clarify that descriptive statistics (means, SDs, and percentages) were calculated using Microsoft Excel. For qualitative data, we elaborate on the grounded theory approach, including the coding process, identification of themes, triangulation by multiple reviewers, and anonymization of transcripts.

Ethical Considerations

Comment: Clarify "non-research" IRB status, type of approval from LFHC, and include details on participation, right to withdraw, risks/benefits, compensation, confidentiality, and data protection.
Response: We have clarified that the Vanderbilt University IRB reviewed this project and granted it "exempt" status, which is a formal university designation for program evaluations that do not involve biomedical interventions or place participants at risk. This status indicates that the project does not require ongoing IRB monitoring but is nonetheless subject to university oversight and human subjects' protection standards. In addition, the LFHC Research Committee independently reviewed and approved the study protocol. The revised section now explicitly outlines voluntary participation, right to withdraw, absence of financial incentives, anonymity, confidentiality safeguards, and secure storage of recordings.

Results

1. Likert scale clarification

Comment: Clarify if Likert ratings were by direct observation, assessment, or recall.
Response: We now specify that Likert ratings were completed by expatriate clinical mentors based on their direct observation of nurses' skills during training and practice, with some recall bias acknowledged.

2. Mean score changes (Table 2)

Comment: Explain improvements and declines in specific skills; discuss significance of change from 3.5 → 4.4.
Response: We have expanded the Results and Discussion sections to reflect that improvements in mean scores demonstrate not only increased clinical skills but also enhanced critical thinking among Lao nurses. We also added a discussion of the significant increase in mentors' self-reported knowledge of the Lao nursing role (3.5 → 4.4).

3. Potential biases

Comment: Expatriate mentors may have biased program ratings.
Response: We acknowledge this limitation explicitly in the Discussion, noting that expatriate mentors both delivered and evaluated the program, which may have influenced objectivity. To mitigate this, we used multiple data sources (surveys, interviews, curriculum review) to triangulate findings.

4. Self-reflection section

Comment: Provide graphical presentation, analyze influence of changes, link to skills, discuss social desirability bias.
Response: We appreciate this suggestion. While we considered adding a graphical presentation, we determined that the small sample size and exploratory nature of the data limited the utility of additional figures. Instead, we have clarified in the manuscript that the changes in self-reflection responses most likely reflect an increased cultural understanding of Lao nursing roles, though these findings cannot be directly linked to skill-based outcomes presented in Table 2. We

therefore avoid overinterpretation of the results. To address the reviewer's concern about social desirability bias, we have expanded the Discussion to note this as a potential limitation and outlined the measures taken to reduce its impact, including the use of anonymized surveys, voluntary participation, and assurances of confidentiality.

5. Semi-structured interviews
Comment: Present findings in qualitative structure with quotes, not narration.
Response: The section has been significantly revised to highlight key themes supported by participant quotes, presenting findings in a qualitative structure to better reflect interview data.

Discussion

1. Key findings
Comment: Expand discussion of cultural immersion and link to transcultural nursing theory.
Response: We added content linking cultural immersion of mentors to the principles of transcultural nursing theory, emphasizing how cultural humility was integral to program success.

2. Significant difference in mean scores (3.5 vs. 4.4)
Comment: Discuss this difference.
Response: Addressed as noted above, highlighting the significance of this increase in enhancing mentors' understanding of the Lao nursing role.

3. Limitations and biases
Comment: Address recall bias, expatriate role, technology issues, lack of Lao staff voice, etc.
Response: These have all been integrated into the expanded Limitations section.

4. Further research
Comment: Suggest areas for future study.
Response: A new subsection recommends further research on culturally relevant, sustainable training models in resource-variable settings, with explicit inclusion of cultural humility in cross-cultural partnerships.

We sincerely thank the reviewers and editor for their valuable comments, which have significantly improved the quality of our manuscript. We hope the revisions and clarifications provided meet expectations and respectfully resubmit our manuscript for your consideration.

Sincerely,

Rebecca Silvers DNP, APN, CPNP-AC, CCRN, RNFA
On behalf of all co-authors
