# Supplementary material for: Pediatric Capacity Building in Northern Laos: An Evaluation of the Pediatric Nursing Training Program at Lao Friends Hospital for Children
Source: PLoS One. 2026 Jul 15;21(7):e0349226. doi: 10.1371/journal.pone.0349226 (PMC13372149; doi:10.1371/journal.pone.0349226)
Supplement: Attachment — Submitted filename: PLOS ONE Letter March 2026.pdf [file pone.0349226.s009.pdf]

UCSF Institute for Global Health Sciences
Center for Global Nursing

March 4, 2026

PLOS ONE Editorial Office

Re: **Manuscript ID: PONE-D-25-29935_R1**
Title: *Pediatric Capacity Building in Northern Laos: An Evaluation of the Pediatric Nursing Training Program at Lao Friends Hospital for Children*

Dear Editor and Reviewers,

We sincerely thank the reviewers for their careful evaluation of our manuscript and for the positive feedback regarding the technical rigor, statistical analysis, and clarity of the work. We appreciate the opportunity to revise the manuscript and have addressed the reviewer comments as outlined below.

Response to Reviewer Comments
We note that Reviewer #2 indicated that all comments had been addressed, and Reviewer #3 provided feedback on the paper as well as a final suggestion requesting clarification of the evaluation model used in the study. In response, we have made the following revisions:

1. **Formatting adjustments**
   The manuscript formatting was revised to improve clarity and alignment with journal requirements. Numerical values previously associated with section headings have been removed to ensure consistency with the journal's formatting guidelines.
2. **Language and spelling corrections**
   The manuscript was carefully reviewed to address spelling, typographical, and minor grammatical errors. Corrections were made throughout the manuscript to improve readability and ensure the text is presented clearly in standard English.
3. **Clarification of the evaluation framework**
   In response to Reviewer #3's suggestion to clarify the model used to guide the evaluation, we have added a description of the Context–Input–Process–Product (CIPP) evaluation framework within the Methods section. The framework is now explicitly described as guiding the evaluation of the program context, educational inputs including curriculum and mentorship structures, implementation processes, and program outcomes. Incorporating this framework strengthens the methodological transparency of the study and clarifies the structure used to guide the evaluation and interpretation of findings.

All revisions have been incorporated into the revised manuscript and are reflected within the Methods section.

We thank the reviewers again for their thoughtful feedback and for their positive assessment of the manuscript. We appreciate the opportunity to improve the clarity of our work and believe the revisions have strengthened the methodological transparency and presentation of the study. We

hope the revised manuscript is now suitable for publication in PLOS ONE and we look forward to your consideration**.**

Sincerely,

Rebecca Silvers
On behalf of all authors
