# Supplementary material for: Pediatric Capacity Building in Northern Laos: An Evaluation of the Pediatric Nursing Training Program at Lao Friends Hospital for Children
Source: PLoS One. 2026 Jul 15;21(7):e0349226. doi: 10.1371/journal.pone.0349226 (PMC13372149; doi:10.1371/journal.pone.0349226)
Supplement: Attachment — Submitted filename: PONE-D-25-29935_R1.docx [file pone.0349226.s006.docx]

PLOS ONE

Pediatric Capacity Building in Northern Laos: An Evaluation of the Pediatric Nursing

Training Program at Lao Friends Hospital for Children

--Manuscript Draft--

| Manuscript Number: | PONE-D-25-29935R1 |
| --- | --- |
| Article Type: | Research Article |
| Full Title: | Pediatric Capacity Building in Northern Laos: An Evaluation of the Pediatric Nursing Training Program at Lao Friends Hospital for Children |
| Short Title: | Pediatric Nursing Capacity Building in Northern Laos |
| Corresponding Author: | Rebecca Silvers, DNP, APN, CPNP-AC  University of California San Francisco  San Francisco, CA UNITED STATES OF AMERICA |
| Keywords: | global nursing; knowledge to practice gap; pediatric acute care; nurse training program; clinical mentorship; cultural humility |
| Abstract: | Background: In 2015, the child mortality rate in Laos was 53.6 per 1,000 live births. The Lao Friends Hospital for Children (LFHC) in Luang Prabang was established to enhance access to specialized pediatric care. To support workforce development, a Pediatric Nursing Training Program was implemented. However, the program has not undergone a formal evaluation since its inception. This study aims to evaluate the program’s first five years.  Study Design: The evaluation included a review of curriculum documents via an online platform and feedback collection through an online survey administered to former expatriate nurse volunteers who provided clinical mentorship. Additionally, semistructured interviews were conducted with former nurse leaders who designed and implemented the program. Qualitative data were analyzed thematically and validated through triangulation.  Results: The curriculum review revealed system-based and course-specific plans, including clear objectives, teaching methods, and evaluation tools. Of the 148 clinical mentors surveyed, a 45% response rate was achieved. Survey responses indicated improvements in nurses’ clinical skills since the program’s inception, with mentors emphasizing the importance of cultural attunement to Lao staff. Six out of seven former nurse leaders participated in interviews. Key themes emerged, highlighting the value of culturally relevant educational materials, multimodal teaching strategies, a train-thetrainer approach, and clinical mentorship as integral components of the program. Conclusion: The development of pediatric nursing specialization at LFHC was facilitated by structured curriculum plans, cultural sensitivity, diverse teaching methodologies, and robust clinical mentorship. These findings provide valuable insights for designing culturally competent and sustainable training programs in Laos and similar contexts. |
| Order of Authors: | Rebecca Silvers, DNP |
|  | Maly Chittaphai |
|  | Madeleine Wong, MS |
|  | Matt Evans, MS, MPH |
|  | Jessica Van Meter, DNP |
| Response to Reviewers: | August 20th, 2025  Dear PLOS ONE Academic Editor and Reviewers,  We are grateful for the time and thoughtful feedback provided during the review of our manuscript. We have carefully revised the text to address all comments and believe these revisions have strengthened the clarity, rigor, and contribution of our work. Below we provide a point-by-point response, with reviewer comments in italics followed by our responses regarding the following manuscript:  Pediatric Capacity Building in Northern Laos: An Evaluation of the Pediatric Nursing Training Program at Lao Friends Hospital for Children |

|  | Methods   1. Study setting and design   Comment: Please include a description of the LFHC setting (level of hospital, services rendered, staff strength, staff caliber, and possibly child mortality figures). Response: We have added a description of LFHC as a tertiary pediatric referral hospital in Luang Prabang, Laos. The revised text now includes information on the types of services provided, the scope of staff roles, and its role in serving a vulnerable pediatric population.   2. Sample selection and recruitment   Comment: Certain sentences belong in other sections (voluntary participation → ethics, pilot test → validity, interview details → data collection).  Response: We have reorganized these elements into appropriate sections. Voluntary participation and confidentiality are now described under Ethical Considerations. Details of the pilot survey are presented under Tools, and interview procedures are included in the Data Collection subsection.   3. Data analysis   Comment: More detail is needed on quantitative (means, SDs) and qualitative (grounded theory, coding, theme identification, anonymization) analysis.  Response: We have expanded our description of both quantitative and qualitative data analysis. For the quantitative data, we clarify that descriptive statistics (means, SDs, and percentages) were calculated using Microsoft Excel. For qualitative data, we elaborate on the grounded theory approach, including the coding process, identification of themes, triangulation by multiple reviewers, and anonymization of transcripts.  Ethical Considerations  Comment: Clarify “non-research” IRB status, type of approval from LFHC, and include details on participation, right to withdraw, risks/benefits, compensation, confidentiality, and data protection.  Response: We have clarified that the Vanderbilt University IRB reviewed this project and granted it “exempt” status, which is a formal university designation for program evaluations that do not involve biomedical interventions or place participants at risk. This status indicates that the project does not require ongoing IRB monitoring but is nonetheless subject to university oversight and human subjects’ protection standards. In addition, the LFHC Research Committee independently reviewed and approved the study protocol. The revised section now explicitly outlines voluntary participation, right to withdraw, absence of financial incentives, anonymity, confidentiality safeguards, and secure storage of recordings.  Results   1. Likert scale clarification   Comment: Clarify if Likert ratings were by direct observation, assessment, or recall. Response: We now specify that Likert ratings were completed by expatriate clinical mentors based on their direct observation of nurses’ skills during training and practice, with some recall bias acknowledged.   2. Mean score changes (Table 2)   Comment: Explain improvements and declines in specific skills; discuss significance of change from 3.5 → 4.4.  Response: We have expanded the Results and Discussion sections to reflect that improvements in mean scores demonstrate not only increased clinical skills but also enhanced critical thinking among Lao nurses. We also added a discussion of the significant increase in mentors’ self-reported knowledge of the Lao nursing role (3.5 → 4.4).   3. Potential biases   Comment: Expatriate mentors may have biased program ratings.  Response: We acknowledge this limitation explicitly in the Discussion, noting that expatriate mentors both delivered and evaluated the program, which may have influenced objectivity. To mitigate this, we used multiple data sources (surveys, interviews, curriculum review) to triangulate findings.   4. Self-reflection section   Comment: Provide graphical presentation, analyze influence of changes, link to skills, discuss social desirability bias.  Response: We appreciate this suggestion. While we considered adding a graphical presentation, we determined that the small sample size and exploratory nature of the data limited the utility of additional figures. Instead, we have clarified in the manuscript |
| --- | --- |

|  | that the changes in self-reflection responses most likely reflect an increased cultural understanding of Lao nursing roles, though these findings cannot be directly linked to skill-based outcomes presented in Table 2. We therefore avoid overinterpretation of the results. To address the reviewer’s concern about social desirability bias, we have expanded the Discussion to note this as a potential limitation and outlined the measures taken to reduce its impact, including the use of anonymized surveys, voluntary participation, and assurances of confidentiality.  5. Semi-structured interviews  Comment: Present findings in qualitative structure with quotes, not narration. Response: The section has been significantly revised to highlight key themes supported by participant quotes, presenting findings in a qualitative structure to better reflect interview data.  Discussion   1. Key findings   Comment: Expand discussion of cultural immersion and link to transcultural nursing theory.  Response: We added content linking cultural immersion of mentors to the principles of transcultural nursing theory, emphasizing how cultural humility was integral to program success.   2. Significant difference in mean scores (3.5 vs. 4.4) Comment: Discuss this difference.   Response: Addressed as noted above, highlighting the significance of this increase in enhancing mentors’ understanding of the Lao nursing role.   3. Limitations and biases   Comment: Address recall bias, expatriate role, technology issues, lack of Lao staff voice, etc.  Response: These have all been integrated into the expanded Limitations section.   4. Further research   Comment: Suggest areas for future study.  Response: A new subsection recommends further research on culturally relevant, sustainable training models in resource-variable settings, with explicit inclusion of cultural humility in cross-cultural partnerships.  We sincerely thank the reviewers and editor for their valuable comments, which have significantly improved the quality of our manuscript. We hope the revisions and clarifications provided meet expectations and respectfully resubmit our manuscript for your consideration.  Sincerely,  Rebecca Silvers DNP, APN, CPNP-AC, CCRN, RNFA On behalf of all co-authors |
| --- | --- |
| Additional Information: |  |
| Question | Response |
| Financial Disclosure  Enter a financial disclosure statement that describes the sources of funding for the work included in this submission. Review the [submission guidelines](http://journals.plos.org/plosone/s/submission-guidelines#loc-financial-disclosure-statement) for detailed requirements. View published research articles from [PLOS ONE](https://journals.plos.org/plosone/) for specific examples.  This statement is required for submission and will appear in the published article if the submission is accepted. Please make sure it is accurate. | The author(s) received no specific funding for this work. |

| Funded studies  Enter a statement with the following details:   - Initials of the authors who received each award - Grant numbers awarded to each author - The full name of each funder - URL of each funder website - Did the sponsors or funders play any role in the study design, data collection and analysis, decision to publish, or preparation of the manuscript? |  |
| --- | --- |
| Did you receive funding for this work? |  |
| Competing Interests  Use the instructions below to enter a competing interest statement for this submission. On behalf of all authors, disclose any [competing interests](http://journals.plos.org/plosone/s/competing-interests) that could be perceived to bias this work—acknowledging all financial support and any other relevant financial or nonfinancial competing interests.  This statement is required for submission and will appear in the published article if the submission is accepted. Please make sure it is accurate and that any funding sources listed in your Funding Information later in the submission form are also declared in your Financial Disclosure statement.  View published research articles from [PLOS ONE](http://journals.plos.org/plosone/) for specific examples. | The authors have declared that no competing interests exist. |

| NO authors have competing interests  Enter: The authors have declared that no competing interests exist.  Authors with competing interests  Enter competing interest details beginning with this statement:  I have read the journal's policy and the authors of this manuscript have the following competing interests: [insert competing interests here] |  |
| --- | --- |
| * typeset |  |
| Ethics Statement  Enter an ethics statement for this submission. This statement is required if the study involved:   - Human participants - Human specimens or tissue - Vertebrate animals or cephalopods - Vertebrate embryos or tissues - Field research   Write "N/A" if the submission does not require an ethics statement.  General guidance is provided below. Consult the [submission guidelines](http://journals.plos.org/plosone/s/submission-guidelines#loc-guidelines-for-specific-study-types) for detailed instructions. Make sure that all information entered here is included in the Methods section of the manuscript. | This study received ethical approval from the Institutional Review Board (IRB) at Vanderbilt University under exempt (non-research) status (Protocol #202298).  Additional approval was provided by the Lao Friends Hospital for Children’s Research Committee (See letter from Matt Evans, Nursing Director, approving the project). Participation in the study was voluntary, and participants were informed that consent would be implied through their completion of the survey and interviews. |

| Format for specific study types  Human Subject Research (involving human participants and/or tissue)   - Give the name of the institutional review board or ethics committee that approved the study - Include the approval number and/or a statement indicating approval of this research - Indicate the form of consent obtained (written/oral) or the reason that consent was not obtained (e.g. the data were analyzed anonymously)   Animal Research (involving vertebrate animals, embryos or tissues)   - Provide the name of the Institutional Animal Care and Use Committee (IACUC) or other relevant ethics board that reviewed the study protocol, and indicate whether they approved this research or granted a formal waiver of ethical approval - Include an approval number if one was obtained - If the study involved non-human primates, add additional details about animal welfare and steps taken to ameliorate suffering - If anesthesia, euthanasia, or any kind of animal sacrifice is part of the study, include briefly which substances and/or methods were applied   Field Research  Include the following details if this study involves the collection of plant, animal, or other materials from a natural setting:   - Field permit number - Name of the institution or relevant body that granted permission |  |
| --- | --- |
|  |  |
| Data Availability  Authors are required to make all data underlying the findings described fully available, without restriction, and from the time of publication. PLOS allows rare exceptions to address legal and ethical concerns. See the [PLOS Data Policy](http://journals.plos.org/plosone/s/data-availability) and [FAQ](http://journals.plos.org/plosone/s/data-availability#loc-faqs-for-data-policy) for detailed information. | Yes - all data are fully available without restriction |

| A Data Availability Statement describing where the data can be found is required at submission. Your answers to this question constitute the Data Availability Statement and will be published in the article, if accepted. |  |
| --- | --- |
| Important: Stating ‘data available on request from the author’ is not sufficient. If your data are only available upon request, select ‘No’ for the first question and explain your exceptional situation in the text box. |  |
| Do the authors confirm that all data underlying the findings described in their manuscript are fully available without restriction? |  |
| Describe where the data may be found in full sentences. If you are copying our sample text, replace any instances of XXX with the appropriate details.   - If the data are held or will be held in a public repository, include URLs, accession numbers or DOIs. If this information will only be available after acceptance, indicate this by ticking the box below. For example: All XXX files   are available from the XXX database (accession number(s) XXX, XXX.).   - If the data are all contained within the manuscript and/or Supporting Information files, enter the following: All relevant data are within the manuscript and its Supporting Information files. - If neither of these applies but you are able to provide details of access elsewhere, with or without limitations, please do so. For example:   Data cannot be shared publicly because of [XXX]. Data are available from the XXX Institutional Data Access / Ethics Committee (contact via XXX) for researchers who meet the criteria for access to confidential data.  The data underlying the results presented in the study are available from (include the name of the third party | All relevant data are within the manuscript and its Supporting Information files. |

| and contact information or URL).  • This text is appropriate if the data are owned by a third party and authors do not have permission to share the data.  * typeset |  |
| --- | --- |
| Additional data availability information: |  |

Manuscript	[Click here to access/download;Manuscript;Manuscript.docx](https://www2.cloud.editorialmanager.com/pone/download.aspx?id=41679351&guid=69e354c8-1e7e-4dff-8168-cf6cb8369e74&scheme=1)

1

2

3

4

5

6

7

8

9 Pediatric Capacity Building in Northern Laos: An Evaluation of the Pediatric
10 Nursing Training Program at Lao Friends Hospital for Children

11

12

13

14

15

16	**Short title**: Pediatric Nursing Capacity Building in Laos

17

18 **Authors**:
19 Rebecca SILVERS DNP, APN, CPNP-AC, CCRN, RNFA^[1]^*;
20 Maly CHITTAPHAI RN^[2]^;
21 Matt EVANS, MS, MPH, RN ^2^;
22 Maddie WONG, MS^1^;
23 Jessica VAN METER, DNP, RN, APN-BC, CCRN, A-EMT^[3]^ 24
25 **Maly Chittaphai:** Conceptualization (support), resources (support), review, and editing (equal).
26 **Matt Evans**: Conceptualization (support), resources (lead), review, and editing (equal). **Rebecca**
27 **Silvers**: Conceptualization (lead); Methodology (lead), writing – original draft (lead); formal
28 analysis (lead); writing – review and editing (equal). **Jessica Van Meter**: Conceptualization
29 (supporting); Writing – original draft (supporting); Writing – review and editing (equal). **Maddie** 30	**Wong**: Writing – review and editing (equal)

31

32	**Conflict of Interest**: The authors deny any conflicts of interest.

33

34 ***Corresponding Author:**
35 Rebecca.silvers@ucsf.edu (RS)

36

37

38

39

40

# ABSTRACT

**Background**: In 2015, the child mortality rate in Laos was 53.6 per 1,000 live births. The Lao Friends Hospital for Children (LFHC) in Luang Prabang was established to enhance access to specialized pediatric care. To support workforce development, a Pediatric Nursing Training Program was implemented. However, the program has not undergone a formal evaluation since its inception. This study aims to evaluate the program’s first five years.

**Study Design**: The evaluation included a review of curriculum documents via an online platform and feedback collection through an online survey administered to former expatriate nurse volunteers who provided clinical mentorship. Additionally, semi-structured interviews were conducted with former nurse leaders who designed and implemented the program. Qualitative data were analyzed thematically and validated through triangulation.

**Results**: The curriculum review revealed system-based and course-specific plans, including clear objectives, teaching methods, and evaluation tools. Of the 148 clinical mentors surveyed, a 45% response rate was achieved. Survey responses indicated improvements in nurses’ clinical skills since the program’s inception, with mentors emphasizing the importance of cultural attunement to Lao staff. Six out of seven former nurse leaders participated in interviews. Key themes emerged, highlighting the value of culturally relevant educational materials, multimodal teaching strategies, a train-the-trainer approach, and clinical mentorship as integral components of the program.

**Conclusion**: The development of pediatric nursing specialization at LFHC was facilitated by structured curriculum plans, cultural sensitivity, diverse teaching methodologies, and robust clinical mentorship. These findings provide valuable insights for designing culturally competent and sustainable training programs in Laos and similar contexts.

**Keywords**: knowledge to practice gap, pediatric acute care, nurse training program, clinical mentorship, cultural humility

## 1. Introduction

Child mortality worldwide has reduced dramatically over the last few decades. In 1990, one in 11 children died before reaching age five, compared to one in 26 in 2017 [1]. Although the global rate of child mortality is on a decline, there remains a great deal of variation in death rates across geographical locations. Ninety-five percent of pediatric deaths occur in low- and middle-income countries [2]. In 2017, the number of under-5 deaths was 5.4 million globally; half of those deaths were children in sub-Saharan Africa, and another 30% were children in Southern Asia [1].

Access to appropriate pediatric care is the largest determinant of child morbidity and mortality, and therefore, understanding access to care is essential to uncovering solutions worldwide [3]. For a country or region to provide access to pediatric health services, a system must optimize the number of clinicians and their clinical expertise, environmental and physical resources, and address patient variables and barriers to care. In addition, clinicians must be prepared with the clinical skills and knowledge to make appropriate clinical judgments in pediatric care [4].

There are numerous challenges in low- and middle-income countries (LMICs) to optimizing the nursing workforce to provide access to pediatric care. Additionally, high-quality professional nursing education in LMICs faces many challenges, including a lack of available faculty, limited clinical preceptorship hours, and failure of available evidence for best practice to apply to their clinical settings [5,6]. As a result, nurses struggle to apply what they have learned with available resources and standards of practice in their local setting, which results in a lack of standardized care and poor confidence in nursing practice [7]. Thirdly, the opportunities for specialized pediatric training are limited [8].

## 2. Background

Laos, a landlocked country in Southeast Asia, is characterized by mountainous terrain and significant ethnic, religious, and linguistic diversity. Despite its rich cultural heritage, Laos faces considerable economic challenges and is classified by the World Bank as a low-income country. Its healthcare infrastructure is still developing, particularly in rural areas outside the capital city. To bridge gaps in healthcare delivery, various organizations in Laos partner with international aid agencies and non-governmental organizations (NGOs) to strengthen healthcare programs and enhance the education of medical professionals [9].

Efforts to improve healthcare in low- and middle-income countries (LMICs) like Laos have increasingly focused on supplementary education and training. Academic institutions and NGOs play a pivotal role in elevating clinical practice standards by providing specialized training for healthcare workers [10-13]. These initiatives aim to address critical shortages in healthcare workforce expertise and improve health outcomes in resource-limited settings.

In 2014, the non-governmental organization Friends Without Borders (FWAB), in collaboration with the Ministry of Health, launched an initiative to expand access to pediatric specialty care in northern Laos. This effort led to the establishment of a pediatric specialty medical center aimed at addressing critical healthcare gaps and reducing the child mortality rate, which was 53.6 per 1,000 live births. A key focus of the initiative was capacity building through comprehensive training programs for Lao healthcare professionals—including doctors, nurses, allied health workers, and administrative staff—to strengthen local expertise and improve health outcomes.

The Lao Friends Hospital for Children (LFHC) opened its doors in January 2015, alongside the launch of the Pediatric Nursing Training Program. This program was designed to enhance the clinical competencies of nursing staff through culturally tailored didactic education and bedside clinical mentorship. The training program is supported by expatriate nurse leaders who commit to one- to two-year terms, as well as short-term nursing volunteers who provide bedside mentorship during their one- to twelve-month placements. Didactic sessions take place in the hospital’s designated learning spaces, with protected time allocated for nurses to participate in educational activities.

The pediatric nursing training program at LFHC is the first of its kind in Laos, yet a program evaluation has not been conducted. Thus, the purpose of this study was to evaluate the first five years of the specialized nursing training program. Aims including evaluating whether the program implemented culturally relevant training and increased nurses’ clinical knowledge and their ability to apply their knowledge within the clinical setting.

## 3. Methods

### 3.1 Study Setting & Design

This study was conducted at the Lao Friends Hospital for Children (LFHC), a no-cost tertiary pediatric referral facility located in Luang Prabang, northern Laos. According to

UNICEF estimates, the pediatric population in Laos is approximately 2.8 million; however,

LFHC is one of only two dedicated pediatric hospitals in the country. Since opening its doors in 2015 with an outpatient clinic and inpatient wards, LFHC has expanded to provide a full range of specialized services, including surgery, neonatal, emergency, and critical care, a developmental clinic, and a thalassemia program. Annually, the hospital now serves more than 45,000 children from across northern Laos and has provided access to care for over 200,000 patients in the first ten years of operations. LFHC continues to train a growing workforce of Lao physicians and nurses, many of whom are new to pediatric specialty care.

The evaluation of the Pediatric Nursing Training Program at LFHC involved a multimethod approach, including a review of the curriculum and associated documents, electronic surveys of expatriate volunteer mentors, and interviews with former nurse leaders from LFHC. Due to travel restrictions imposed during the COVID-19 pandemic, all components of the evaluation were conducted remotely using online platforms. Surveys were designed and distributed via REDCap, a secure web-based platform for managing online surveys and databases [14, 15]. The survey was open for participation from December 15, 2020, to February 1, 2021. In-depth qualitative interviews with former nurse leaders were conducted via Zoom between February and March 2021. Additionally, the review of curriculum documents was carried out concurrently using a shared Office Drive hosted on a secure cloud-based platform.

### 3.2 Theoretical Framework: Transcultural Theory of Nursing

Madeleine Leininger's Transcultural Theory of Nursing has been used to support the understanding of diverse populations to provide culturally competent care [16]. It has been successfully utilized to implement sustainable health promotion activities in resource-variable settings [17]. The success of any sustainable healthcare development plan that includes bringing expatriate teams to assist in establishing new health systems and training the healthcare workforce is directly related to the consideration of cultural relevance and sensitivity.

### 3.3 Sample Selection and Recruitment

Participants were purposively selected based on their direct involvement in the LFHC Pediatric Nursing Training Program during the study period 2015-2019. Two groups were included which included former LFHC clinical mentors and nurse leaders.

Clinical mentors were international nurse volunteer who served as mentors provided didactic education, bedside clinical teaching, and supported Lao nurses in applying knowledge to practice. They also contributed to curriculum development and competency evaluation. Contact information for clinical mentors who voluhnteereed at LFHC between 2015 and 2019 was provided by the Human Resources Department and the nursing director at LFHC. An introductory email was sent to potential participants, explaining the project and inviting them to complete the online survey via a provided REDCap link.

Nurse leaders consisted of expatriate staff who worked at LFHC in a Nursing Director or Nursing Eductor role. They played a critical role in adapting and sustaining the program within the local context, while living in Lao, as well as supported curriculum design, mentorship structures, and leadership development. All nurse leaders were invited by the research team and current Nirsing Director to participate in semi-structured interviews via email with a request to choose an interview date and time, if they were interested in participation.

This sampling ensured representation from those who directly designed, implemented, and guided the program, capturing perspectives central to evaluating its outcomes and sustainability.

### 3.4 Data Tools and Collection

Data collection occurred simultaneously through a review of curriculum documents, a

REDCap survey, and through semi-structured interviews over a four-week period. The LFHC

Nurse Training Program curriculum and other relevant documents were gathered and reviewed to evaluate the following themes: program design, program objectives, training schedule, and the content of the program. Attention to the train-the-trainer model and cultural relevance was also noted.

The LFHC Clinical Mentor Survey consisted of three sections and was distributed in

January 2021 to all expatriate nurses who participated in the clinical mentorship program from

2015 to 2019. The first section gathered information about participants’ nursing background, clinical experience prior to LFHC, and any prior experience working abroad. The second section focused on the expatriate nurses’ perspectives regarding the clinical skill set of the Lao staff nurses during their time at LFHC. Ratings were done based on recall of past experiences of direct observation. The final section provided an opportunity for self-reflection on the expatriate nurses’ contributions to the training process, specifically in clinical mentoring and cultural competence. A pilot test was conducted to enhance the face and content validity of the survey before it was distributed. All the data collected from the survey were imported via REDCap into a Microsoft Excel spreadsheet for analysis.

Interviews with former nurse leaders were scheduled at a time convenient for each participant. The interviews were conducted via Zoom and were recorded with the participants' permission. Each interview was designed to last approximately one hour. The LFHC Program Evaluation Interview Guide was used for the semi-structured interviews and consisted of 14 questions (Fig. 1). Two questions collected demographic data about the nurse leaders, while the remaining 12 questions focused on evaluating the program. These questions encouraged participants to reflect on their role in facilitating the LFHC Nurse Training Program. To ensure content and face validity, the interview guide was tested by the research team at Vanderbilt University. The interviews were recorded by the interviewer and submitted to Trint Ltd., an online high-quality and secure transcription service, to be transcribed [18].

Fig 1. Interview Guide

### 3.5 Data Analysis

Data collected from the electronic REDCap survey were analyzed using descriptive statistics. Demographic variables were summarized as frequencies and percentages. Likert-scale responses were analyzed in Microsoft Excel using built-in functions (AVERAGE and STDEV) to calculate mean scores and standard deviations. For the qualitative component, interviews were audio-recorded, transcribed verbatim, and anonymized prior to analysis. Data were examined using thematic analysis informed by grounded theory methodology. Coding was performed manually by two members of the research team, followed by review with the Principal Investigator. The coding process involved open coding to identify initial concepts, axial coding to organize these into categories, and iterative refinement of emerging themes and subthemes. Discrepancies were resolved through consensus meetings, and an audit trail of coding decisions was maintained. Triangulation across coders and data sources enhanced credibility, and crossvalidation of findings strengthened the rigor and trustworthiness of the analysis.

### 3.6 Ethical Considerations

This study received ethical approval from the Institutional Review Board (IRB) at Vanderbilt University under exempt (non-research) status (Protocol #202298). Additional approval to proceed with the study was granted by the Lao Friends Hospital for Children (LFHC) Research Committee following review of the study protocol. Participation in the study was entirely voluntary. Consent was implied through the completion of surveys and participation in interviews or focus groups, with participants informed of their right to withdraw at any time

245 without penalty. No financial incentives or compensation were provided. All data were
246 anonymized to safeguard confidentiality, and results are reported in aggregate to protect 247	participant identity. Audio recordings of interviews and focus groups were obtained only with

248	consent and remain securely stored, with assurance of non-disclosure beyond the research team.

## 4. Results

### 4.1 Curriculum Documents Review

The curriculum documents were stored on a privately accessed Microsoft OneDrive, with access controlled by the head of information technology at LFHC. The OneDrive contains organized folders, including audits, competency, courses, curricula, English vocabulary list, exams, in-services, medication safety, emergency simulations, nursing orientation, students, and training records, each with several subfolders. A review of the curriculum documents revealed thorough and well-organized record-keeping of clinical courses taught as part of the education and training programs since 2016. The training documents and PowerPoint presentations for each course were easily accessible in their designated subfolders. Lesson plans clearly outline the targeted learning objectives for each course, along with the associated evaluation methods, which include hands-on activities and group exercises. The clinical competency evaluations were clearly documented for each nurse at LFHC, but the schedule for student reassessment or evaluation was unclear. It was also unclear if there was testing to measure expatriate nurses’ understanding of the content that they were teaching.

### 4.2 LFHC Clinical Mentorship Survey

The LFHC Clinical Mentorship Survey was distributed to 148 former nursing volunteers who served from 2015 to 2019. Sixty-seven of the volunteers responded and completed the survey in full for a return rate of 45%.

*4.2.1 Expatriate Nurse Demographics*

The first section of the survey focused on the demographics and work history of expatriate nursing volunteers (Fig. 2). A total of 67 survey responses were analyzed, revealing participation across all years of the Pediatric Nursing Training Program's initial operation. The majority of respondents volunteered in 2017 (n=19) and 2018 (n=17), with additional participation in 2015 (n=6), 2016 (n=10), and 2019 (n=15). Regarding the duration of service at LFHC, most respondents (64.2%) reported volunteering for two to six months (n=43). Seventeen volunteers (25.4%) stayed for one month, five (7.5%) for six months to one year, and two (3%) for more than one year.

The participants represented a diverse range of countries, with the majority identifying as Australian (n=21), American (n=19), British (n=11), Canadian (n=8), or New Zealander (n=3).

Additional countries of origin included Italy (n=2), Spain, Portugal, and Denmark (n=1 each).

Notably, 41 respondents (61%) had prior experience in resource-variable settings (RVS).	Forty-eight expatriate volunteers (71.5%) identified as bedside registered nurses, while others reported varied roles, including clinical nurse specialists (13.4%, n=9), nurse educators (11.9%, n=8), advanced practice nurses or nurse practitioners (7.5%, n=5), operating room nurses (4.5%, n=3), professors at university nursing schools (3%, n=2), and nurse anesthetists (1.5%, n=1).

Participants also had diverse clinical backgrounds. More than half (50.7%, n=34) had experience in pediatric wards. Other areas of clinical experience included emergency departments (38.8%, n=26), pediatric-specific emergency departments (27.3%, n=25), pediatric critical care (26.9%, n=18), pediatric clinics (20.9%, n=14), neonatal intensive care (17.9%, n=12), and pediatric hematology and oncology (16.4%, n=11).

The respondents varied in their years of clinical nursing experience before joining LFHC: 17 (25.4%) had one to five years of experience, 22 (32.8%) had five to ten years, nine (13.4%) had 10 to 15 years, and 19 (28.4%) had over 15 years of experience.

**Table 1. Demographics of Participants: LFHC Clinical Mentorship Survey.**

| *Demographic Data (N=67)* |  |
| --- | --- |
| **Start participating at LFHC (Year)** | **n (%)** |
| 2015 | 6 (9) |
| 2016 | 10 (14.9) |
| 2017 | 19 (28.4) |
| 2018 | 17 (25.4) |
| 2019 | 15 (22.4) |
| **Length of time at LFHC** |  |
| <1 month | 17 (25.4) |
| 2-6 months | 43 (64.2) |
| 6 months – 1 year | 5 (7.5) |
| >1 year | 2 (3) |
| **Age (years)** |  |
| 21-30 | 32 (47.8) |
| 31-39 | 14 (20.9) |
| 40-49 | 5 (7.5) |
| 50-59 | 11 (16.4) |
| >59 | 5 (7.5) |
| **Home Country** |  |
| Australia | 21 (31.3) |
| United States | 19 (28.4) |
| United Kingdom | 11 (16.4) |
| Canada | 8 (11.9) |
| New Zealand | 3 (4.5) |
| Other ^a^ | 5 (7.5) |
| **Profession ^b^** |  |
| Bedside Nurse | 48 (71.6) |
| Clinical Nurse Specialist | 9 (13.4) |
| Nurse Educator | 8 (11.9) |
| Advance Practice Nurse (Nurse Practitioner) | 5 (7.5) |
| Operating Room/Theater Nurse | 3 (4.5) |
| Professor/Teacher at School of Nursing | 2 (3) |
| Nurse Anesthetist | 1 (1.5) |
| Other ^c^ | 6 (9) |
| **Clinical Experience (years)** |  |
| 1 - 5 | 17 (25.4) |
| 5- 10 | 22 (32.8) |
| 10-15 | 9 (13.4) |
| >15 | 19 (28.4) |
| **Previous experience in resource-limited setting** |  |
| Yes | 41 (61.2) |
| No | 26 (38.8) |
| **# countries experience working** |  |
| None | 26 (38.8) |
| 1-3 | 30 (44.8) |

| 3-10 | 9 (13.4) |
| --- | --- |
| >10 | 2 (3) |
| **Clinical experience ^a^** |  |
| Pediatric Ward | 34 (50.7) |
| Emergency department (A & E) | 26 (38.8) |
| Pediatric specific ED (A & E) | 25 (37.3) |
| Pediatric Critical Care (PICU) | 18 (26.9) |
| Pediatric Clinic | 14 (20.9) |
| Neonatal Intensive Care | 12 (17.9) |
| Pediatric Hematology & Oncology | 11 (16.4) |
| Other *** | 20 (29.9) |

^a^ Other includes Italy (n=2), Spain, Portugal, & Denmark ^b^ Participants were asked to mark all answers that apply ^c^ Other includes physician assistant, nurse manager (NICU), clinical nurse consultant (n=2), research ^d^ Other includes stroke triage, PACU (pediatric recovery room) (n=2), remote First Nations Primary Care Health Centre, Trauma, adult ICU (n=3), adult medical & surgical wards (n=7), school nurse in a public school, acute rehabilitation (n=3), hospice, Operating Theatre (n=3), Labor and Delivery (Maternity) (n=2)

*4.2.2 Evaluation of the Content of the Training Program*

In the second section of the LFHC Clinical Mentorship Survey, participants rated the Lao nursing staff on 14 skills using a Likert scale ranging from one to five where one referred to insufficient knowledge and being unable to complete the skill, while five referred to completing competence in knowledge and skill and being able to help support others (Table 2). The mean (standard deviation) of the clinical skill set rated by the participants in 2015 was 2.8 (0.4). Over the course of five years, the mean increased to 3.5 (0.5) in 2019. The greatest changes in mean scores between 2015 and 2019 were starting an IV live (1.1), recognition of a patient having a seizure (1), application of weight-based medication dosing (0.8), and application of appropriate respiratory support for hypoxia (0.8). The smallest change in mean scores was seen in the knowledge of normal vital signs for age (0.2), performing a 12-lead ECG (0.3), and recognition of a patient in respiratory distress or failure (0.3).

## 317 Table 2. LFHC Clinical Mentorship Survey: Nursing Clinical Skill Data by Year

| **Nursing Clinical Skills** | **2015** | **2016** | **2017** | **2018** | **2019** |
| --- | --- | --- | --- | --- | --- |
| Taking vital signs on a pediatric patient | 3.3 | 3.7 | 3.6 | 3.6 | 3.8 |
| Knowledge of normal vital signs patients of various ages | 3 | 2.9 | 3.1 | 3.4 | 3.2 |
| Drawing blood through a venipuncture | 3.7 | 4 | 4 | 4.3 | 4.4 |
| Starting an intravenous (IV) line to administer intravenous fluids/medications | 3 | 4.1 | 3.9 | 4.1 | 4.1 |
| Using a stethoscope to perform a respiratory examination | 2.5 | 3.1 | 2.7 | 2.9 | 3.2 |
| Application of weight-based medication dosing | 2.7 | 2.8 | 2.7 | 3.1 | 3.5 |
| Preparing and administering oral medications | 3.2 | 3.7 | 3.5 | 3.6 | 3.9 |
| Preparing and administering IV administration | 2.8 | 3.5 | 3.3 | 3.4 | 3.5 |
| Performing an electrocardiogram (EKG or ECG) | 2.5 | 2.7 | 2.7 | 2.8 | 2.8 |
| Recognition of hemodynamically unstable patient | 2.3 | 2.6 | 2.5 | 2.8 | 2.8 |
| Recognition of a patient in respiratory distress or failure | 2.8 | 3.1 | 2.9 | 3.2 | 3.1 |
| Application of the appropriate oxygenation delivery devise for hypoxemia/respiratory distress | 2.7 | 2.8 | 2.9 | 3.2 | 3.5 |
| Recognition of a child with seizures | 2.3 | 3.1 | 3.1 | 3.2 | 3.3 |
| Participation in pediatric or neonatal cardiopulmonary resuscitation (CPR) | 2.7 | 2.9 | 2.8 | 3.1 | 3.3 |
| **Mean** | **2.8** | **3.2** | **3.1** | **3.3** | **3.5** |
| **Standard Deviation** | 0.4 | 0.5 | 0.5 | 0.5 | 0.5 |

*4.2.3 Participants’ Self-Reflection on Contributions to the Training Program*

The third section of the survey focused on the participants' self-reflection on their contribution to the training process pertaining to clinical mentoring and cultural competence. The nurses responded to 14 statements on a Likert scale of one to five, where one was “strongly disagreed” and five was “strongly agree.” Eighty-four percent (n=56) of participants agreed or strongly agreed that their knowledge of Lao culture contributed to their ability to provide clinical mentorship and education to the LFHC nursing staff at the bedside. The majority also agreed or strongly agreed (90%, n=60) that participating in cultural events in the hospital and town increased their understanding go Lao values and beliefs. Additionally, 96% (n=64) of volunteers responded that they agree or strongly agree that as their time at LFHC progressed, they could recognize barriers to learning in Lao and adapt their teaching methods.

Sixty-one percent (n=41) of participants strongly agreed they were able to facilitate more constructive clinical mentorship as the strength of their relationship with the Lao staff strengthened (mean 4.5, SD 0.8). The participants agreed that they recognized the importance of cultural competence in the nursing training program (mean 4.6, SD 0.5). 96% (n=64) of the participants strongly agreed or agreed that they understood the role of nursing in Laos at the end of their time in LFHC (mean 4.4, SD 0.6). Fifty-five percent of participants agreed or strongly disagreed with understanding the role of nursing in Laos prior to volunteering at LFHC (mean

3.5, SD 1.0).

There was neither an agreement or disagreement (mean 3.0, SD 1.0) that the Lao nurses struggled to apply their classroom education to the clinical setting, but 93% (n=62) of the participants agreed or strongly agreed that they felt comfortable recognizing the knowledge to practice gap and addressing it as they mentored Lao staff at the bedside (mean 3.9, SD 0.6). Overall, the clinical mentors felt that their time working with the Lao nurses contributed to an increase in the clinical skills of the Lao staff (mean 3.9, SD 0.7).

### 4.3 LFHC Program Evaluation: Semi-Structured Interviews

*4.3.1 Demographics*

During the first two years of the program at LFHC, a nursing director managed both the administrative and educational programs for the nurses, and in the latter three years, there was an additional role of a nurse educator (Participant 1). Semi-structured interviews were conducted with six former nurse leaders at LFHC. Two of the nurse leaders were from Australia; one was from the United Kingdom, one was from New Zealand, one was from Canada, and one was from the United States. The amount of time each nurse leader worked at LFCH ranged from nine months to two years. Based on the content analysis of the interview transcripts, many themes (italicized below) emerged related to the following topics: nursing curriculum development, clinical mentorship program, and cultural competence.

*4.3.2 Nursing Curriculum Development*

*Continual Revision of Curriculum*

Participants described the evolution of the nursing curriculum as a dynamic and responsive process. In the early years of hospital development, training began with a brief WHO course, which several participants felt was too limited. One explained, “The initial training was only a short course based on the WHO’s little blue book” (Participant 2). Over time, the curriculum expanded with input from global sources. As another noted, “We adapted from WHO and MSF system-based curricula, but always tried to fit them to our context” (Participant 3).	The onboarding of a formal nurse educator in 2016 marked a turning point. According to one participant, “With a dedicated educator, we could customize the curriculum based on the Lao nurses’ skill set and what we had learned in the first two years” (Participant 1). This shift included the deliberate removal of irrelevant material. “We eliminated content written for highincome countries that did not make sense here” (Participant 4). Curriculum revision became a regular process, with each new educator reassessing practice and clinical goals. “Every one or two years, when a new educator joined, they looked at what the staff needed and adjusted the content” (Participant 1). Lao nurse educators also played a critical role once they began teaching independently. One participant highlighted, “They were encouraged to change the curriculum themselves, based on whether it met the needs of their colleagues” (Participant 5).

*Adaptive Teaching Style Grounded in Humility*

Alongside curriculum changes, participants emphasized the importance of adapting teaching styles to meet the learning preferences of Lao nurses. Early experiences revealed mismatches between Western teaching expectations and local educational norms. “The nurses were used to memorizing and repeating answers; they had never been asked to question or participate before” (Participant 5). To address this, educators experimented with different methods. One described, “We used a trial and error system to find what worked. Interactive games ended up being very effective” (Participant 1).

Tone and demeanor were also crucial. As one educator explained, “The nurses were very

sensitive to the teacher’s tone and frustrations. They responded best to a compassionate, understanding style” (Participant 1). Participants consistently described the importance of humility in teaching, recognizing that respectful and culturally sensitive interactions were key to sustaining engagement.

*Creating a Culture of Critical Thinking*

A consistent theme across interviews was the need to move beyond rote memorization and cultivate critical thinking. Participants spoke about historical barriers. “Nursing education in Laos had been focused on memorization and tasks, not questioning or inquiry” (Participant 4). This approach left gaps in clinical reasoning. “The nurses were task compliant, but they did not always understand how interventions impacted the patient’s health” (Participant 3).

Several leaders provided striking examples. One recalled, “There were fundamental knowledge gaps, such as not connecting the pulse with the beating of the heart” (Participant 1). For the nurse leaders, teaching critical thinking was not only about knowledge but about transforming practice.

As one summarized, “Our goal was to build clinical inquiry, so they could trust their assessments and contribute to planning care with the team” (Participant 4).

*Nursing Empowerment and Sustainability*

Another key focus was empowering Lao nurses to assume leadership roles as part of

LFHC’s long-term sustainability plan. Early on, nurses struggled with confidence. “At first, they did not want to speak up or challenge norms; they stayed quiet even when they had good ideas” (Participant 3). Empowerment strategies included encouraging open communication. “We asked them to voice concerns and suggest improvements in patient care” (Participant 1).

A structured train-the-trainer model supported this goal. One leader explained, “We identified future nurse leaders by watching them over two years, looking at their clinical skills and emerging leadership” (Participant 1). Despite early barriers, including reluctance to stand out, progress was evident. “When one Lao nurse became an educational leader, it changed everything. It inspired others to step forward and use culturally appropriate teaching methods” (Participant 5).

*4.3.3 Clinical Mentorship Program*

*Evolving Roles of Clinical Mentors*

Participants described how the role of clinical mentors adapted as Lao nurses gained competence. Early on, mentors were embedded directly in patient care to provide safety oversight and hands-on training. “At first, the mentors worked side by side with the nurses in every unit, making sure they were safe and guiding them step by step” (Participant 4). As local nurses advanced, mentors shifted their focus. “By late 2017, we had Lao shift leaders, and mentors were assigned to individuals identified as needing extra support” (Participant 4). Mentors rotated through inpatient, neonatal, operating theater, and emergency units, mirroring the schedules of Lao staff to maximize learning opportunities.

*Selecting and Preparing Mentors*

Nurse leaders highlighted that mentor selection was critical to program success. Volunteers were initially recruited through academic institutions in the United Kingdom, including the Liverpool School of Tropical Medicine (LSTM) offers a Diploma in Tropical

Nursing for nurses wanting to work in LMICs. As one participant explained, “Many of our mentors came through the LSTM which provided them a good baseline knowledge for work with nurses here outside their home country” (Participant 2). Word of mouth also played a role. “A lot of our mentors were referrals from former volunteers” (Participant 3). Beyond clinical expertise, cultural humility emerged as an essential trait. “We looked for open-mindedness and adaptability, those who could adjust to Laos. Intensive care nurses often struggled here, but ER nurses thrived” (Participant 4).

*Supporting the Mentors through Cultural Humility*

Participants underscored the importance of preparing mentors to engage with humility and reflection. Preparation began with cultural orientation. “They received a pre-departure packet with Lao culture, their role, and details about LFHC” (Participant 4). On arrival, mentors shadowed seasoned volunteers and often spent time in villages to understand daily life. “They even had language learning time built into the schedule” (Participant 3).

Support also emphasized reflective practice. “We reminded mentors that they first needed to observe and learn the culture and workflow before suggesting changes” (Participant 3). Nurse leaders encouraged humility when navigating differences. “We told them, you are here to listen, to support, not to impose your way” (Participant 2). This reflective stance was reinforced through ongoing check-ins, debriefs, and monthly gatherings where mentors could process challenges and share insights.

*Staff Responses to Mentorship*

Initially, Lao staff were apprehensive. “At the bedside, they worried that having a mentor meant they were failing or being singled out” (Participant 1). Over time, perceptions shifted as mentors demonstrated respect and humility. “After explanations, they began requesting to be scheduled with mentors” (Participant 4). Trust deepened through personal relationships. “They invited mentors to weddings and dinners. They enjoyed those friendships” (Participant 4). Cultural humility shaped how successful mentors interacted with staff. “The nurses were very sensitive to tone and attitude. Volunteers who were patient and humble were much more successful” (Participant 3). One participant added, “In Lao culture, you do not raise your voice. The mentors who adapted to that had better results” (Participant 4). Language barriers also underscored the need for humility. “Medical terms do not always translate. The best mentors admitted when things were unclear and worked together to find simpler explanations” (Participant 6). Ultimately, humility built trust. “The most successful mentors created safe relationships where nurses felt comfortable saying what they did not understand” (Participant 6).

*4.3.4 Utilizing Cultural Humility within the LFHC Nursing Training Program*

*Respecting and Integrating Lao Cultural Practices*

All participants emphasized that cultural humility, defined as approaching local practices with openness and respect, was essential to developing the training program. As one nurse leader explained, “In Laos, post-birth customs require mothers to sit by the fire, and colostrum is believed to harm infants. We needed to understand these practices before we could teach neonatal care” (Participant 6). Nurses also navigated requests for traditional healing. “A family wanted to take their child home for a ceremony that they believed would cure pneumonia. We respected their wishes and provided antibiotics for their journey, orally, and intravenously when they returned” (Participant 1). End-of-life care further demonstrated the importance of humility. “Families often took children home to die, because of spiritual beliefs about spirits not returning.

It was important that we respected that, even if it conflicted with our own norms” (Participant 3).

*Adapting Global Curricula with Humility*

Participants reflected on the limitations of directly applying international curricula without considering local realities. “When volunteers came to teach WHO’s ETAT course, it did not work. The content was at the wrong level, language was a barrier, and some lessons clashed with local practices” (Participant 1). Over time, the team recognized the need for humility in training design. “We rewrote every course specifically for LFHC, taking into account culture, language, and the actual diseases here” (Participant 1). Continuous adaptation and openness to feedback from Lao nurses ensured that curricula remained relevant, effective, and grounded in local context.

*Practicing Humility as an Ongoing Process*

Participants emphasized that cultural humility was not a single adjustment but a continuous process of reflection and adaptation. One explained, *“Cultural humility meant reminding ourselves to keep learning. Every time we thought we understood, something new challenged our assumptions”* (Participant 2). Another reflected, *“The most important part was not assuming we knew better. We had to listen first, and then work together to find solutions”* (Participant 4). Humility was therefore seen as a mindset that required sustained openness to learning and adaptation in everyday interactions.

### 5. Discussion

5.1 Key Findings

The LFHC Nursing Training Program led to a notable improvement in the Lao nurses’ clinical skillset, as consistently emphasized by both clinical mentors and nurse leaders. In addition to strengthening procedural competence, the program facilitated a shift toward more advanced forms of clinical reasoning and problem-solving. Participants described how nurses began to move beyond rote memorization toward applying knowledge in practice, asking questions, and critically engaging with patient care. These changes reflect a transition from taskbased nursing to a more holistic and critical thinking approach to clinical practice.

A central factor in this development was the adaptation of the curriculum to the Lao context. By eliminating materials designed for high-income countries and customizing training to reflect local epidemiology, cultural practices, and available resources, the LFHC team ensured that the curriculum was both accessible and relevant. Such contextualization is consistent with literature on global health education, which emphasizes that externally imported curricula, without adaptation, often fail to achieve meaningful practice change [20–22]. The WHO ETAT course, for example, was attempted at LFHC but was reported to be of limited value without curriculum adaptations due to its misalignment with Lao nursing practice and cultural norms.

The program’s combined strategy of structured didactic sessions and hands-on clinical mentorship was another key contributor to success. Bedside mentorship created a feedback loop in which nurses could immediately apply knowledge, receive guidance, and reflect on practice. Nurse leaders noted that mentorship also facilitated psychosocial support, helping Lao nurses develop confidence and autonomy in their roles. Importantly, the clinical mentorship model was not static. Over time, it shifted from broad support of all staff to more targeted mentorship for junior nurses, allowing for efficient use of mentor expertise.

A further strength of the LFHC program was its investment in sustainability through leadership development. The modified train-the-trainer approach was pivotal in building longterm capacity. By the second year, a Lao nurse was identified as a future educator, and by 2019, this nurse was independently teaching the core curriculum. This not only ensured program continuity but also signaled a critical shift in power and ownership from expatriate staff to Lao leadership. In qualitative accounts, this transition was described as a turning point that inspired other nurses to aspire to leadership roles. Such findings align with global evidence that local leadership and peer-to-peer teaching are essential for enduring health system strengthening [19].

Perhaps most importantly, the LFHC experience demonstrated that cultivating critical thinking among nurses is possible even in traditionally rote-based educational systems. Through compassionate teaching, interactive learning strategies, and culturally sensitive mentorship, Lao nurses developed the ability to analyze clinical situations, anticipate complications, and contribute actively to care planning. This represents a substantial departure from historical models of nursing education in LMICs and highlights the potential for global nursing initiatives to drive pedagogical change.

Taken together, the findings of this evaluation underscore the importance of three interdependent elements in global nursing capacity building: contextual adaptation of curricula, integration of bedside mentorship, and investment in leadership through train-the-trainer strategies. The LFHC model demonstrates that when these elements are combined, nurse training can advance beyond technical skill acquisition to foster critical thinking, confidence, and sustainable leadership.

#### 5.2 Volunteer Perspectives and Bidirectional Learning

A significant finding from this study was the difference in the mean scores reflecting volunteers’ knowledge of the role of Lao nursing before and after participation at LFHC (3.5 vs. 4.4). This increase demonstrates that international clinical mentors not only contributed to the strengthening of Lao nurses’ clinical skills but also deepened their own understanding of the cultural, clinical, and leadership roles of local nurses. This highlights the reciprocal nature of the program, in which mentorship fostered bidirectional learning and exchange. Importantly, it underscores the value of cultural humility in cross-cultural partnerships, as volunteers gained greater awareness of the Lao context and the complexities of nursing practice in a resourcevariable setting. Such findings reinforce the importance of designing global mentorship initiatives as bidirectional learning opportunities that promote both local capacity building and the professional development of international collaborators.

#### 5.3 Implications for Global Nursing Education

The evaluation of the LFHC Nursing Training Program provides important lessons for nursing education in resource-variable settings. First, the findings reinforce that standardized, externally developed training programs may not achieve their intended outcomes without meaningful cultural and contextual adaptation. Efforts to implement global curricula such as WHO’s ETAT course highlight this limitation, as the program was perceived as misaligned with local needs and practices. In contrast, the LFHC model demonstrated that tailoring content to reflect local learning styles, language, and health system realities can lead to measurable gains in both clinical skills and critical thinking.

Second, the integration of bedside mentorship alongside didactic learning proved critical.

This aligns with growing evidence that experiential learning models are particularly effective for bridging knowledge-to-practice gaps in nursing. Mentorship not only improved technical competencies but also fostered professional identity and autonomy, qualities that are central to the advancement of nursing as a profession globally. For nurses in Laos, who traditionally trained in hierarchical and didactic systems, the opportunity to engage in dialogue, ask questions, and apply critical reasoning marked a transformative educational shift.

Finally, the study underscores the importance of embedding cultural humility into global nursing education. Expatriate mentors and educators at LFHC recognized that success depended on listening to Lao nurses’ perspectives, respecting local practices, and adapting pedagogical approaches accordingly. This emphasis on cultural humility rather than cultural competence promoted a spirit of mutual learning and helped establish trust, which was repeatedly highlighted in interviews as central to the program’s success.

#### 5.4 Sustainability and Leadership Development

A key contribution of the LFHC program was its explicit focus on sustainability. Too often, nursing education initiatives are limited by dependence on external trainers or short-term funding. In contrast, LFHC prioritized developing local leadership capacity through a modified train-the-trainer approach. By the second year of the program, a Lao nurse was formally identified as an educator, and within three years, they assumed independent responsibility for delivering the curriculum. This transition not only secured the program’s continuity but also elevated the professional role of nurses within the hospital.

Leadership development extended beyond the formal educator role. Qualitative findings revealed that exposure to mentorship and responsibility inspired many Lao nurses to envision themselves as leaders in their units. Several participants reflected that the program encouraged them to mentor junior colleagues, set standards for practice, and advocate for patient care improvements. These findings echo global nursing literature that emphasizes the link between local leadership and sustainable improvements in care quality.

The emphasis on sustainability also offers insights for broader health systems. By empowering Lao educators and fostering critical thinking among staff, the LFHC program contributed to resilience during crises, including the COVID-19 pandemic, when international travel restrictions severely limited expatriate support. In this way, the program illustrates how investments in LMIC nursing leadership can buffer against system shocks and promote longterm independence from external aid.

#### 5.5 Broader Global Health Implications

Beyond the context of LFHC, this evaluation contributes to the discourse on how global partnerships can advance health equity. It illustrates the value of bi-directional learning, where expatriate mentors adapted their teaching to local contexts while simultaneously learning from Lao nurses about culturally grounded care practices. Such partnerships advance not only technical skills but also relational capacity, which is critical for sustainable health system strengthening.

Furthermore, the LFHC experience provides a replicable model for other low- and middle-income countries seeking to strengthen pediatric nursing education. The combination of contextualized curricula, mentorship, and leadership development could be adapted for various clinical areas, from emergency and critical care to maternal and newborn health.

#### 5.6 Strengths and Limitations of the Study

The strengths of this project include strong engagement and participation from nursing leadership at LFHC. The clinical mentorship survey achieved a response rate of 45 percent, and six of the seven former nurse leaders from LFHC participated in semi-structured interviews, ensuring a broad representation of leadership perspectives. The use of a mixed-methods approach further strengthened the evaluation by helping to mitigate potential deficiencies and biases associated with reliance on a single method [23].

This study also has several limitations. A primary limitation was recall bias, as participants were asked to reflect on past work experiences [24]. Social desirability bias may have further influenced responses, particularly in relation to sensitive topics such as leadership and training effectiveness. The COVID-19 pandemic imposed additional challenges, including the absence of a standardized, validated survey tool, reliance on online technologies with varying levels of internet connectivity, and the inability to conduct in-person data collection. Lastly, due to travel restrictions during the pandemic, feedback from Lao staff could not be incorporated, limiting the inclusion of their perspectives, which are essential for a comprehensive evaluation of the LFHC Pediatric Nursing Training Program. Despite these limitations, the study provides critical insights into the development, implementation, and sustainability of pediatric nursing education and mentorship at LFHC, offering lessons that may be transferable to education program development in similar resource-variable settings.

#### 5.7 Recommendations for Further Research

This study provides evidence for new approaches to building and evaluating sustainable

and culturally relevant nursing training programs in resource-variable settings. The lessons learned from this project can inform future capacity-building initiatives by highlighting techniques to identify potential barriers early and equipping program developers with the tools necessary to ensure program success.

Future research is needed in several areas. First, further studies should evaluate the long-

term outcomes of train-the-trainer models, particularly regarding how they influence nurse retention, leadership development, and patient outcomes. Second, research should focus on refining methods for integrating cultural humility, which must be a central component of any cross-cultural partnership, into training curricula. This includes evaluating how cultural humility practices affect learner engagement, mutual respect, and the sustainability of acquired skills. Third, there is a need for comparative studies examining how different mentorship models, such as in-person, virtual, or hybrid, affect knowledge translation and practice change across resourcevariable contexts. Finally, involving nurses in LMIC’s perspectives, which were limited in this study due to COVID-19 restrictions, will be crucial for gaining a more comprehensive understanding of training needs, effectiveness, and sustainability.

### 6. Conclusion

The LFHC Nursing Training Program demonstrated that tailored, context-specific approaches can strengthen nursing skills, promote critical thinking, and foster leadership development in resource-variable settings. The use of bedside mentorship, didactic teaching, and a train-the-trainer model provided a sustainable pathway for building local capacity while advancing nursing leadership within LFHC. Importantly, the study underscores that cultural humility must be a central pillar in any cross-cultural training partnership. By approaching collaboration with openness, mutual respect, and recognition of everyone’s knowledge, training programs can achieve greater relevance, sustainability, and impact.

Future initiatives should not only focus on clinical skill-building but also prioritize the integration of cultural humility into program design and evaluation. Doing so will help ensure that global nursing partnerships remain equitable, contextually appropriate, and capable of driving lasting improvements in patient care and health system strengthening.

# Acknowledgements

The authors would like to express their sincere gratitude to the dedicated nursing staff at Lao Friends Hospital for Children (LFHC) for their unwavering commitment to skill development and their pioneering work in specialized pediatric care. We are deeply appreciative of our continued collaboration. We are also thank the former nursing leaders and volunteers who contributed to this evaluation, Their colletive contributions continue to strengthen nursing practice and leadership at LFHC, with Lao nursing stsff now taking the lead in fostering the

gowth of the furture of pediatric nursing in Laos.

**REFERENCES**

1. United Nations Children's Fund (UNICEF). Under five mortality [Internet]. 2018 [cited 2025

Jun 2]. Available from: https://data.unicef.org/topic/child-survival/under-five-mortality/

2. Liu L, Oza S, Hogan D, Perin J, Rudan I, Lawn J, et al. Global, regional, and national causes of child mortality in 2000–13, with projections to inform post-2015 priorities: An updated systematic analysis. Lancet. 2015;385(9966):430–40.
3. Rutherford M, Dockerty J, Jasseh M, Howie S, Herbison P, Jeffries D, et al. Access to health care and mortality of children under 5 years of age in the Gambia: A case-control study. Bull World Health Organ. 2009;87(3):216–25.
4. Kissoon N, Burns J. Who should get pediatric intensive care when not all can? A call for international guidelines on allocation of pediatric intensive care resources. Pediatr Crit Care Med. 2014;15(1):82–3.
5. World Health Organization. WHO global disability action plan 2014–2021: Better health for all people with disability [Internet]. Geneva: WHO; [cited 2025 Jun 2]. Available from: https://www.who.int/disabilities/actionplan/en/
6. Wong FKY, Liu H, Wang H, Anderson D, Seib C, Molasiotis A. Global nursing issues and development: An analysis of World Health Organization documents. J Nurs Scholarsh.

2015;47(6):574–83.

7. Morin KH. Evolving global education standards for nurses and midwives. MCN Am J Matern

Child Nurs. 2012;37(6):360–6.

8. Slusher TM, Kiragu AW, Day LT, Bjorklund AR, Shirk A, Johannsen C, et al. Pediatric critical care in resource-limited settings—Overview and lessons learned. Front Pediatr.

2018;6:49.

9. Pacific Bridge Medical. Healthcare in Laos [Internet]. 2017 Jul 11 [cited 2025 Jun 2].

Available from: https://www.pacificbridgemedical.com/publication/healthcare-in-laos/

10. Barron A, Moran J, Nina S, Harlow J, Gyawali M, Hossain F, et al. Building specialized nursing practice capacity in Bangladesh: An educational program to prepare nurses to care for oncology and bone marrow transplant patients in Dhaka, Bangladesh. J Glob Oncol.

2018;4(4):1–6.

11. Halabi JO, Abdalrahim MS, Persson GL, Hedemalm A, Lepp M. The development of a preceptor training program on clinical nursing education in Jordan in collaboration with Sweden.

J Contin Educ Nurs. 2012;43(3):135–44.

12. Kumar R, Canarie M. Developing pediatric critical care in Kenya. Pediatr Crit Care Med.

2019;20(12):e538.

13. McConnell KA, Krisher LK, Lenssen M, Bunik M, Bunge Montes S, Domek GJ. Telehealth to expand community health nurse education in rural Guatemala: A pilot feasibility and acceptability evaluation. Front Public Health. 2017;5:60.
14. Harris PA, Taylor R, Thielke R, Payne J, Gonzalez N, Conde JG. Research electronic data capture (REDCap) – A metadata-driven methodology and workflow process for providing translational research informatics support. J Biomed Inform. 2009;42(2):377–81.
15. Harris PA, Taylor R, Minor BL, Elliott V, Fernandez M, O’Neal L, et al. The REDCap consortium: Building an international community of software partners. J Biomed Inform.

2019;103208.

16. Leininger M. Leininger's theory of nursing: Cultural care diversity and universality. Nurs Sci Q. 1988;1(4):152–60.
17. Sullivan B, Bettger J. Community-informed health promotion to improve health behaviors in Honduras. J Transcult Nurs. 2018;29(1):14–20.
18. Trint Ltd. Trint Platform [Internet]. [cited 2020]. Available from: https://www.trint.com/
19. Bell S, Bam V, Acheampong E. Developments in emergency nursing education in Ghana.

Emerg Nurse. 2015;23(8):18–21.

20. Canarie M, Shenoi A. Teaching the principles of pediatric critical care to non-intensivists in resource limited settings: Challenges and opportunities. Front Pediatr. 2018;6:44.
21. Ralston M, Day L, Slusher T, Musa N, Doss H. Global paediatric advanced life support:

Improving child survival in limited-resource settings. Lancet. 2013;381(9862):256–65.

22. Shilkofski N, Hunt E. Identification of barriers to pediatric care in limited-resource settings:

A simulation study. Pediatrics. 2015;136(6):e1569–75.

23. Thurmond V. The point of triangulation. J Nurs Scholarsh. 2001;33(3):253–8.
24. Melnyk BM, Fineout-Overholt E. Evidence-based practice in nursing & healthcare: A guide to best practice. 4th ed. Philadelphia: Wolters Kluwer; 2019.

Figure	[Click here to access/download;Figure;Fig 1.tiff](https://www2.cloud.editorialmanager.com/pone/download.aspx?id=41691942&guid=6a9ed729-4312-46c7-baf1-bab0b2c72e78&scheme=1)

[Click here to access/download](https://www2.cloud.editorialmanager.com/pone/download.aspx?id=41691972&guid=84ace404-18ec-49c0-9c5b-aa4ed9342bc7&scheme=1)

[Supporting Information](https://www2.cloud.editorialmanager.com/pone/download.aspx?id=41691972&guid=84ace404-18ec-49c0-9c5b-aa4ed9342bc7&scheme=1)

[N8105.LikertQuestions_DATA_2021-02-07_1203.xls](https://www2.cloud.editorialmanager.com/pone/download.aspx?id=41691972&guid=84ace404-18ec-49c0-9c5b-aa4ed9342bc7&scheme=1)

[Click here to access/download](https://www2.cloud.editorialmanager.com/pone/download.aspx?id=41691973&guid=930612ff-1c82-4d4d-9387-96c11da3237e&scheme=1)

[Supporting Information](https://www2.cloud.editorialmanager.com/pone/download.aspx?id=41691973&guid=930612ff-1c82-4d4d-9387-96c11da3237e&scheme=1)

[N8105.NursingSkillsAnalysi_DATA_2021-02-07_1151.xlt](https://www2.cloud.editorialmanager.com/pone/download.aspx?id=41691973&guid=930612ff-1c82-4d4d-9387-96c11da3237e&scheme=1)

Revised Manuscript with Track Changes

1

2

3

4

5

6

7

8

9 Pediatric Capacity Building in Northern Laos: An Evaluation of the Pediatric
10 Nursing Training Program at Lao Friends Hospital for Children

11

12

13

14

15

16	**Short title**: Pediatric Nursing Capacity Building in Laos

17

18 **Authors**:
19 Rebecca SILVERS DNP, APN, CPNP-AC, CCRN, RNFA^[4]^*;
20 Maly CHITTAPHAI RN^[5]^;
21 Matt EVANS, MS, MPH, RN ^2^;
22 Maddie WONG, MS^1^;
23 Jessica VAN METER, DNP, RN, APN-BC, CCRN, A-EMT^[6]^ 24
25 **Maly Chittaphai:** Conceptualization (support), resources (support), review, and editing (equal).
26 **Matt Evans**: Conceptualization (support), resources (lead), review, and editing (equal). **Rebecca**
27 **Silvers**: Conceptualization (lead); Methodology (lead), writing – original draft (lead); formal
28 analysis (lead); writing – review and editing (equal). **Jessica Van Meter**: Conceptualization
29 (supporting); Writing – original draft (supporting); Writing – review and editing (equal). **Maddie**
30 **Wong**: Writing – review and editing (equal)

31

32	**Conflict of Interest**: The authors deny any conflicts of interest.

33

34 ***Corresponding Author:**
35 Rebecca.silvers@ucsf.edu (RS)

36

37

38

39

40

## ABSTRACT

**Background**: In 2015, the child mortality rate in Laos was 53.6 per 1,000 live births. The Lao Friends Hospital for Children (LFHC) in Luang Prabang was established to enhance access to specialized pediatric care. To support workforce development, a Pediatric Nursing Training Program was implemented. However, the program has not undergone a formal evaluation since its inception. This study aims to evaluate the program’s first five years.

**Study Design**: The evaluation included a review of curriculum documents via an online platform and feedback collection through an online survey administered to former expatriate nurse volunteers who provided clinical mentorship. Additionally, semi-structured interviews were conducted with former nurse leaders who designed and implemented the program. Qualitative data were analyzed thematically and validated through triangulation.

**Results**: The curriculum review revealed system-based and course-specific plans, including clear objectives, teaching methods, and evaluation tools. Of the 148 clinical mentors surveyed, a 45% response rate was achieved. Survey responses indicated improvements in nurses’ clinical skills since the program’s inception, with mentors emphasizing the importance of cultural attunement to Lao staff. Six out of seven former nurse leaders participated in interviews. Key themes emerged, highlighting the value of culturally relevant educational materials, multimodal teaching strategies, a train-the-trainer approach, and clinical mentorship as integral components of the program.

**Conclusion**: The development of pediatric nursing specialization at LFHC was facilitated by structured curriculum plans, cultural sensitivity, diverse teaching methodologies, and robust clinical mentorship. These findings provide valuable insights for designing culturally competent and sustainable training programs in Laos and similar contexts.

**Keywords**: knowledge to practice gap, pediatric acute care, nurse training program, clinical mentorship, cultural humility

### 1. Introduction

Child mortality worldwide has reduced dramatically over the last few decades. In 1990, one in 11 children died before reaching age five, compared to one in 26 in 2017 [1]. Although the global rate of child mortality is on a decline, there remains a great deal of variation in death rates across geographical locations. Ninety-five percent of pediatric deaths occur in low- and middle-income countries [2]. In 2017, the number of under-5 deaths was 5.4 million globally; half of those deaths were children in sub-Saharan Africa, and another 30% were children in Southern Asia [1].

Access to appropriate pediatric care is the largest determinant of child morbidity and mortality, and therefore, understanding access to care is essential to uncovering solutions worldwide [3]. For a country or region to provide access to pediatric health services, a system must optimize the number of clinicians and their clinical expertise, environmental and physical resources, and address patient variables and barriers to care. In addition, clinicians must be prepared with the clinical skills and knowledge to make appropriate clinical judgments in pediatric care [4].

There are numerous challenges in low- and middle-income countries (LMICs) to optimizing the nursing workforce to provide access to pediatric care. Additionally, high-quality professional nursing education in LMICs faces many challenges, including a lack of available faculty, limited clinical preceptorship hours, and failure of available evidence for best practice to apply to their clinical settings [5,6]. As a result, nurses struggle to apply what they have learned with available resources and standards of practice in their local setting, which results in a lack of standardized care and poor confidence in nursing practice [7]. Thirdly, the opportunities for specialized pediatric training are limited [8].

### 2. Background

Laos, a landlocked country in Southeast Asia, is characterized by mountainous terrain and significant ethnic, religious, and linguistic diversity. Despite its rich cultural heritage, Laos faces considerable economic challenges and is classified by the World Bank as a low-income country. Its healthcare infrastructure is still developing, particularly in rural areas outside the capital city. To bridge gaps in healthcare delivery, various organizations in Laos partner with international aid agencies and non-governmental organizations (NGOs) to strengthen healthcare programs and enhance the education of medical professionals [9].

Efforts to improve healthcare in low- and middle-income countries (LMICs) like Laos have increasingly focused on supplementary education and training. Academic institutions and NGOs play a pivotal role in elevating clinical practice standards by providing specialized training for healthcare workers [10-13]. These initiatives aim to address critical shortages in healthcare workforce expertise and improve health outcomes in resource-limited settings.

In 2014, the non-governmental organization Friends Without Borders (FWAB), in collaboration with the Ministry of Health, launched an initiative to expand access to pediatric specialty care in northern Laos. This effort led to the establishment of a pediatric specialty medical center aimed at addressing critical healthcare gaps and reducing the child mortality rate, which was 53.6 per 1,000 live births. A key focus of the initiative was capacity building through comprehensive training programs for Lao healthcare professionals—including doctors, nurses, allied health workers, and administrative staff—to strengthen local expertise and improve health outcomes.

The Lao Friends Hospital for Children (LFHC) opened its doors in January 2015, alongside the launch of the Pediatric Nursing Training Program. This program was designed to enhance the clinical competencies of nursing staff through culturally tailored didactic education and bedside clinical mentorship. The training program is supported by expatriate nurse leaders who commit to one- to two-year terms, as well as short-term nursing volunteers who provide bedside mentorship during their one- to twelve-month placements. Didactic sessions take place in the hospital’s designated learning spaces, with protected time allocated for nurses to participate in educational activities.

The pediatric nursing training program at LFHC is the first of its kind in Laos, yet a program evaluation has not been conducted. Thus, the purpose of this study was to evaluate the first five years of the specialized nursing training program. Aims including evaluating whether the program implemented culturally relevant training and increased nurses’ clinical knowledge and their ability to apply their knowledge within the clinical setting.

### 3. Methods

#### 3.1 Study Setting & Design

This study was conducted at the Lao Friends Hospital for Children (LFHC), a no-cost tertiary pediatric referral facility located in Luang Prabang, northern Laos. According to

UNICEF estimates, the pediatric population in Laos is approximately 2.8 million; however,

LFHC is one of only two dedicated pediatric hospitals in the country. Since opening its doors in 2015 with an outpatient clinic and inpatient wards, LFHC has expanded to provide a full range of specialized services, including surgery, neonatal, emergency, and critical care, a developmental clinic, and a thalassemia program. Annually, the hospital now serves more than 45,000 children from across northern Laos and has provided access to care for over 200,000 patients in the first

~~voluntary, and respondents were not required to submit identifiable information. The survey was~~ ~~administered in English. A pilot test was conducted to enhance the face and content validity of~~ ~~the survey before it was distributed.~~

~~A separate email was sent to eligible nursing directors and nurse educators inviting them~~ ~~to participate in a semi-structured interview.~~ Participants were informed that their consent to participate in the program evaluation was implied through their response to the email invitation.

Interviews with former nurse leaders were scheduled at a time convenient for each participant.

The interviews were conducted via Zoom and were recorded with the participants' permission.

Each interview was designed to last approximately one hour.

#### 3.4 Data Tools and Collection

Data collection occurred simultaneously through a review of curriculum documents, a

REDCap survey, and through semi-structured interviews over a four-week period. The LFHC Nurse Training Program curriculum and other relevant documents were gathered and reviewed to evaluate the following themes: program design, program objectives, training schedule, and the content of the program. Attention to the train-the-trainer model and cultural relevance was also noted.

**Formatted:**

Space

After:

0

pt

The LFHC Clinical Mentor Survey consisted of three sections and was distributed in

January 2021 to all expatriate nurses who participated in the clinical mentorship program from

2015 to 2019. The first section gathered information about participants’ nursing background, clinical experience prior to LFHC, and any prior experience working abroad. The second section focused on the expatriate nurses’ perspectives regarding the clinical skill set of the Lao staff nurses during their time at LFHC. Ratings were done based on recall of past experiences of direct observation. The final section provided an opportunity for self-reflection on the expatriate nurses’ contributions to the training process, specifically in clinical mentoring and cultural competence. A pilot test was conducted to enhance the face and content validity of the survey before it was distributed. All the data collected from the survey were imported via REDCap into a Microsoft Excel spreadsheet for analysis.

~~Participants were informed that their consent to participate in the program evaluation was~~ ~~implied through their response to the email invitation.~~ Interviews with former nurse leaders were scheduled at a time convenient for each participant. The interviews were conducted via Zoom and were recorded with the participants' permission. Each interview was designed to last approximately one hour. The LFHC Program Evaluation Interview Guide was used for the semistructured interviews and consisted of 14 questions ~~used to guide semi-structured interviews with~~ ~~former nursing leadership~~ (Fig. 1). Two questions collected demographic data about the nurse leaders, while the remaining 12 questions focused on evaluating the program. These questions encouraged participants to reflect on their role in facilitating the LFHC Nurse Training Program. To ensure content and face validity, the interview guide was tested by the r~~R~~esearch ~~Committee~~ team at Vanderbilt University. The interviews were recorded by the interviewer and submitted to

**Formatted:**

Line

spacing:

Double

Trint Ltd., an online high-quality and secure transcription service, to be transcribed [18].

Fig 1. Interview Guide

#### 3.5 Data Analysis

Data collected from the electronic REDCap survey were analyzed using descriptive statistics. Demographic variables were summarized as frequencies and percentages. Likert-scale responses were analyzed in Microsoft Excel using built-in functions (AVERAGE and STDEV) to calculate mean scores and standard deviations. For the qualitative component, interviews were audiorecorded, transcribed verbatim, and anonymized prior to analysis. Data were examined using thematic analysis informed by grounded theory methodology. Coding was performed manually by two members of the research team, followed by review with the Principal Investigator. The coding process involved open coding to identify initial concepts, axial coding to organize these into categories, and iterative refinement of emerging themes and subthemes. Discrepancies were resolved through consensus meetings, and an audit trail of coding decisions was maintained. Triangulation across coders and data sources enhanced credibility, and cross-validation of findings strengthened the rigor and trustworthiness of the analysis. ~~Data collected from the~~ ~~electronic REDCap survey were analyzed utilizing descriptive statistics. Each demographic~~ ~~variable was evaluated and described using the number of participants in each category, along~~ ~~with corresponding percentages. Variables measured on the Likert scale were analyzed using~~ ~~mean scores and standard deviations. The qualitative data from the interviews were transcribed~~ ~~and analyzed through thematic analysis using grounded theory methodology. This process was~~ ~~conducted by two members of the Research Committee at Vanderbilt University, in addition to~~ ~~the Principal Investigator, to ensure triangulation and validity of the results. The collaborative~~ ~~analysis helped to confirm the consistency of findings across different data sources, enhancing~~ ~~the reliability and validity of the study through cross-validation and multiple perspectives.~~

**Formatted:**

Indent:

First

line:

0.5

"

#### 3.6 Ethical Considerations

This study received ethical approval from the Institutional Review Board (IRB) at Vanderbilt University under exempt (non-research) status (Protocol #202298). Additional approval to proceed with the study was granted by the Lao Friends Hospital for Children (LFHC) Research Committee following review of the study protocol.~~Additional approval was provided~~ ~~by the Lao Friends Hospital for Children’s Research Committee .~~ Participation in the study was entirely voluntary. Consent was implied through the completion of surveys and participation in interviews or focus groups, with participants informed of their right to withdraw at any time without penalty. No financial incentives or compensation were provided. All data were anonymized to safeguard confidentiality, and results are reported in aggregate to protect participant identity. Audio recordings of interviews and focus groups were obtained only with consent and remain securely stored, with assurance of non-disclosure beyond the research team. ~~Participation in the study was voluntary, and participants were informed that consent would be~~

~~implied through their completion of the survey and interviews.~~

### 4. Results

#### 4.1 Curriculum Documents Review

The curriculum documents were stored on a privately accessed Microsoft OneDrive, with access controlled by the head of information technology at LFHC. The OneDrive contains organized folders, including audits, competency, courses, curricula, English vocabulary list, exams, in-services, medication safety, emergency simulations, nursing orientation, students, and training records, each with several subfolders. A review of the curriculum documents revealed thorough and well-organized record-keeping of clinical courses taught as part of the education and training programs since 2016. The training documents and PowerPoint presentations for each course were easily accessible in their designated subfolders. Lesson plans clearly outline the targeted learning objectives for each course, along with the associated evaluation methods, which include hands-on activities and group exercises. The clinical competency evaluations were clearly documented for each nurse at LFHC, but the schedule for student reassessment or evaluation was unclear. It was also unclear if there was testing to measure expatriate nurses’ understanding of the content that they were teaching.

#### 4.2 LFHC Clinical Mentorship Survey

The LFHC Clinical Mentorship Survey was distributed to 148 former nursing volunteers who served from 2015 to 2019. Sixty-seven of the volunteers responded and completed the survey in full for a return rate of 45%.

##### 4.2.1 Expatriate Nurse Demographics

The first section of the survey focused on the demographics and work history of expatriate nursing volunteers (Fig. 2). A total of 67 survey responses were analyzed, revealing participation across all years of the Pediatric Nursing Training Program's initial operation. The majority of respondents volunteered in 2017 (n=19) and 2018 (n=17), with additional participation in 2015 (n=6), 2016 (n=10), and 2019 (n=15). Regarding the duration of service at LFHC, most respondents (64.2%) reported volunteering for two to six months (n=43). Seventeen volunteers (25.4%) stayed for one month, five (7.5%) for six months to one year, and two (3%) for more than one year.

The participants represented a diverse range of countries, with the majority identifying as Australian (n=21), American (n=19), British (n=11), Canadian (n=8), or New Zealander (n=3).

Additional countries of origin included Italy (n=2), Spain, Portugal, and Denmark (n=1 each). Notably, 41 respondents (61%) had prior experience in resource-variable settings (RVS).

Forty-eight expatriate volunteers (71.5%) identified as bedside registered nurses, while others reported varied roles, including clinical nurse specialists (13.4%, n=9), nurse educators (11.9%, n=8), advanced practice nurses or nurse practitioners (7.5%, n=5), operating room nurses (4.5%, n=3), professors at university nursing schools (3%, n=2), and nurse anesthetists (1.5%, n=1).

Participants also had diverse clinical backgrounds. More than half (50.7%, n=34) had experience in pediatric wards. Other areas of clinical experience included emergency departments (38.8%, n=26), pediatric-specific emergency departments (27.3%, n=25), pediatric critical care (26.9%, n=18), pediatric clinics (20.9%, n=14), neonatal intensive care (17.9%, n=12), and pediatric hematology and oncology (16.4%, n=11).

The respondents varied in their years of clinical nursing experience before joining LFHC: 17 (25.4%) had one to five years of experience, 22 (32.8%) had five to ten years, nine (13.4%) had 10 to 15 years, and 19 (28.4%) had over 15 years of experience.

322	**Table 1. Demographics of Participants: LFHC Clinical Mentorship Survey.**

| *Demographic Data (N=67)* |  |
| --- | --- |
| **Start participating at LFHC (Year)** | **n (%)** |
| 2015 | 6 (9) |
| 2016 | 10 (14.9) |
| 2017 | 19 (28.4) |
| 2018 | 17 (25.4) |
| 2019 | 15 (22.4) |
| **Length of time at LFHC** |  |
| <1 month | 17 (25.4) |
| 2-6 months | 43 (64.2) |
| 6 months – 1 year | 5 (7.5) |
| >1 year | 2 (3) |
| **Age (years)** |  |
| 21-30 | 32 (47.8) |
| 31-39 | 14 (20.9) |
| 40-49 | 5 (7.5) |
| 50-59 | 11 (16.4) |
| >59 | 5 (7.5) |
| **Home Country** |  |
| Australia | 21 (31.3) |
| United States | 19 (28.4) |
| United Kingdom | 11 (16.4) |
| Canada | 8 (11.9) |
| New Zealand | 3 (4.5) |
| Other ^a^ | 5 (7.5) |
| **Profession ^b^** |  |
| Bedside Nurse | 48 (71.6) |
| Clinical Nurse Specialist | 9 (13.4) |
| Nurse Educator | 8 (11.9) |
| Advance Practice Nurse (Nurse Practitioner) | 5 (7.5) |
| Operating Room/Theater Nurse | 3 (4.5) |
| Professor/Teacher at School of Nursing | 2 (3) |
| Nurse Anesthetist | 1 (1.5) |
| Other ^c^ | 6 (9) |
| **Clinical Experience (years)** |  |
| 1 - 5 | 17 (25.4) |
| 5- 10 | 22 (32.8) |
| 10-15 | 9 (13.4) |
| >15 | 19 (28.4) |
| **Previous experience in resource-limited setting** |  |
| Yes | 41 (61.2) |
| No | 26 (38.8) |
| **# countries experience working** |  |

| None | 26 (38.8) |
| --- | --- |
| 1-3 | 30 (44.8) |
| 3-10 | 9 (13.4) |
| >10 | 2 (3) |
| **Clinical experience ^a^** |  |
| Pediatric Ward | 34 (50.7) |
| Emergency department (A & E) | 26 (38.8) |
| Pediatric specific ED (A & E) | 25 (37.3) |
| Pediatric Critical Care (PICU) | 18 (26.9) |
| Pediatric Clinic | 14 (20.9) |
| Neonatal Intensive Care | 12 (17.9) |
| Pediatric Hematology & Oncology | 11 (16.4) |
| Other *** | 20 (29.9) |

^a^ Other includes Italy (n=2), Spain, Portugal, & Denmark ^b^ Participants were asked to mark all answers that apply ^c^ Other includes physician assistant, nurse manager (NICU), clinical nurse consultant (n=2), research ^d^ Other includes stroke triage, PACU (pediatric recovery room) (n=2), remote First Nations Primary Care Health Centre, Trauma, adult ICU (n=3), adult medical & surgical wards (n=7), school nurse in a public school, acute rehabilitation (n=3), hospice, Operating Theatre (n=3),

Labor and Delivery (Maternity) (n=2)

##### 4.2.2 Evaluation of the Content of the Training Program

In the second section of the LFHC Clinical Mentorship Survey, participants rated the Lao nursing staff on 14 skills using a Likert scale ranging from one to five where one referred to insufficient knowledge and being unable to complete the skill, while five referred to completing competence in knowledge and skill and being able to help support others (Table 2). The mean (standard deviation) of the clinical skill set rated by the participants in 2015 was 2.8 (0.4). Over the course of five years, the mean increased to 3.5 (0.5) in 2019. The greatest changes in mean scores between 2015 and 2019 were starting an IV live (1.1), recognition of a patient having a seizure (1), application of weight-based medication dosing (0.8), and application of appropriate respiratory support for hypoxia (0.8). The smallest change in mean scores was seen in the knowledge of normal vital signs for age (0.2), performing a 12-lead ECG (0.3), and recognition of a patient in respiratory distress or failure (0.3).

### 345 Table 2. LFHC Clinical Mentorship Survey: Nursing Clinical Skill Data by Year

| **Nursing Clinical Skills** | **2015** | **2016** | **2017** | **2018** | **2019** |
| --- | --- | --- | --- | --- | --- |
| Taking vital signs on a pediatric patient | 3.3 | 3.7 | 3.6 | 3.6 | 3.8 |
| Knowledge of normal vital signs patients of various ages | 3 | 2.9 | 3.1 | 3.4 | 3.2 |
| Drawing blood through a venipuncture | 3.7 | 4 | 4 | 4.3 | 4.4 |
| Starting an intravenous (IV) line to administer intravenous fluids/medications | 3 | 4.1 | 3.9 | 4.1 | 4.1 |
| Using a stethoscope to perform a respiratory examination | 2.5 | 3.1 | 2.7 | 2.9 | 3.2 |
| Application of weight-based medication dosing | 2.7 | 2.8 | 2.7 | 3.1 | 3.5 |
| Preparing and administering oral medications | 3.2 | 3.7 | 3.5 | 3.6 | 3.9 |
| Preparing and administering IV administration | 2.8 | 3.5 | 3.3 | 3.4 | 3.5 |
| Performing an electrocardiogram (EKG or ECG) | 2.5 | 2.7 | 2.7 | 2.8 | 2.8 |
| Recognition of hemodynamically unstable patient | 2.3 | 2.6 | 2.5 | 2.8 | 2.8 |
| Recognition of a patient in respiratory distress or failure | 2.8 | 3.1 | 2.9 | 3.2 | 3.1 |
| Application of the appropriate oxygenation delivery devise for hypoxemia/respiratory distress | 2.7 | 2.8 | 2.9 | 3.2 | 3.5 |
| Recognition of a child with seizures | 2.3 | 3.1 | 3.1 | 3.2 | 3.3 |
| Participation in pediatric or neonatal cardiopulmonary resuscitation (CPR) | 2.7 | 2.9 | 2.8 | 3.1 | 3.3 |
| **Mean** | **2.8** | **3.2** | **3.1** | **3.3** | **3.5** |
| **Standard Deviation** | 0.4 | 0.5 | 0.5 | 0.5 | 0.5 |

#### *4.2.3 Participants’ Self-Reflection on Contributions to the Training Program*

The third section of the survey focused on the participants' self-reflection on their contribution to the training process pertaining to clinical mentoring and cultural competence. The nurses responded to 14 statements on a Likert scale of one to five, where one was “strongly disagreed” and five was “strongly agree.” Eighty-four percent (n=56) of participants agreed or strongly agreed that their knowledge of Lao culture contributed to their ability to provide clinical mentorship and education to the LFHC nursing staff at the bedside. The majority also agreed or strongly agreed (90%, n=60) that participating in cultural events in the hospital and town increased their understanding go Lao values and beliefs. Additionally, 96% (n=64) of volunteers responded that they agree or strongly agree that as their time at LFHC progressed, they could recognize barriers to learning in Lao and adapt their teaching methods.

Sixty-one percent (n=41) of participants strongly agreed they were able to facilitate more constructive clinical mentorship as the strength of their relationship with the Lao staff strengthened (mean 4.5, SD 0.8). The participants agreed that they recognized the importance of cultural competence in the nursing training program (mean 4.6, SD 0.5). 96% (n=64) of the participants strongly agreed or agreed that they understood the role of nursing in Laos at the end of their time in LFHC (mean 4.4, SD 0.6). Fifty-five percent of participants agreed or strongly disagreed with understanding the role of nursing in Laos prior to volunteering at LFHC (mean

3.5, SD 1.0).

There was neither an agreement or disagreement (mean 3.0, SD 1.0) that the Lao nurses struggled to apply their classroom education to the clinical setting, but 93% (n=62) of the participants agreed or strongly agreed that they felt comfortable recognizing the knowledge to practice gap and addressing it as they mentored Lao staff at the bedside (mean 3.9, SD 0.6). Overall, the clinical mentors felt that their time working with the Lao nurses contributed to an increase in the clinical skills of the Lao staff (mean 3.9, SD 0.7).

#### 4.3 LFHC Program Evaluation: Semi-Structured Interviews

##### 4.3.1 Demographics

During the first two years of the program at LFHC, a nursing director managed both the administrative and educational programs for the nurses, and in the latter three years, there was an additional role of a nurse educator (Participant 1). Semi-structured interviews were conducted with six former nurse leaders at LFHC. Two of the nurse leaders were from Australia; one was from the United Kingdom, one was from New Zealand, one was from Canada, and one was from the United States. The amount of time each nurse leader worked at LFCH ranged from nine months to two years. Based on the content analysis of the interview transcripts, many themes

(

italicized below) emerged related to the following topics: nursing curriculum development,

381

clinical mentors

hip program, and cultural competence.

382

*4*

*.3.2*

*Nursing Curriculum Development*

383

*Continual Revision of Curriculum*

384

Participants described the evolution of the nursing curriculum as a dynamic and

385

responsive process. In the early years of hospital development,

training began with a brief WHO

386

course, which several participants felt was too limited. One explained, “The initial training was

387

only a short course based on the WHO’s little blue book” (Participant 2). Over time, the

388

curriculum expanded with input from g

lobal sources. As another noted, “We adapted from WHO

389

and MSF system

-

based curricula, but always tried to fit them to our context” (Participant 3).

390

The onboarding of a formal nurse educator in 2016 marked a turning point. According to

391

one participant, “Wi

th a dedicated educator, we could customize the curriculum based on the Lao

392

nurses’ skill set and what we had learned in the first two years” (Participant 1). This shift

393

included the deliberate removal of irrelevant material. “We eliminated content written

for high

-

394

income countries that did not make sense here” (Participant 4). Curriculum revision became a

395

regular process, with each new educator reassessing practice and clinical goals. “Every one or

396

two years, when a new educator joined, they looked at what

the staff needed and adjusted the

397

content” (Participant 1). Lao nurse educators also played a critical role once they began teaching

398

independently. One participant highlighted, “They were encouraged to change the curriculum

399

themselves, based on whether it

met the needs of their colleagues” (Participant 5).

400

*Adaptive Teaching Style Grounded in Humility*

401

Alongside curriculum changes, participants emphasized the importance of adapting

402

teaching styles to meet the learning preferences of Lao nurses. Early experi

ences revealed

403

**Formatted:**

Font:

Not

Bold

**Formatted:**

Space

After:

0

pt,

Line

spacing:

Double

**Formatted:**

Font:

Not

Italic

**Formatted:**

Space

After:

0

pt

**Formatted:**

Font:

Not

Italic

**Formatted:**

Font:

Not

Bold

**Formatted:**

Space

After:

0

pt,

Line

spacing:

Double

**Formatted:**

Font:

Not

Italic

mismatches between Western teaching expectations and local educational norms. “The nurses

404

were used to memorizing and repeating answers; they had never been asked to question or

405

participate before” (Participant 5). To address this, educators

experimented with different

406

methods. One described, “We used a trial and error system to find what worked. Interactive

407

games ended up being very effective” (Participant 1).

408

Tone and demeanor were also crucial. As one educator explained, “The nurses were

very

409

sensitive to the teacher’s tone and frustrations. They responded best to a compassionate,

410

understanding style” (Participant 1). Participants consistently described the importance of

411

humility in teaching, recognizing that respectful and culturally sens

itive interactions were key to

412

sustaining engagement.

413

*Creating a Culture of Critical Thinking*

414

A consistent theme across interviews was the need to move beyond rote memorization

415

and cultivate critical thinking. Participants spoke about historical barriers.

“Nursing education in

416

Laos had been focused on memorization and tasks, not questioning or inquiry” (Participant 4).

417

This approach left gaps in clinical reasoning. “The nurses were task compliant, but they did not

418

always understand how interventions impact

ed the patient’s health” (Participant 3).

419

Several leaders provided striking examples. One recalled, “There were fundamental knowledge

420

gaps, such as not connecting the pulse with the beating of the heart” (Participant 1). For the nurse

421

leaders, teaching cri

tical thinking was not only about knowledge but about transforming practice.

422

As one summarized, “Our goal was to build clinical inquiry, so they could trust their assessments

423

and contribute to planning care with the team” (Participant 4).

424

*Nursing Empowerme*

*nt and Sustainability*

425

**Formatted:**

Font:

Not

Italic

**Formatted:**

Font:

Not

Bold

**Formatted:**

Space

After:

0

pt,

Line

spacing:

Double

**Formatted:**

Font:

Not

Italic

**Formatted:**

Font:

Not

Bold

~~nurses were sensitive to the teacher's tone and frustrations; they responded well to a~~ ~~compassionate teaching style with understanding from their teacher (Participant 1).~~

*~~Creating a Culture of Critical Thinking.~~* ~~All the interviewed nurse leaders spoke about~~ ~~the desire to bring critical thinking to LFHC. Historically, nursing education in Laos emphasized~~ ~~rote memorization and task-oriented practice, discouraging inquiry or questioning. This approach~~ ~~hindered the development of essential clinical processes, such as assessment, diagnosis,~~ ~~planning, implementation, and evaluation (Participant 4). One nurse leader described the Lao~~ ~~nurses as highly task-compliant but noted that their understanding of how interventions impacted~~ ~~patient health was limited (Participant 3). Another provided an example of fundamental~~ ~~knowledge gaps, such as the inability to connect the pulse to the heart's beating (Participant 1).~~ ~~Critical thinking was viewed as pivotal to advancing pediatric nursing care. The overarching goal~~ ~~of the training program was to cultivate clinical inquiry, enabling Lao nurses to trust their~~ ~~assessments and actively engage in care planning with interdisciplinary teams.~~

*~~Nursing Empowerment & Sustainability.~~* ~~Initially, Lao nurses at LFHC demonstrated low~~ ~~confidence when faced with significant challenges or setbacks (Participant 3). Given that LFHC's~~ ~~long-term plan involved transitioning the hospital to the Lao Ministry of Health after 10 years, a~~ ~~core objective for nurse leaders was to empower the local nursing staff to eventually assume~~ ~~leadership roles. This empowerment strategy centered on fostering autonomy and confidence~~ ~~among the nurses. To build empowerment, nurse leaders encouraged Lao nurses to voice~~ ~~concerns or suggest improvements in patient care (Participant 1). A key component of this effort~~ ~~was the planned implementation of a modified train-the-trainer model. Over two years of training~~ ~~and observation, future nurse leaders were identified based on their clinical expertise and~~ ~~emerging leadership potential (Participant 1). Despite this progress, a significant barrier to~~

successful” (Participant 3). One participant added, “In Lao culture, you do not raise your voice.

526

The mentors who adapted t

o that had better results” (Participant 4). Language barriers also

527

underscored the need for humility. “Medical terms do not always translate. The best mentors

528

admitted when things were unclear and worked together to find simpler explanations”

529

(

Participant

6)

. Ultimately, humility built trust. “The most successful mentors created safe

530

relationships where nurses felt

comfortable saying what they did not understand” (Participant 6).

531

~~Clinical mentorship was an essential part of the training program~~

~~,~~

~~and the role of the~~

532

~~clinical mentor changed as the nursing education and clinical scope of LFHC developed.~~

533

~~Initially, their role in the inpatient unit focused on~~

~~clinical training and patient safety support for~~

534

~~all of the nursing staff~~

~~. In~~

~~late~~

~~2017~~

~~, howe~~

~~ver, the Lao nursing competence rose~~

~~,~~

~~and Lao shift~~

535

~~leaders were appointed. At that time, clinical mentors worked side by side with nurses who~~

~~m~~

~~the~~

536

~~nursing director or educator identified as needing support (~~

~~Participant 4~~

~~)~~

~~. Clinic mentors worked~~

537

~~in the inp~~

~~atient department, neonatal unit, operating theater, and emergency department with a~~

538

~~full~~

~~-~~

~~time shift schedule that mimicked the Lao staff schedule.~~

539

~~Choosing Clinical Mentors~~

~~.~~

~~It was the nursing director’s responsibility to select the~~

540

~~clinical mentors for~~

~~LFHC, and volunteers were initially recruited through the London School of~~

541

~~Hygiene and Tropical Medicine~~

~~and Liverpool School of Tropical Medicine~~

~~. Nurses who took~~

542

~~the tropical medicine course at the school~~

~~s~~

~~also received training on working in resource~~

~~-~~

~~va~~

~~riable~~

543

~~areas and were familiar with the pathophysiology of childhood disease in Laos (~~

~~Participant 2~~

~~.~~

~~)~~

544

~~Additionally, many of the volunteer nurses came from a former clinical mentor referral~~

545

~~(~~

~~Participant 3~~

~~)~~

~~. Prospective volunteers were interviewed by the~~

~~nursing directors via Skype or~~

546

~~Zoom, and preference was given to those who had prior experience working in resource~~

~~-~~

~~variable~~

547

~~settings (~~

~~Participant 2~~

~~. Interviewe~~

~~)~~

~~e~~

~~s also highly regarded open~~

~~-~~

~~mindedness and adaptability~~

548

**Formatted:**

Font:

Not

Bold,

Not

Italic

**Formatted:**

Font:

Not

Bold,

Not

Italic

**Formatted:**

Font:

Not

Italic

**Formatted:**

Font:

Not

Bold

~~among selected volunteers. One intervi~~

~~ewee observed that intensive care nurses were less~~

549

~~successful in the role of clinical mentors at LFHC due to challenges in adapting to the dynamic~~

550

~~and unpredictable nature of the environment, whereas emergency room nurses were better~~

551

~~equipped to handle suc~~

~~h variability due to their experience in managing diverse situations and~~

552

~~urgent cases~~

~~(~~

~~Participant 4~~

~~).~~

553

~~Mentoring the Mentors~~

~~.~~

~~Each of the interviewed nursing leaders alluded to the need for~~

554

~~ongoing support for the clinical mentors~~

~~. Existing support resour~~

~~ces included~~

~~a pre~~

~~-~~

~~departure~~

555

~~orientation packet that con~~

~~sisted~~

~~of a brief description of Lao culture, their role at LFHC, and~~

556

~~detailed information about Laos (~~

~~Participant 4~~

~~. Clinical mentors also spent a day with a nursing~~

~~)~~

557

~~leader and a seasoned volunteer~~

~~(~~

~~Participant 1~~

~~. Additional opportunities for clinical mentors to~~

~~)~~

558

~~learn about Laos and LFHC included an optional one~~

~~-~~

~~day with the outreach team, who drove into~~

559

~~the villages to provide~~

~~education, support, and~~

~~care (~~

~~Participant 1~~

~~.~~

~~)~~

~~Clinical mentors~~

~~were also~~

560

~~invited into the Lao staff~~

~~’~~

~~s classroom training, and they were provided language learning time on~~

561

~~campus (~~

~~Participant 3~~

~~).~~

562

~~Ongoing support for the mentors through counseling during their time at LFHC was essential to~~

563

~~the nurse leaders (~~

~~Participant 2~~

~~)~~

~~. Each~~

~~of the nurse leaders reported an open~~

~~-~~

~~door policy for~~

564

~~mentors to come to speak to them and all of the nurse leaders found that listening and informal~~

565

~~debriefs over challenges helped the volunteers working in this new environment. In addition, the~~

566

~~nurse le~~

~~aders sent out monthly emails with updates on what teaching was happening in the~~

567

~~classroom and reminded clinical mentors which nurses were junior and needed more support, as~~

568

~~well as everyone’s English level (~~

~~Participant 1~~

~~. They also hosted monthly inform~~

~~)~~

~~al meetings~~

569

~~over dinner (~~

~~Participant 4~~

~~).~~

570

**Formatted:**

Font:

Not

Bold

**Formatted:**

Font:

Not

Bold,

Not

Italic

**Formatted:**

Font:

Not

Italic

**Formatted:**

Indent:

First

line:

",

0

Space

After:

0

pt

~~Three of the nurse leaders~~

~~cited~~

~~the most significant goal of the clinical mentor’s orientation was~~

571

~~to highlight the importance of cultural humility and fostering an environment of refle~~

~~ct~~

~~ivity. The~~

572

~~leadership team~~

~~was also cognizant that despite a pre~~

~~-~~

~~departure and on~~

~~-~~

~~site orientation, the~~

573

~~volunteers would also need time to experience and adjust to their new setting (~~

~~Participant 5~~

~~)~~

~~.~~

574

~~They encourage clinical mentors to first observe and learn the process, care, and cu~~

~~lture of~~

575

~~LFHC before offering suggestions for improvement (~~

~~Participant 3~~

~~)~~

~~.~~

576

~~Staff Responses to Mentorship~~

~~.~~

~~The nurse leaders reported that,~~

~~initially, at the bedside,~~

577

~~the Lao staff were anxious about having a mentor as they felt it singled them out and~~

~~were~~

578

~~worried it reflected on their poor performance (~~

~~Participant 1~~

~~)~~

~~. After a lot of explanation by the~~

579

~~leadership, the Lao staff began requesting shifts scheduled with a clinical mentor (~~

~~Participant 4~~

~~)~~

~~.~~

580

~~Thus, the nurse leaders stated that the clinical ment~~

~~orship at the bedside was an overall positive~~

581

~~experience for the Lao staff and was essential in their early stages of learning. They each~~

582

~~commented on how the Lao staff w~~

~~ere~~

~~welcoming to the clinical mentors. The Lao nurses would~~

583

~~invite their clinical ment~~

~~ors to dinners~~

~~and~~

~~weddings~~

~~,~~

~~and overall enjoyed their friendships with~~

584

~~their mentors.~~

585

~~Despite the generally positive experience with the mentorship, challenges remained. Verbal~~

586

~~and non~~

~~-~~

~~verbal shows of communication were a significant determinant~~

~~of the success of a~~

587

~~clinical mentor from the Lao staff’s perspective (~~

~~Participant 3~~

~~)~~

~~. All six nurse leaders highlighted~~

588

~~the staff’s sensitivity to their mentor’s tone and attitude. For example, in~~

~~the Lao~~

~~culture, it is~~

589

~~uncommon to get loud and frustrated~~

~~, so the less strict volunteers had more success in the chaos~~

590

~~of the acute care hospital (~~

~~Participant 4~~

~~)~~

~~. Fu~~

~~r~~

~~ther, if a volunteer was not motivated to mentor the~~

591

~~Lao nurses, it was palpable (~~

~~Participant 6~~

~~)~~

~~. Communication challenges, ho~~

~~w~~

~~ever, were secondary~~

592

~~to overcoming low health literacy in Lao (~~

~~Participant 1~~

~~)~~

~~. Medical terminology in the Lao~~

593

**Formatted:**

Font:

Not

Bold,

Not

Italic

**Formatted:**

Space

After:

0

pt

**Formatted:**

Font:

Not

Italic

**Formatted:**

Font:

Not

Bold

language, and the actual diseases here” (Participant 1). Continuous adaptation and openness to

618

feedback from Lao nurses ensured that curricula remained relevant, effective, and grounded in

619

loc

al context.

620

*Practicing Humility as an Ongoing Process*

621

Participants emphasized that cultural humility was not a single adjustment but a

622

continuous process of reflection and adaptation. One explained,

*“Cultural humility meant*

623

*reminding ourselves*

*to keep learning. Every time we thought we understood, something new*

624

*challenged our assumptions”*

Participant 2). Another reflected,

(

*“The most important part was*

625

*not assuming we knew better. We had to listen first, and then work together to find solutions*

*”*

626

Participant 4). Humility was therefore seen as a mindset that required sustained openness to

(

627

learning and adaptation in everyday interactions.

628

*~~Lao Cultural Practice~~*

*~~.~~*

~~All of the nursing leaders emphasized the importance of~~

629

~~respecting and valuing local c~~

~~ulture and healthcare practices in the development of the nurse~~

630

~~training program at LFHC. For example, in Laos, post~~

~~-~~

~~birth customs require that mothers sit by~~

631

~~the fire for a specific number of days after childbirth, and colostrum is considered harmful to t~~

~~he~~

632

~~infant and is not administered (~~

~~Participant 6~~

~~)~~

~~. Understanding these cultural practices was~~

633

~~essential when providing training for nurses in the neonatal department.~~

~~Additionally, t~~

~~raditional~~

634

~~medicine is highly regarded in Laos, and families often request~~

~~integrative practices. For~~

635

~~instance, the family of a toddler being treated for complex pneumonia requested to take him back~~

636

~~to the village for a goat sacrifice ceremony, informing the staff that they would return within~~

637

~~twenty~~

~~-~~

~~four hours. Despite this con~~

~~flicting with~~

~~the~~

~~current~~

~~understanding of~~

~~best practices, the~~

638

~~Lao staff respected the family’s wishes and sent the child home with oral antibiotics until his~~

639

~~return (~~

~~Participant 1~~

~~)~~

~~. Additionally, many people in Laos hold the belief that a deceased spirit~~

640

**Formatted:**

Font:

Not

Bold,

Italic

**Formatted:**

Space

After:

0

pt,

Line

spacing:

Double

**Formatted:**

Font:

Not

Bold,

Not

Italic

~~cannot be brought back to the home (Participant 3). As a result, when families are aware that a~~ ~~patient may die, they sometimes choose to take the child home to pass in accordance with their~~ ~~spiritual beliefs, a practice that can conflict with the cultural norms found in high-income~~ ~~countries (Participant 1).~~

*~~LFHC Centric Courses.~~* ~~Interviewees cited the unique culture, language, and~~ ~~epidemiology of the region when discussing the effectiveness of personalizing the didactic~~ ~~courses for LFHC. In one of the first years, LFHC welcomed a group of volunteers who traveled~~ ~~to Laos specifically to teach the Emergency Triage Assessment and Treatment (ETAT) course~~ ~~written by the WHO. However, the training proved to be ineffective. Various aspects of the~~ ~~material were not at the level of what the staff needed, the language was a barrier to~~ ~~understanding, and some material also suggested practices that conflicted with Lao cultural~~ ~~practice (Participant 1). Moving forward, the course had to be adapted by the nurse educator so~~ ~~that each of the foundation nurse training courses taught at LFHC was written specifically for the~~ ~~staff. Following each course's implementation, the curricula were augmented to increase the~~ ~~relevance and effectiveness for the Lao staff.~~

#### 5. Discussion

5.1 Key Findings

The LFHC Nursing Training Program led to a notable improvement in the Lao nurses’ clinical skillset, as consistently emphasized by both clinical mentors and nurse leaders. In addition to strengthening procedural competence, the program facilitated a shift toward more advanced forms of clinical reasoning and problem-solving. Participants described how nurses began to move beyond rote memorization toward applying knowledge in practice, asking questions, and critically engaging with patient care. These changes reflect a transition from taskbased nursing to a more holistic and critical thinking approach to clinical practice.

A central factor in this development was the adaptation of the curriculum to the Lao context. By eliminating materials designed for high-income countries and customizing training to reflect local epidemiology, cultural practices, and available resources, the LFHC team ensured that the curriculum was both accessible and relevant. Such contextualization is consistent with literature on global health education, which emphasizes that externally imported curricula, without adaptation, often fail to achieve meaningful practice change [20–22]. The WHO ETAT course, for example, was attempted at LFHC but was reported to be of limited value without curriculum adaptations due to its misalignment with Lao nursing practice and cultural norms.

**Formatted:**

Indent:

First

line:

0

"

**Formatted:**

Indent:

First

line:

0.5

",

Space

After:

0

pt,

Line

spacing:

Double

The program’s combined strategy of structured didactic sessions and hands-on clinical mentorship was another key contributor to success. Bedside mentorship created a feedback loop in which nurses could immediately apply knowledge, receive guidance, and reflect on practice. Nurse leaders noted that mentorship also facilitated psychosocial support, helping Lao nurses develop confidence and autonomy in their roles. Importantly, the clinical mentorship model was not static. Over time, it shifted from broad support of all staff to more targeted mentorship for junior nurses, allowing for efficient use of mentor expertise.

A further strength of the LFHC program was its investment in sustainability through leadership development. The modified train-the-trainer approach was pivotal in building longterm capacity. By the second year, a Lao nurse was identified as a future educator, and by 2019, this nurse was independently teaching the core curriculum. This not only ensured program continuity but also signaled a critical shift in power and ownership from expatriate staff to Lao leadership. In qualitative accounts, this transition was described as a turning point that inspired other nurses to aspire to leadership roles. Such findings align with global evidence that local leadership and peer-to-peer teaching are essential for enduring health system strengthening [19].

Perhaps most importantly, the LFHC experience demonstrated that cultivating critical thinking among nurses is possible even in traditionally rote-based educational systems. Through compassionate teaching, interactive learning strategies, and culturally sensitive mentorship, Lao nurses developed the ability to analyze clinical situations, anticipate complications, and contribute actively to care planning. This represents a substantial departure from historical models of nursing education in LMICs and highlights the potential for global nursing initiatives to drive pedagogical change.

**Formatted:**

Line

spacing:

Double

Taken together, the findings of this evaluation underscore the importance of three interdependent elements in global nursing capacity building: contextual adaptation of curricula, integration of bedside mentorship, and investment in leadership through train-the-trainer strategies. The LFHC model demonstrates that when these elements are combined, nurse training can advance beyond technical skill acquisition to foster critical thinking, confidence, and sustainable leadership.

**5.2 Volunteer Perspectives and Bidirectional Learning**

A significant finding from this study was the difference in the mean scores reflecting volunteers’ knowledge of the role of Lao nursing before and after participation at LFHC (3.5 vs. 4.4). This increase demonstrates that international clinical mentors not only contributed to the strengthening of Lao nurses’ clinical skills but also deepened their own understanding of the cultural, clinical, and leadership roles of local nurses. This highlights the reciprocal nature of the program, in which mentorship fostered bidirectional learning and exchange. Importantly, it underscores the value of cultural humility in cross-cultural partnerships, as volunteers gained greater awareness of the Lao context and the complexities of nursing practice in a resource-variable setting. Such findings reinforce the importance of designing global mentorship initiatives as bidirectional learning opportunities that promote both local capacity building and the professional development of international collaborators.

**Formatted:**

Indent:

First

line:

0

"

~~The LFHC Nursing Training Program led to an overall improvement in the Lao nurses’ clinical~~ ~~skillset from the perspective of the clinical mentors and nurse leaders. Customizing the LFHC~~ ~~nurse training curriculum based on the Lao nurses' learning style was an essential part of the~~ ~~training process. The LFHC training program's use of didactic training and bedside clinical~~ ~~mentorship helped bridge the Lao nurses’ knowledge-to-practice gaps. LFHC also implemented~~ ~~a modified train-the-trainer approach, which has been widely utilized in sustainable capacity~~ ~~building [19]. The Lao nurse educator was identified amongst the nurses in the second year of~~ ~~training, and in 2019, they were teaching the curriculum independently with minimal assistance.~~ ~~Additionally, the experience of the train-the-trainer model highly contributed to promoting~~ ~~nursing in leadership at LFHC.~~

**5.3 Implications for Global Nursing Education**

The evaluation of the LFHC Nursing Training Program provides important lessons for nursing education in resource-variable settings. First, the findings reinforce that standardized, externally developed training programs may not achieve their intended outcomes without meaningful cultural and contextual adaptation. Efforts to implement global curricula such as

WHO’s ETAT course highlight this limitation, as the program was perceived as misaligned with local needs and practices. In contrast, the LFHC model demonstrated that tailoring content to reflect local learning styles, language, and health system realities can lead to measurable gains in both clinical skills and critical thinking.

Second, the integration of bedside mentorship alongside didactic learning proved critical. This aligns with growing evidence that experiential learning models are particularly effective for bridging knowledge-to-practice gaps in nursing. Mentorship not only improved technical competencies but also fostered professional identity and autonomy, qualities that are central to the advancement of nursing as a profession globally. For nurses in Laos, who traditionally trained in hierarchical and didactic systems, the opportunity to engage in dialogue, ask questions, and apply critical reasoning marked a transformative educational shift.

Finally, the study underscores the importance of embedding cultural humility into global nursing education. Expatriate mentors and educators at LFHC recognized that success depended on listening to Lao nurses’ perspectives, respecting local practices, and adapting pedagogical approaches accordingly. This emphasis on cultural humility rather than cultural competence promoted a spirit of mutual learning and helped establish trust, which was repeatedly highlighted in interviews as central to the program’s success.

**5.4 Sustainability and Leadership Development**

A key contribution of the LFHC program was its explicit focus on sustainability. Too often, nursing education initiatives are limited by dependence on external trainers or short-term funding. In contrast, LFHC prioritized developing local leadership capacity through a modified train-the-trainer approach. By the second year of the program, a Lao nurse was formally identified as an educator, and within three years, they assumed independent responsibility for delivering the curriculum. This transition not only secured the program’s continuity but also elevated the professional role of nurses within the hospital.

Leadership development extended beyond the formal educator role. Qualitative findings revealed that exposure to mentorship and responsibility inspired many Lao nurses to envision themselves as leaders in their units. Several participants reflected that the program encouraged them to mentor junior colleagues, set standards for practice, and advocate for patient care improvements. These findings echo global nursing literature that emphasizes the link between local leadership and sustainable improvements in care quality.

The emphasis on sustainability also offers insights for broader health systems. By empowering Lao educators and fostering critical thinking among staff, the LFHC program contributed to resilience during crises, including the COVID-19 pandemic, when international travel restrictions severely limited expatriate support. In this way, the program illustrates how investments in LMIC nursing leadership can buffer against system shocks and promote longterm independence from external aid.

**5.5 Broader Global Health Implications**

Beyond the context of LFHC, this evaluation contributes to the discourse on how global partnerships can advance health equity. It illustrates the value of bi-directional learning, where expatriate mentors adapted their teaching to local contexts while simultaneously learning from Lao nurses about culturally grounded care practices. Such partnerships advance not only technical skills but also relational capacity, which is critical for sustainable health system strengthening.

Furthermore, the LFHC experience provides a replicable model for other low- and middle-income countries seeking to strengthen pediatric nursing education. The combination of contextualized curricula, mentorship, and leadership development could be adapted for various clinical areas, from emergency and critical care to maternal and newborn health.

~~The study findings highlighted ways in which standardized training programs' lack of~~ ~~cultural relevance can limit their effectiveness in improving clinical practice [20-22]. For~~ ~~instance, there were attempts to implement standardized curricula designed for resource-limited~~ ~~countries, such as the World Health Organization's ETAT course, but they had limited~~ ~~application to the Lao cultural practices, available healthcare resources, and regional~~ ~~epidemiology (Participant 1).~~

**Formatted:**

Space

After:

0

pt

**Formatted:**

Space

After:

0

pt,

Line

spacing:

Double

##### **5.6 ~~1~~ Strengths and Limitations of the Study**

The strengths of this project include strong engagement and participation from nursing leadership at LFHC. The clinical mentorship survey achieved a response rate of 45 percent, and six of the seven former nurse leaders from LFHC participated in semi-structured interviews, ensuring a broad representation of leadership perspectives. The use of a mixed-methods approach further strengthened the evaluation by helping to mitigate potential deficiencies and biases associated with reliance on a single method [23].

This study also has several limitations. A primary limitation was recall bias, as participants were asked to reflect on past work experiences [24]. Social desirability bias may have further influenced responses, particularly in relation to sensitive topics such as leadership and training effectiveness. The COVID-19 pandemic imposed additional challenges, including the absence of a standardized, validated survey tool, reliance on online technologies with varying levels of internet connectivity, and the inability to conduct in-person data collection. Lastly, due to travel restrictions during the pandemic, feedback from Lao staff could not be incorporated, limiting the inclusion of their perspectives, which are essential for a comprehensive evaluation of the LFHC Pediatric Nursing Training Program. Despite these limitations, the study provides critical insights into the development, implementation, and sustainability of pediatric nursing education and mentorship at LFHC, offering lessons that may be transferable to education program development in similar resource-variable settings.

~~The strengths of this project include strong engagement and participation from nursing~~ ~~leadership at LFHC. The clinical mentorship survey received a solid response rate of 45%, and~~ ~~six of the seven former nurse leaders from LFHC participated in the semi-structured interviews.~~ ~~Additionally, the use of a mixed-methods approach helped mitigate potential deficiencies and~~ ~~biases associated with relying on a single method [23]. A primary limitation of this project was~~ ~~recall bias, as participants were asked to reflect on past work experiences [24]. Furthermore, due~~ ~~to the COVID-19 pandemic and the absence of a standardized, validated survey tool for this~~ ~~project, the strength of the data is limited. Additionally, the reliance on online technologies~~ ~~introduced additional challenges, including navigating varying technologies and reliance on~~ ~~internet connectivity. Lastly, due to travel restrictions during the COVID-19 pandemic, feedback~~ ~~from the Lao staff was not included, and their perspective is crucial for a comprehensive~~ ~~evaluation of the LFHC Pediatric Nursing Training Program.~~

##### **5.7~~2~~ Recommendations for Further Research**

This study provides evidence for new approaches to building and

evaluating sustainable

816

and culturally relevant nursing training programs in resource

-

variable settings. The lessons

817

learned from this project can inform future capacity

-

building initiatives by highlighting

818

techniques to identify potential barriers early an

d equipping program developers with the tools

819

necessary to ensure program success.

820

Future research is needed in several areas. First, further studies should evaluate the long

-

821

term outcomes of train

-

the

-

trainer models, particularly regarding how they influe

nce nurse

822

retention, leadership development, and patient outcomes. Second, research should focus on

823

refining methods for integrating

cultural humility

, which must be a central component of any

824

cross

-

cultural partnership, into training curricula. This inclu

des evaluating how cultural humility

825

practices affect learner engagement, mutual respect, and the sustainability of acquired skills.

826

Third, there is a need for comparative studies examining how different mentorship models

,

such

827

as in

-

person, virtual, or hy

brid

,

affect knowledge translation and practice change across resource

-

828

variable contexts. Finally, involving

nurses in LMIC’s

perspectives, which were limited in this

829

study due to COVID

-

19

restrictions, will be crucial for gaining a more comprehensive

830

unde

rstanding of training

needs,

effectiveness

,

and sustainability.

831

~~This study provides evidence for new efforts~~

~~in~~

~~building and evaluating sustainable and~~

832

~~culturally relevant nursing training programs in resource~~

~~-~~

~~variable settings~~

~~. The lessons learned~~

833

~~from th~~

~~is project can aid future capacity~~

~~-~~

~~building efforts with techniques to identify potential~~

834

~~barriers and equip program developers with skills~~

~~for~~

~~the program’s success. Future research is~~

835

~~needed related to capacity building, especially in the field of traini~~

~~ng program development and~~

836

~~implementation.~~

837

838

**Formatted:**

Indent:

First

line:

",

0.56

Space

After:

0

pt,

Line

spacing:

Double

**Formatted:**

Font:

Not

Bold

~~settings, it is crucial to ensure their cultural humility through pre-departure preparation, continual~~ ~~mentoring, and feedback to increase their efficacy.~~

## Acknowledgements

The authors would like to express their sincere gratitude to the dedicated nursing staff at Lao Friends Hospital for Children (LFHC) for their unwavering commitment to skill development and their pioneering work in specialized pediatric care. We are deeply appreciative of our continued collaboration. We are also thank the former nursing leaders and volunteers who contributed to this evaluation, ~~—~~Their colletive contributions continue to strengthen nursing practice and leadership at LFHC, with Lao nursing stsff now taking the lead in fostering the gowth of the furture of pediatric nursing in Laos~~your enduring impact is felt throughout the~~ ~~hospital every da~~. ~~y. It has been an honor to work alongside each of you in service of our~~

~~pediatric community.~~

**REFERENCES**

1. United Nations Children's Fund (UNICEF). Under five mortality [Internet]. 2018 [cited 2025

Jun 2]. Available from: https://data.unicef.org/topic/child-survival/under-five-mortality/

2. Liu L, Oza S, Hogan D, Perin J, Rudan I, Lawn J, et al. Global, regional, and national causes of child mortality in 2000–13, with projections to inform post-2015 priorities: An updated systematic analysis. Lancet. 2015;385(9966):430–40.
3. Rutherford M, Dockerty J, Jasseh M, Howie S, Herbison P, Jeffries D, et al. Access to health care and mortality of children under 5 years of age in the Gambia: A case-control study. Bull World Health Organ. 2009;87(3):216–25.
4. Kissoon N, Burns J. Who should get pediatric intensive care when not all can? A call for international guidelines on allocation of pediatric intensive care resources. Pediatr Crit Care Med. 2014;15(1):82–3.
5. World Health Organization. WHO global disability action plan 2014–2021: Better health for all people with disability [Internet]. Geneva: WHO; [cited 2025 Jun 2]. Available from: https://www.who.int/disabilities/actionplan/en/
6. Wong FKY, Liu H, Wang H, Anderson D, Seib C, Molasiotis A. Global nursing issues and development: An analysis of World Health Organization documents. J Nurs Scholarsh.

2015;47(6):574–83.

7. Morin KH. Evolving global education standards for nurses and midwives. MCN Am J Matern

Child Nurs. 2012;37(6):360–6.

8. Slusher TM, Kiragu AW, Day LT, Bjorklund AR, Shirk A, Johannsen C, et al. Pediatric critical care in resource-limited settings—Overview and lessons learned. Front Pediatr.

2018;6:49.

9. Pacific Bridge Medical. Healthcare in Laos [Internet]. 2017 Jul 11 [cited 2025 Jun 2].

Available from: https://www.pacificbridgemedical.com/publication/healthcare-in-laos/

10. Barron A, Moran J, Nina S, Harlow J, Gyawali M, Hossain F, et al. Building specialized nursing practice capacity in Bangladesh: An educational program to prepare nurses to care for oncology and bone marrow transplant patients in Dhaka, Bangladesh. J Glob Oncol.

2018;4(4):1–6.

11. Halabi JO, Abdalrahim MS, Persson GL, Hedemalm A, Lepp M. The development of a preceptor training program on clinical nursing education in Jordan in collaboration with Sweden.

J Contin Educ Nurs. 2012;43(3):135–44.

12. Kumar R, Canarie M. Developing pediatric critical care in Kenya. Pediatr Crit Care Med.

2019;20(12):e538.

13. McConnell KA, Krisher LK, Lenssen M, Bunik M, Bunge Montes S, Domek GJ. Telehealth to expand community health nurse education in rural Guatemala: A pilot feasibility and acceptability evaluation. Front Public Health. 2017;5:60.
14. Harris PA, Taylor R, Thielke R, Payne J, Gonzalez N, Conde JG. Research electronic data capture (REDCap) – A metadata-driven methodology and workflow process for providing translational research informatics support. J Biomed Inform. 2009;42(2):377–81.
15. Harris PA, Taylor R, Minor BL, Elliott V, Fernandez M, O’Neal L, et al. The REDCap consortium: Building an international community of software partners. J Biomed Inform.

2019;103208.

16. Leininger M. Leininger's theory of nursing: Cultural care diversity and universality. Nurs Sci Q. 1988;1(4):152–60.
17. Sullivan B, Bettger J. Community-informed health promotion to improve health behaviors in Honduras. J Transcult Nurs. 2018;29(1):14–20.
18. Trint Ltd. Trint Platform [Internet]. [cited 2020]. Available from: https://www.trint.com/
19. Bell S, Bam V, Acheampong E. Developments in emergency nursing education in Ghana.

Emerg Nurse. 2015;23(8):18–21.

20. Canarie M, Shenoi A. Teaching the principles of pediatric critical care to non-intensivists in resource limited settings: Challenges and opportunities. Front Pediatr. 2018;6:44.
21. Ralston M, Day L, Slusher T, Musa N, Doss H. Global paediatric advanced life support:

Improving child survival in limited-resource settings. Lancet. 2013;381(9862):256–65.

22. Shilkofski N, Hunt E. Identification of barriers to pediatric care in limited-resource settings:

A simulation study. Pediatrics. 2015;136(6):e1569–75.

23. Thurmond V. The point of triangulation. J Nurs Scholarsh. 2001;33(3):253–8.
24. Melnyk BM, Fineout-Overholt E. Evidence-based practice in nursing & healthcare: A guide to best practice. 4th ed. Philadelphia: Wolters Kluwer; 2019.

Response to Reviewers

# August 20^th^, 2025

Dear PLOS ONE Academic Editor and Reviewers,

We are grateful for the time and thoughtful feedback provided during the review of our manuscript. We have carefully revised the text to address all comments and believe these revisions have strengthened the clarity, rigor, and contribution of our work. Below we provide a point-by-point response, with reviewer comments in italics followed by our responses regarding the following manuscript:

*Pediatric Capacity Building in Northern Laos: An Evaluation of the Pediatric Nursing Training Program at Lao Friends Hospital for Children*

# Methods

## 1. Study setting and design

*Comment: Please include a description of the LFHC setting (level of hospital, services rendered, staff strength, staff caliber, and possibly child mortality figures).*

**Response:** We have added a description of LFHC as a tertiary pediatric referral hospital in Luang Prabang, Laos. The revised text now includes information on the types of services provided, the scope of staff roles, and its role in serving a vulnerable pediatric population.

## 2. Sample selection and recruitment

*Comment: Certain sentences belong in other sections (voluntary participation → ethics, pilot test → validity, interview details → data collection).*

**Response:** We have reorganized these elements into appropriate sections. Voluntary participation and confidentiality are now described under Ethical Considerations. Details of the pilot survey are presented under Tools, and interview procedures are included in the Data Collection subsection.

## 3. Data analysis

*Comment: More detail is needed on quantitative (means, SDs) and qualitative (grounded theory, coding, theme identification, anonymization) analysis.*

**Response:** We have expanded our description of both quantitative and qualitative data analysis. For the quantitative data, we clarify that descriptive statistics (means, SDs, and percentages) were calculated using Microsoft Excel. For qualitative data, we elaborate on the grounded theory approach, including the coding process, identification of themes, triangulation by multiple reviewers, and anonymization of transcripts.

# Ethical Considerations

*Comment: Clarify “non-research” IRB status, type of approval from LFHC, and include details on participation, right to withdraw, risks/benefits, compensation, confidentiality, and data protection.*

**Response:** We have clarified that the Vanderbilt University IRB reviewed this project and granted it “exempt” status, which is a formal university designation for program evaluations that do not involve biomedical interventions or place participants at risk. This status indicates that the project does not require ongoing IRB monitoring but is nonetheless subject to university oversight and human subjects’ protection standards. In addition, the LFHC Research Committee independently reviewed and approved the study protocol. The revised section now explicitly outlines voluntary participation, right to withdraw, absence of financial incentives, anonymity, confidentiality safeguards, and secure storage of recordings.

# Results

## 1. Likert scale clarification

*Comment: Clarify if Likert ratings were by direct observation, assessment, or recall.* **Response:** We now specify that Likert ratings were completed by expatriate clinical mentors based on their direct observation of nurses’ skills during training and practice, with some recall bias acknowledged.

## 2. Mean score changes (Table 2)

*Comment: Explain improvements and declines in specific skills; discuss significance of change from 3.5 → 4.4.*

**Response:** We have expanded the Results and Discussion sections to reflect that improvements in mean scores demonstrate not only increased clinical skills but also enhanced critical thinking among Lao nurses. We also added a discussion of the significant increase in mentors’ selfreported knowledge of the Lao nursing role (3.5 → 4.4).

## 3. Potential biases

*Comment: Expatriate mentors may have biased program ratings.*

**Response:** We acknowledge this limitation explicitly in the Discussion, noting that expatriate mentors both delivered and evaluated the program, which may have influenced objectivity. To mitigate this, we used multiple data sources (surveys, interviews, curriculum review) to triangulate findings.

## 4. Self-reflection section

*Comment: Provide graphical presentation, analyze influence of changes, link to skills, discuss social desirability bias.*

**Response:** We appreciate this suggestion. While we considered adding a graphical presentation, we determined that the small sample size and exploratory nature of the data limited the utility of additional figures. Instead, we have clarified in the manuscript that the changes in self-reflection responses most likely reflect an increased cultural understanding of Lao nursing roles, though these findings cannot be directly linked to skill-based outcomes presented in Table 2. We therefore avoid overinterpretation of the results. To address the reviewer’s concern about social desirability bias, we have expanded the Discussion to note this as a potential limitation and outlined the measures taken to reduce its impact, including the use of anonymized surveys, voluntary participation, and assurances of confidentiality.

## 5. Semi-structured interviews

*Comment: Present findings in qualitative structure with quotes, not narration.*

**Response:** The section has been significantly revised to highlight key themes supported by participant quotes, presenting findings in a qualitative structure to better reflect interview data.

# Discussion

## 1. Key findings

*Comment: Expand discussion of cultural immersion and link to transcultural nursing theory.* **Response:** We added content linking cultural immersion of mentors to the principles of transcultural nursing theory, emphasizing how cultural humility was integral to program success.

**2. Significant difference in mean scores (3.5 vs. 4.4)** *Comment: Discuss this difference.*

**Response:** Addressed as noted above, highlighting the significance of this increase in enhancing mentors’ understanding of the Lao nursing role.

## 3. Limitations and biases

*Comment: Address recall bias, expatriate role, technology issues, lack of Lao staff voice, etc.* **Response:** These have all been integrated into the expanded Limitations section.

## 4. Further research

*Comment: Suggest areas for future study.*

**Response:** A new subsection recommends further research on culturally relevant, sustainable training models in resource-variable settings, with explicit inclusion of cultural humility in crosscultural partnerships.

We sincerely thank the reviewers and editor for their valuable comments, which have significantly improved the quality of our manuscript. We hope the revisions and clarifications provided meet expectations and respectfully resubmit our manuscript for your consideration.

Sincerely,

Rebecca Silvers DNP, APN, CPNP-AC, CCRN, RNFA

On behalf of all co-authors

1. Institute of Global Health Sciences 550 16th Street SF CA, USA
2. Lao Friends Hospital for Children, Nasang Road, Ban Phoumork, Luang Prabang, Lao PDR
3. Vanderbilt School of Nursing, 461 21^st^ Ave South, Nashville, TN USA
4. Institute of Global Health Sciences 550 16th Street SF CA, USA
5. Lao Friends Hospital for Children, Nasang Road, Ban Phoumork, Luang Prabang, Lao PDR
6. Vanderbilt School of Nursing, 461 21^st^ Ave South, Nashville, TN USA
