# Supplementary material for: Pediatric Capacity Building in Northern Laos: An Evaluation of the Pediatric Nursing Training Program at Lao Friends Hospital for Children
Source: PLoS One. 2026 Jul 15;21(7):e0349226. doi: 10.1371/journal.pone.0349226 (PMC13372149; doi:10.1371/journal.pone.0349226)
Supplement: Attachment — Submitted filename: Reviewer comments_PLOS ONE_2025.docx [file pone.0349226.s003.docx]

Thank you for the opportunity to review this paper.

Title: Pediatric Capacity Building in Northern Laos: An Evaluation of the Pediatric Nursing Training Program at Lao Friends Hospital for Children

**Methods**

**Study setting and Design**

Comment:

1. Line 147-157: Generally, the study design is well-described. However, the authors did not include a description of the setting. A brief description of the LFHC would give readers an understanding of the hospital. Include the level (primary, secondary, tertiary) or if it is a referral hospital. Include services rendered, staff strength, calibre of staff and possibly child mortality figures

**Sample Selection and Recruitment**

**Comment:**

2. Line 166-179: The authors described how the participants will be selected and recruited. However, there are some statements in this section that may not be related. Authors should address these issues by creating appropriate headings to realign some statements
i. Line 170-171: “*Participation in the survey was voluntary, and respondents were not required to submit identifiable information”.* This phrase may be suitable at the ethics section.
ii. Line 171-172: “*The survey was administered in English”.*
iii. *Line 178: “The interviews were conducted via Zoom and were recorded with the participants' permission”.*
iv. *Lines 179: “Each interview was designed to last approximately one hour”.*

The above phrases seem more suitable for a data collection method.

3. Line 172-173: “*A pilot test was conducted to enhance the face and content validity of the survey before it was distributed”.* This statement seems more suitable under validity.

**Data Analysis**

**Comments:**

4. Lines 207-216: The authors failed to describe in detail how data analysis for the qualitative and quantitative data was done.
5. The analysis is deficient in how the means and standard deviations in Table 2 were achieved. This should be done adequately.
6. The authors should include in detail all the statistical methods used in the quantitative data analysis.
7. Authors should describe extensively the processes of qualitative data analysis under the grounded theory or any other literature being used, including coding, identification of themes and sub-themes, and how individual transcribed data were anonymized.

**Ethical Considerations**

**Comment:**

8. Lines 218-219: *“This study received ethical approval from the Institutional Review Board (IRB) at Vanderbilt 218 University under exempt (non-research) status (Protocol #202298).”*
9. The authors should explain what is meant by “non-research” status in this regard. This is a research project, and why should the ethics be non-research status?
10. What type of approval was given by the LFHC? Indicate
11. Include information on voluntary participation, right to withdraw, risks and benefits, compensation, confidentiality, anonymity, and privacy. Also, include information on assurance of non-disclosure of recorded information

**Results**

**Evaluation of the Content of the Training Program**

**Comments:**

12. Lines 279-291: Could the authors explain or clarify in the methods section (data analysis) how the Likert scale ratings were done? By direct observation, assessment or through recall of past experience (memory)?
13. The data showed a change in mean scores in Table 2, but the authors did not elaborate on why certain aspects of the skills (improved and decreased)
14. Could the authors elaborate whether there was a significant relationship between changes in skills as observed in Table 2 and other variables, such as demographic characteristics of participants, mentorship duration and possibly educational interventions? This will add clarity to findings
15. There could be issues of potential biases since the expatriate mentors were those who provided the ratings. How could they rate the program they were part of objectively? Could the authors clarify issues of biases.

**Participants’ Self-Reflection on Contributions to the Training Program**

16. Authors should show a graphical presentation of the items under this section for clarity.
17. Authors should show further analysis on what influenced the changes in mean score before and after volunteering at LFHC (3.5 vs 4.4)
18. Could the authors link their self-reflection contribution to the objective skills outline in table 2? This will show the impact of mentor on clinical outcomes
19. Could the authors reflect on the possibility of social desirability bias in self-reflection? They should take steps in the methods section to explain how this was mitigated or addressed

**LFHC Program Evaluation: Semi-Structured Interviews**

Comments:

20. Lines 318-328: Information here could be added to the table on demographics to free space. This information is demographic in nature
21. Lines 329-471: This should present data in a qualitative structure. The authors should revise this part to avoid narration and instead present the findings from the interview, which takes the form of qualitative research.

**Discussion**

22. The discussion should include the key findings of the study
23. The findings indicated that cultural immersion influenced mentors’ understanding of the program. But the authors did not discuss this relevant aspect to indicate its linkage with the transcultural nursing theory. This could improve the discussion section
24. There is a significant difference in the mean scores before volunteering to LFHC and at the end of participating in LFHC (3.5 vs 4.4). however, the authors did not discuss these significant differences. If done, it could improve the discussion section.

**General comments**

25. Could the authors create and describe a section or data collection method effectively? This will give clarity to the types of methods used in data collection in this study. The description of the methods section does not make this clear.
26. Under the data collection method section, authors should indicate the level at which saturation was attained and the number of interviews that were conducted
27. The authors should create sections for validity and reliability and trustworthiness. This will give clarity to the tool's validity and reliability for quantitative data and ensure rigor through ensuring qualitative trustworthiness.
28. Move all statements about ethics in the methods to the ethical consideration section.
29. Authors should create a “Study Participants” section and describe the participants vividly. This will give more clarity on who the participants were, why they were selected, and their role in the study.
