# Supplementary material for: Pediatric Capacity Building in Northern Laos: An Evaluation of the Pediatric Nursing Training Program at Lao Friends Hospital for Children
Source: PLoS One. 2026 Jul 15;21(7):e0349226. doi: 10.1371/journal.pone.0349226 (PMC13372149; doi:10.1371/journal.pone.0349226)
Supplement: Attachment — Submitted filename: Plos ONE-revised comments.docx [file pone.0349226.s005.docx]

Thank you for contacting me to revise the revised manuscript. The authors have effectively addressed my comments, and I am satisfied with their input.
