# Supplementary material for: Pediatric Capacity Building in Northern Laos: An Evaluation of the Pediatric Nursing Training Program at Lao Friends Hospital for Children
Source: PLoS One. 2026 Jul 15;21(7):e0349226. doi: 10.1371/journal.pone.0349226 (PMC13372149; doi:10.1371/journal.pone.0349226)
Supplement: Attachment — Submitted filename: PLOS ONE_Laos.docx [file pone.0349226.s008.docx]

Dear editor,

Thank you for the opportunity to review this manuscript again. At this point, I no longer have any further comments for the authors.

Thank you.
