# Supplementary material for: Pediatric Capacity Building in Northern Laos: An Evaluation of the Pediatric Nursing Training Program at Lao Friends Hospital for Children
Source: PLoS One. 2026 Jul 15;21(7):e0349226. doi: 10.1371/journal.pone.0349226 (PMC13372149; doi:10.1371/journal.pone.0349226)
Supplement: Peer review history

# Peer review: Decision Letter 0

**DOI:** 10.1371/journal.pone.0349226.r001

**Authors:** Alejandro Torrado Pacheco

8 Aug 2025

PONE-D-25-29935

Pediatric Capacity Building in Northern Laos: An Evaluation of the Pediatric Nursing Training Program at Lao Friends Hospital for Children

PLOS ONE

Dear Dr. Silvers,

Thank you for submitting your manuscript to PLOS ONE. After careful consideration, we feel that it has merit but does not fully meet PLOS ONE’s publication criteria as it currently stands. Therefore, we invite you to submit a revised version of the manuscript that addresses the points raised during the review process.

==============================

The manuscript has been evaluated by two reviewers, and their comments are available below.

The reviewers make several recommendations to improve the clarity and quality of the manuscript.

Could you please revise the manuscript to carefully address the concerns raised?

==============================

Please submit your revised manuscript by Sep 21 2025 11:59PM. If you will need more time than this to complete your revisions, please reply to this message or contact the journal office at plosone@plos.org. When you're ready to submit your revision, log on to https://www.editorialmanager.com/pone/ and select the 'Submissions Needing Revision' folder to locate your manuscript file.

Please include the following items when submitting your revised manuscript:

- A rebuttal letter that responds to each point raised by the academic editor and reviewer(s). You should upload this letter as a separate file labeled 'Response to Reviewers'.
- A marked-up copy of your manuscript that highlights changes made to the original version. You should upload this as a separate file labeled 'Revised Manuscript with Track Changes'.
- An unmarked version of your revised paper without tracked changes. You should upload this as a separate file labeled 'Manuscript'.

If you would like to make changes to your financial disclosure, please include your updated statement in your cover letter. Guidelines for resubmitting your figure files are available below the reviewer comments at the end of this letter.

If applicable, we recommend that you deposit your laboratory protocols in protocols.io to enhance the reproducibility of your results. Protocols.io assigns your protocol its own identifier (DOI) so that it can be cited independently in the future. For instructions see: https://journals.plos.org/plosone/s/submission-guidelines#loc-laboratory-protocols. Additionally, PLOS ONE offers an option for publishing peer-reviewed Lab Protocol articles, which describe protocols hosted on protocols.io. Read more information on sharing protocols at https://plos.org/protocols?utm_medium=editorial-email&utm_source=authorletters&utm_campaign=protocols.

We look forward to receiving your revised manuscript.

Kind regards,

Alejandro Torrado Pacheco, PhD

Staff Editor

PLOS ONE

Journal Requirements:

When submitting your revision, we need you to address these additional requirements.

1. Please ensure that your manuscript meets PLOS ONE's style requirements, including those for file naming. The PLOS ONE style templates can be found at

https://journals.plos.org/plosone/s/file?id=wjVg/PLOSOne_formatting_sample_main_body.pdf and

https://journals.plos.org/plosone/s/file?id=ba62/PLOSOne_formatting_sample_title_authors_affiliations.pdf.

2. We note that your Data Availability Statement is currently as follows: [All relevant data are within the manuscript and its Supporting Information files.]

Please confirm at this time whether or not your submission contains all raw data required to replicate the results of your study. Authors must share the “minimal data set” for their submission. PLOS defines the minimal data set to consist of the data required to replicate all study findings reported in the article, as well as related metadata and methods (https://journals.plos.org/plosone/s/data-availability#loc-minimal-data-set-definition).

For example, authors should submit the following data:

- The values behind the means, standard deviations and other measures reported;

- The values used to build graphs;

- The points extracted from images for analysis.

Authors do not need to submit their entire data set if only a portion of the data was used in the reported study.

If your submission does not contain these data, please either upload them as Supporting Information files or deposit them to a stable, public repository and provide us with the relevant URLs, DOIs, or accession numbers. For a list of recommended repositories, please see https://journals.plos.org/plosone/s/recommended-repositories.

If there are ethical or legal restrictions on sharing a de-identified data set, please explain them in detail (e.g., data contain potentially sensitive information, data are owned by a third-party organization, etc.) and who has imposed them (e.g., an ethics committee). Please also provide contact information for a data access committee, ethics committee, or other institutional body to which data requests may be sent. If data are owned by a third party, please indicate how others may request data access.

3. Please include a caption for figure 2.

4. Please upload a copy of Figure 2, to which you refer in your text on page 10. If the figure is no longer to be included as part of the submission please remove all reference to it within the text

5. If the reviewer comments include a recommendation to cite specific previously published works, please review and evaluate these publications to determine whether they are relevant and should be cited. There is no requirement to cite these works unless the editor has indicated otherwise.

[Note: HTML markup is below. Please do not edit.]

Reviewers' comments:

Reviewer's Responses to Questions

**Comments to the Author**

1. Is the manuscript technically sound, and do the data support the conclusions?

The manuscript must describe a technically sound piece of scientific research with data that supports the conclusions. Experiments must have been conducted rigorously, with appropriate controls, replication, and sample sizes. The conclusions must be drawn appropriately based on the data presented.

Reviewer #1: Yes

Reviewer #2: Partly

**********

2. Has the statistical analysis been performed appropriately and rigorously?

Reviewer #1: Yes

Reviewer #2: No

**********

3. Have the authors made all data underlying the findings in their manuscript fully available?

The PLOS Data policy requires authors to make all data underlying the findings described in their manuscript fully available without restriction, with rare exception (please refer to the Data Availability Statement in the manuscript PDF file). The data should be provided as part of the manuscript or its supporting information, or deposited to a public repository. For example, in addition to summary statistics, the data points behind means, medians and variance measures should be available. If there are restrictions on publicly sharing data—e.g. participant privacy or use of data from a third party—those must be specified.

Reviewer #1: Yes

Reviewer #2: No

**********

4. Is the manuscript presented in an intelligible fashion and written in standard English?

PLOS ONE does not copyedit accepted manuscripts, so the language in submitted articles must be clear, correct, and unambiguous. Any typographical or grammatical errors should be corrected at revision, so please note any specific errors here.

Reviewer #1: Yes

Reviewer #2: Yes

**********

5. Review Comments to the Author

Please use the space provided to explain your answers to the questions above. You may also include additional comments for the author, including concerns about dual publication, research ethics, or publication ethics. (Please upload your review as an attachment if it exceeds 20,000 characters)

Reviewer #1: The study is extremely interesting and provides evidence of how well-trained nursing staff can make a difference. However, I think that the complexity of the programme, which is well described in narrative form, could benefit from a graphic outline to help the reader understand “who” did “what”, “when”, with what “objective” and with what results. The qualitative analysis certainly offers interesting data, but on its own, in my opinion, it does not allow us to grasp the enormous amount of work carried out by this working group.

Reviewer #2: Thank you for the opportunity to review this paper.

Title: Pediatric Capacity Building in Northern Laos: An Evaluation of the Pediatric Nursing Training Program at Lao Friends Hospital for Children

Methods

Study setting and Design

Comment:

1. Line 147-157: Generally, the study design is well-described. However, the authors did not include a description of the setting. A brief description of the LFHC would give readers an understanding of the hospital. Include the level (primary, secondary, tertiary) or if it is a referral hospital. Include services rendered, staff strength, calibre of staff and possibly child mortality figures

Sample Selection and Recruitment

Comment:

2. Line 166-179: The authors described how the participants will be selected and recruited. However, there are some statements in this section that may not be related. Authors should address these issues by creating appropriate headings to realign some statements

i. Line 170-171: “Participation in the survey was voluntary, and respondents were not required to submit identifiable information”. This phrase may be suitable at the ethics section.

ii. Line 171-172: “The survey was administered in English”.

iii. Line 178: “The interviews were conducted via Zoom and were recorded with the participants' permission”.

iv. Lines 179: “Each interview was designed to last approximately one hour”.

The above phrases seem more suitable for a data collection method.

3. Line 172-173: “A pilot test was conducted to enhance the face and content validity of the survey before it was distributed”. This statement seems more suitable under validity.

Data Analysis

Comments:

4. Lines 207-216: The authors failed to describe in detail how data analysis for the qualitative and quantitative data was done.

5. The analysis is deficient in how the means and standard deviations in Table 2 were achieved. This should be done adequately.

6. The authors should include in detail all the statistical methods used in the quantitative data analysis.

7. Authors should describe extensively the processes of qualitative data analysis under the grounded theory or any other literature being used, including coding, identification of themes and sub-themes, and how individual transcribed data were anonymized.

Ethical Considerations

Comment:

8. Lines 218-219: “This study received ethical approval from the Institutional Review Board (IRB) at Vanderbilt 218 University under exempt (non-research) status (Protocol #202298).”

9. The authors should explain what is meant by “non-research” status in this regard. This is a research project, and why should the ethics be non-research status?

10. What type of approval was given by the LFHC? Indicate

11. Include information on voluntary participation, right to withdraw, risks and benefits, compensation, confidentiality, anonymity, and privacy. Also, include information on assurance of non-disclosure of recorded information

Results

Evaluation of the Content of the Training Program

Comments:

12. Lines 279-291: Could the authors explain or clarify in the methods section (data analysis) how the Likert scale ratings were done? By direct observation, assessment or through recall of past experience (memory)?

13. The data showed a change in mean scores in Table 2, but the authors did not elaborate on why certain aspects of the skills (improved and decreased)

14. Could the authors elaborate whether there was a significant relationship between changes in skills as observed in Table 2 and other variables, such as demographic characteristics of participants, mentorship duration and possibly educational interventions? This will add clarity to findings

15. There could be issues of potential biases since the expatriate mentors were those who provided the ratings. How could they rate the program they were part of objectively? Could the authors clarify issues of biases.

Participants’ Self-Reflection on Contributions to the Training Program

16. Authors should show a graphical presentation of the items under this section for clarity.

17. Authors should show further analysis on what influenced the changes in mean score before and after volunteering at LFHC (3.5 vs 4.4)

18. Could the authors link their self-reflection contribution to the objective skills outline in table 2? This will show the impact of mentor on clinical outcomes

19. Could the authors reflect on the possibility of social desirability bias in self-reflection? They should take steps in the methods section to explain how this was mitigated or addressed

LFHC Program Evaluation: Semi-Structured Interviews

Comments:

20. Lines 318-328: Information here could be added to the table on demographics to free space. This information is demographic in nature

21. Lines 329-471: This should present data in a qualitative structure. The authors should revise this part to avoid narration and instead present the findings from the interview, which takes the form of qualitative research.

Discussion

22. The discussion should include the key findings of the study

23. The findings indicated that cultural immersion influenced mentors’ understanding of the program. But the authors did not discuss this relevant aspect to indicate its linkage with the transcultural nursing theory. This could improve the discussion section

24. There is a significant difference in the mean scores before volunteering to LFHC and at the end of participating in LFHC (3.5 vs 4.4). however, the authors did not discuss these significant differences. If done, it could improve the discussion section.

General comments

25. Could the authors create and describe a section or data collection method effectively? This will give clarity to the types of methods used in data collection in this study. The description of the methods section does not make this clear.

26. Under the data collection method section, authors should indicate the level at which saturation was attained and the number of interviews that were conducted

27. The authors should create sections for validity and reliability and trustworthiness. This will give clarity to the tool's validity and reliability for quantitative data and ensure rigor through ensuring qualitative trustworthiness.

28. Move all statements about ethics in the methods to the ethical consideration section.

29. Authors should create a “Study Participants” section and describe the participants vividly. This will give more clarity on who the participants were, why they were selected, and their role in the study.

**********

6. PLOS authors have the option to publish the peer review history of their article (what does this mean?). If published, this will include your full peer review and any attached files.

If you choose “no”, your identity will remain anonymous but your review may still be made public.

**Do you want your identity to be public for this peer review?**  For information about this choice, including consent withdrawal, please see our Privacy Policy.

Reviewer #1: No

Reviewer #2: **Yes:**  Solomon Mohammed Salia

**********

[NOTE: If reviewer comments were submitted as an attachment file, they will be attached to this email and accessible via the submission site. Please log into your account, locate the manuscript record, and check for the action link "View Attachments". If this link does not appear, there are no attachment files.]

While revising your submission, please upload your figure files to the Preflight Analysis and Conversion Engine (PACE) digital diagnostic tool, https://pacev2.apexcovantage.com/. PACE helps ensure that figures meet PLOS requirements. To use PACE, you must first register as a user. Registration is free. Then, login and navigate to the UPLOAD tab, where you will find detailed instructions on how to use the tool. If you encounter any issues or have any questions when using PACE, please email PLOS at figures@plos.org. Please note that Supporting Information files do not need this step.

Attachment Submitted filename: Reviewer comments_PLOS ONE_2025.docx

# Author comment: Author response to Decision Letter 1

**DOI:** 10.1371/journal.pone.0349226.r002

21 Aug 2025

August 20th, 2025

Dear PLOS ONE Academic Editor and Reviewers,

We are grateful for the time and thoughtful feedback provided during the review of our manuscript. We have carefully revised the text to address all comments and believe these revisions have strengthened the clarity, rigor, and contribution of our work. Below we provide a point-by-point response, with reviewer comments in italics followed by our responses regarding the following manuscript:

Pediatric Capacity Building in Northern Laos: An Evaluation of the Pediatric Nursing Training Program at Lao Friends Hospital for Children

Methods

1. Study setting and design

Comment: Please include a description of the LFHC setting (level of hospital, services rendered, staff strength, staff caliber, and possibly child mortality figures).

Response: We have added a description of LFHC as a tertiary pediatric referral hospital in Luang Prabang, Laos. The revised text now includes information on the types of services provided, the scope of staff roles, and its role in serving a vulnerable pediatric population.

2. Sample selection and recruitment

Comment: Certain sentences belong in other sections (voluntary participation → ethics, pilot test → validity, interview details → data collection).

Response: We have reorganized these elements into appropriate sections. Voluntary participation and confidentiality are now described under Ethical Considerations. Details of the pilot survey are presented under Tools, and interview procedures are included in the Data Collection subsection.

3. Data analysis

Comment: More detail is needed on quantitative (means, SDs) and qualitative (grounded theory, coding, theme identification, anonymization) analysis.

Response: We have expanded our description of both quantitative and qualitative data analysis. For the quantitative data, we clarify that descriptive statistics (means, SDs, and percentages) were calculated using Microsoft Excel. For qualitative data, we elaborate on the grounded theory approach, including the coding process, identification of themes, triangulation by multiple reviewers, and anonymization of transcripts.

Ethical Considerations

Comment: Clarify “non-research” IRB status, type of approval from LFHC, and include details on participation, right to withdraw, risks/benefits, compensation, confidentiality, and data protection.

Response: We have clarified that the Vanderbilt University IRB reviewed this project and granted it “exempt” status, which is a formal university designation for program evaluations that do not involve biomedical interventions or place participants at risk. This status indicates that the project does not require ongoing IRB monitoring but is nonetheless subject to university oversight and human subjects’ protection standards. In addition, the LFHC Research Committee independently reviewed and approved the study protocol. The revised section now explicitly outlines voluntary participation, right to withdraw, absence of financial incentives, anonymity, confidentiality safeguards, and secure storage of recordings.

Results

1. Likert scale clarification

Comment: Clarify if Likert ratings were by direct observation, assessment, or recall.

Response: We now specify that Likert ratings were completed by expatriate clinical mentors based on their direct observation of nurses’ skills during training and practice, with some recall bias acknowledged.

2. Mean score changes (Table 2)

Comment: Explain improvements and declines in specific skills; discuss significance of change from 3.5 → 4.4.

Response: We have expanded the Results and Discussion sections to reflect that improvements in mean scores demonstrate not only increased clinical skills but also enhanced critical thinking among Lao nurses. We also added a discussion of the significant increase in mentors’ self-reported knowledge of the Lao nursing role (3.5 → 4.4).

3. Potential biases

Comment: Expatriate mentors may have biased program ratings.

Response: We acknowledge this limitation explicitly in the Discussion, noting that expatriate mentors both delivered and evaluated the program, which may have influenced objectivity. To mitigate this, we used multiple data sources (surveys, interviews, curriculum review) to triangulate findings.

4. Self-reflection section

Comment: Provide graphical presentation, analyze influence of changes, link to skills, discuss social desirability bias.

Response: We appreciate this suggestion. While we considered adding a graphical presentation, we determined that the small sample size and exploratory nature of the data limited the utility of additional figures. Instead, we have clarified in the manuscript that the changes in self-reflection responses most likely reflect an increased cultural understanding of Lao nursing roles, though these findings cannot be directly linked to skill-based outcomes presented in Table 2. We therefore avoid overinterpretation of the results. To address the reviewer’s concern about social desirability bias, we have expanded the Discussion to note this as a potential limitation and outlined the measures taken to reduce its impact, including the use of anonymized surveys, voluntary participation, and assurances of confidentiality.

5. Semi-structured interviews

Comment: Present findings in qualitative structure with quotes, not narration.

Response: The section has been significantly revised to highlight key themes supported by participant quotes, presenting findings in a qualitative structure to better reflect interview data.

Discussion

1. Key findings

Comment: Expand discussion of cultural immersion and link to transcultural nursing theory.

Response: We added content linking cultural immersion of mentors to the principles of transcultural nursing theory, emphasizing how cultural humility was integral to program success.

2. Significant difference in mean scores (3.5 vs. 4.4)

Comment: Discuss this difference.

Response: Addressed as noted above, highlighting the significance of this increase in enhancing mentors’ understanding of the Lao nursing role.

3. Limitations and biases

Comment: Address recall bias, expatriate role, technology issues, lack of Lao staff voice, etc.

Response: These have all been integrated into the expanded Limitations section.

4. Further research

Comment: Suggest areas for future study.

Response: A new subsection recommends further research on culturally relevant, sustainable training models in resource-variable settings, with explicit inclusion of cultural humility in cross-cultural partnerships.

We sincerely thank the reviewers and editor for their valuable comments, which have significantly improved the quality of our manuscript. We hope the revisions and clarifications provided meet expectations and respectfully resubmit our manuscript for your consideration.

Sincerely,

Rebecca Silvers DNP, APN, CPNP-AC, CCRN, RNFA

On behalf of all co-authors

Attachment Submitted filename: Response to Reviewers.pdf

# Peer review: Decision Letter 1

**DOI:** 10.1371/journal.pone.0349226.r003

**Authors:** Alejandro Torrado Pacheco, Douglas Aninng Opoku

16 Feb 2026

PONE-D-25-29935R1

Pediatric Capacity Building in Northern Laos: An Evaluation of the Pediatric Nursing Training Program at Lao Friends Hospital for Children

PLOS One

Dear Dr. Silvers,

Thank you for submitting your manuscript to PLOS ONE. After careful consideration, we feel that it has merit but does not fully meet PLOS ONE’s publication criteria as it currently stands. Therefore, we invite you to submit a revised version of the manuscript that addresses the points raised during the review process.

Please submit your revised manuscript by Apr 02 2026 11:59PM. If you will need more time than this to complete your revisions, please reply to this message or contact the journal office at plosone@plos.org. When you're ready to submit your revision, log on to https://www.editorialmanager.com/pone/ and select the 'Submissions Needing Revision' folder to locate your manuscript file.

Please include the following items when submitting your revised manuscript:

- A letter that responds to each point raised by the academic editor and reviewer(s). You should upload this letter as a separate file labeled 'Response to Reviewers'.
- A marked-up copy of your manuscript that highlights changes made to the original version. You should upload this as a separate file labeled 'Revised Manuscript with Track Changes'.
- An unmarked version of your revised paper without tracked changes. You should upload this as a separate file labeled 'Manuscript'.

-->If you would like to make changes to your financial disclosure, please include your updated statement in your cover letter. Guidelines for resubmitting your figure files are available below the reviewer comments at the end of this letter.

If applicable, we recommend that you deposit your laboratory protocols in protocols.io to enhance the reproducibility of your results. Protocols.io assigns your protocol its own identifier (DOI) so that it can be cited independently in the future. For instructions see: https://journals.plos.org/plosone/s/submission-guidelines#loc-laboratory-protocols. Additionally, PLOS ONE offers an option for publishing peer-reviewed Lab Protocol articles, which describe protocols hosted on protocols.io. Read more information on sharing protocols at https://plos.org/protocols?utm_medium=editorial-email&utm_source=authorletters&utm_campaign=protocols.

We look forward to receiving your revised manuscript.

Kind regards,

Douglas Aninng Opoku, MPH

Academic Editor

PLOS One

Journal Requirements:

If the reviewer comments include a recommendation to cite specific previously published works, please review and evaluate these publications to determine whether they are relevant and should be cited. There is no requirement to cite these works unless the editor has indicated otherwise.

Please review your reference list to ensure that it is complete and correct. If you have cited papers that have been retracted, please include the rationale for doing so in the manuscript text, or remove these references and replace them with relevant current references. Any changes to the reference list should be mentioned in the rebuttal letter that accompanies your revised manuscript. If you need to cite a retracted article, indicate the article’s retracted status in the References list and also include a citation and full reference for the retraction notice.

Additional Editor Comments:

You have done well by addressing the comments that were raised in the last review. However, there a a few issues including 'spellings' to address as pointed out by one of the reviewers. Work on them and your manuscript will be ready for publication in this journal.

[Note: HTML markup is below. Please do not edit.]

Reviewers' comments:

Reviewer's Responses to Questions

**Comments to the Author**

1. If the authors have adequately addressed your comments raised in a previous round of review and you feel that this manuscript is now acceptable for publication, you may indicate that here to bypass the “Comments to the Author” section, enter your conflict of interest statement in the “Confidential to Editor” section, and submit your "Accept" recommendation.

Reviewer #2: All comments have been addressed

Reviewer #3: (No Response)

**********

2. Is the manuscript technically sound, and do the data support the conclusions?

The manuscript must describe a technically sound piece of scientific research with data that supports the conclusions. Experiments must have been conducted rigorously, with appropriate controls, replication, and sample sizes. The conclusions must be drawn appropriately based on the data presented.

Reviewer #2: Yes

Reviewer #3: Yes

**********

3. Has the statistical analysis been performed appropriately and rigorously?

Reviewer #2: Yes

Reviewer #3: Yes

**********

4. Have the authors made all data underlying the findings in their manuscript fully available?

The PLOS Data policy requires authors to make all data underlying the findings described in their manuscript fully available without restriction, with rare exception (please refer to the Data Availability Statement in the manuscript PDF file). The data should be provided as part of the manuscript or its supporting information, or deposited to a public repository. For example, in addition to summary statistics, the data points behind means, medians and variance measures should be available. If there are restrictions on publicly sharing data—e.g. participant privacy or use of data from a third party—those must be specified.

Reviewer #2: Yes

Reviewer #3: Yes

**********

5. Is the manuscript presented in an intelligible fashion and written in standard English?

PLOS ONE does not copyedit accepted manuscripts, so the language in submitted articles must be clear, correct, and unambiguous. Any typographical or grammatical errors should be corrected at revision, so please note any specific errors here.

Reviewer #2: Yes

Reviewer #3: Yes

**********

6. Review Comments to the Author

Please use the space provided to explain your answers to the questions above. You may also include additional comments for the author, including concerns about dual publication, research ethics, or publication ethics. (Please upload your review as an attachment if it exceeds 20,000 characters)

Reviewer #2: (No Response)

Reviewer #3: The paper's technicality is very good and all the empirical information gathered very well support the findings and conclusions. Besides, the statistical analysis of the paper was systematically carried out and presented. The study made all suitable and relevant information available. More importantly, the paper was presented in standard English language. However, the authors should make clear the model of evaluation used to guide the presentation of the paper.

**********

7. PLOS authors have the option to publish the peer review history of their article (what does this mean?). If published, this will include your full peer review and any attached files.

If you choose “no”, your identity will remain anonymous but your review may still be made public.

**Do you want your identity to be public for this peer review?**  For information about this choice, including consent withdrawal, please see our Privacy Policy.

Reviewer #2: No

Reviewer #3: No

**********

[NOTE: If reviewer comments were submitted as an attachment file, they will be attached to this email and accessible via the submission site. Please log into your account, locate the manuscript record, and check for the action link "View Attachments". If this link does not appear, there are no attachment files.]

To ensure your figures meet our technical requirements, please review our figure guidelines: https://journals.plos.org/plosone/s/figures

You may also use PLOS’s free figure tool, NAAS, to help you prepare publication quality figures: https://journals.plos.org/plosone/s/figures#loc-tools-for-figure-preparation.

NAAS will assess whether your figures meet our technical requirements by comparing each figure against our figure specifications.

Attachment Submitted filename: Plos ONE-revised comments.docx

Attachment Submitted filename: PONE-D-25-29935_R1.docx

# Author comment: Author response to Decision Letter 2

**DOI:** 10.1371/journal.pone.0349226.r004

16 Mar 2026

Dear Editor and Reviewers,

We sincerely thank the reviewers for their careful evaluation of our manuscript and for the positive feedback regarding the technical rigor, statistical analysis, and clarity of the work. We appreciate the opportunity to revise the manuscript and have addressed the reviewer comments as outlined below.

Response to Reviewer Comments

We note that Reviewer #2 indicated that all comments had been addressed, and Reviewer #3 provided feedback on the paper as well as a final suggestion requesting clarification of the evaluation model used in the study. In response, we have made the following revisions:

1. Formatting adjustments

The manuscript formatting was revised to improve clarity and alignment with journal requirements. Numerical values previously associated with section headings have been removed to ensure consistency with the journal’s formatting guidelines.

2. Language and spelling corrections

The manuscript was carefully reviewed to address spelling, typographical, and minor grammatical errors. Corrections were made throughout the manuscript to improve readability and ensure the text is presented clearly in standard English.

3. Clarification of the evaluation framework

In response to Reviewer #3’s suggestion to clarify the model used to guide the evaluation, we have added a description of the Context–Input–Process–Product (CIPP) evaluation framework within the Methods section. The framework is now explicitly described as guiding the evaluation of the program context, educational inputs including curriculum and mentorship structures, implementation processes, and program outcomes. Incorporating this framework strengthens the methodological transparency of the study and clarifies the structure used to guide the evaluation and interpretation of findings.

All revisions have been incorporated into the revised manuscript and are reflected within the Methods section.

We thank the reviewers again for their thoughtful feedback and for their positive assessment of the manuscript. We appreciate the opportunity to improve the clarity of our work and believe the revisions have strengthened the methodological transparency and presentation of the study. We

hope the revised manuscript is now suitable for publication in PLOS ONE and we look forward to your consideration.

Sincerely,

Rebecca Silvers

On behalf of all authors

Attachment Submitted filename: PLOS ONE Letter March 2026.pdf

# Peer review: Decision Letter 2

**DOI:** 10.1371/journal.pone.0349226.r005

**Authors:** Alejandro Torrado Pacheco, Douglas Aninng Opoku, Douglas Aninng Opoku

27 Apr 2026

Pediatric Capacity Building in Northern Laos: An Evaluation of the Pediatric Nursing Training Program at Lao Friends Hospital for Children

PONE-D-25-29935R2

Dear Dr. Silvers,

We’re pleased to inform you that your manuscript has been judged scientifically suitable for publication and will be formally accepted for publication once it meets all outstanding technical requirements.

Within one week, you’ll receive an e-mail detailing the required amendments. When these have been addressed, you’ll receive a formal acceptance letter and your manuscript will be scheduled for publication.

An invoice will be generated when your article is formally accepted. Please note, if your institution has a publishing partnership with PLOS and your article meets the relevant criteria, all or part of your publication costs will be covered. Please make sure your user information is up-to-date by logging into Editorial Manager at Editorial Manager® and clicking the ‘Update My Information' link at the top of the page. For questions related to billing, please contact billing support.

If your institution or institutions have a press office, please notify them about your upcoming paper to help maximize its impact. If they’ll be preparing press materials, please inform our press team as soon as possible -- no later than 48 hours after receiving the formal acceptance. Your manuscript will remain under strict press embargo until 2 pm Eastern Time on the date of publication. For more information, please contact onepress@plos.org.

Kind regards,

Douglas Aninng Opoku, MPH

Academic Editor

PLOS One

Additional Editor Comments (optional):

Reviewers' comments:

Reviewer's Responses to Questions

**Comments to the Author**

1. If the authors have adequately addressed your comments raised in a previous round of review and you feel that this manuscript is now acceptable for publication, you may indicate that here to bypass the “Comments to the Author” section, enter your conflict of interest statement in the “Confidential to Editor” section, and submit your "Accept" recommendation.

Reviewer #2: All comments have been addressed

**********

2. Is the manuscript technically sound, and do the data support the conclusions?

The manuscript must describe a technically sound piece of scientific research with data that supports the conclusions. Experiments must have been conducted rigorously, with appropriate controls, replication, and sample sizes. The conclusions must be drawn appropriately based on the data presented.

Reviewer #2: Yes

**********

3. Has the statistical analysis been performed appropriately and rigorously?

Reviewer #2: Yes

**********

4. Have the authors made all data underlying the findings in their manuscript fully available?

The PLOS Data policy requires authors to make all data underlying the findings described in their manuscript fully available without restriction, with rare exception (please refer to the Data Availability Statement in the manuscript PDF file). The data should be provided as part of the manuscript or its supporting information, or deposited to a public repository. For example, in addition to summary statistics, the data points behind means, medians and variance measures should be available. If there are restrictions on publicly sharing data—e.g. participant privacy or use of data from a third party—those must be specified.

Reviewer #2: Yes

**********

5. Is the manuscript presented in an intelligible fashion and written in standard English?

PLOS ONE does not copyedit accepted manuscripts, so the language in submitted articles must be clear, correct, and unambiguous. Any typographical or grammatical errors should be corrected at revision, so please note any specific errors here.

Reviewer #2: Yes

**********

6. Review Comments to the Author

Please use the space provided to explain your answers to the questions above. You may also include additional comments for the author, including concerns about dual publication, research ethics, or publication ethics. (Please upload your review as an attachment if it exceeds 20,000 characters)

Reviewer #2: Thank you for the opportunity to review this manuscript again. At this point, I no longer have any further comments for the authors.

**********

7. PLOS authors have the option to publish the peer review history of their article (what does this mean?). If published, this will include your full peer review and any attached files.

If you choose “no”, your identity will remain anonymous but your review may still be made public.

**Do you want your identity to be public for this peer review?**  For information about this choice, including consent withdrawal, please see our Privacy Policy.

Reviewer #2: No

**********

Attachment Submitted filename: PLOS ONE_Laos.docx

# Editor report: Acceptance letter

**DOI:** 10.1371/journal.pone.0349226.r006

**Authors:** Alejandro Torrado Pacheco, Douglas Aninng Opoku, Douglas Aninng Opoku

PONE-D-25-29935R2

PLOS One

Dear Dr. Silvers,

I'm pleased to inform you that your manuscript has been deemed suitable for publication in PLOS One. Congratulations! Your manuscript is now being handed over to our production team.

At this stage, our production department will prepare your paper for publication. This includes ensuring the following:

* All references, tables, and figures are properly cited

* All relevant supporting information is included in the manuscript submission,

* There are no issues that prevent the paper from being properly typeset

You will receive further instructions from the production team, including instructions on how to review your proof when it is ready. Please keep in mind that we are working through a large volume of accepted articles, so please give us a few days to review your paper and let you know the next and final steps.

Lastly, if your institution or institutions have a press office, please let them know about your upcoming paper now to help maximize its impact. If they'll be preparing press materials, please inform our press team within the next 48 hours. Your manuscript will remain under strict press embargo until 2 pm Eastern Time on the date of publication. For more information, please contact onepress@plos.org.

You will receive an invoice from PLOS for your publication fee after your manuscript has reached the completed accept phase. If you receive an email requesting payment before acceptance or for any other service, this may be a phishing scheme. Learn how to identify phishing emails and protect your accounts at https://explore.plos.org/phishing.

If we can help with anything else, please email us at customercare@plos.org.

Thank you for submitting your work to PLOS ONE and supporting open access.

Kind regards,

PLOS ONE Editorial Office Staff

on behalf of

Dr. Douglas Aninng Opoku

Academic Editor

PLOS One
